# Diagnostic accuracy of keystroke dynamics as digital biomarkers for fine motor decline in neuropsychiatric disorders: a systematic review and meta-analysis

**DOI:** 10.1038/s41598-022-11865-7

**Published:** 2022-05-11

**Authors:** Hessa Alfalahi, Ahsan H. Khandoker, Nayeefa Chowdhury, Dimitrios Iakovakis, Sofia B. Dias, K. Ray Chaudhuri, Leontios J. Hadjileontiadis

**Affiliations:** 1 Department of Biomedical Engineering, Khalifa University of Science and Technology, P O Box 127788, Abu Dhabi, United Arab Emirates; 2 Healthcare Engineering Innovation Center (HEIC), Khalifa University of Science and Technology, P O Box 127788, Abu Dhabi, United Arab Emirates; 3 Department of Electrical and Computer Engineering, Aristotle University of Thessaloniki, 54124 Thessaloniki, Greece; 4 CIPER, Faculdade de Motricidade Humana, Universidade de Lisboa, Cruz Quebrada, 1499-002 Lisbon, Portugal; 5 Parkinson’s Foundation Centre of Excellence, King’s College Hospital NHS Foundation Trust, Denmark Hill, London, SE5 9RS United Kingdom; 6 Department of Basic and Clinical Neurosciences, Institute of Psychiatry, Psychology & Neuroscience, King’s College London, De Crespigny Park, London, SE5 8AF United Kingdom

**Keywords:** Neuroscience, Psychology, Biomarkers, Diseases, Neurology, Signs and symptoms, Engineering

## Abstract

The unmet timely diagnosis requirements, that take place years after substantial neural loss and neuroperturbations in neuropsychiatric disorders, affirm the dire need for biomarkers with proven efficacy. In Parkinson’s disease (PD), Mild Cognitive impairment (MCI), Alzheimers disease (AD) and psychiatric disorders, it is difficult to detect early symptoms given their mild nature. We hypothesize that employing fine motor patterns, derived from natural interactions with keyboards, also knwon as keystroke dynamics, could translate classic finger dexterity tests from clinics to populations in-the-wild for timely diagnosis, yet, further evidence is required to prove this efficiency. We have searched PubMED, Medline, IEEEXplore, EBSCO and Web of Science for eligible diagnostic accuracy studies employing keystroke dynamics as an index test for the detection of neuropsychiatric disorders as the main target condition. We evaluated the diagnostic performance of keystroke dynamics across 41 studies published between 2014 and March 2022, comprising 3791 PD patients, 254 MCI patients, and 374 psychiatric disease patients. Of these, 25 studies were included in univariate random-effect meta-analysis models for diagnostic performance assessment. Pooled sensitivity and specificity are 0.86 (95% Confidence Interval (CI) 0.82–0.90, I^2^ = 79.49%) and 0.83 (CI 0.79–0.87, I^2^ = 83.45%) for PD, 0.83 (95% CI 0.65–1.00, I^2^ = 79.10%) and 0.87 (95% CI 0.80–0.93, I^2^ = 0%) for psychomotor impairment, and 0.85 (95% CI 0.74–0.96, I^2^ = 50.39%) and 0.82 (95% CI 0.70–0.94, I^2^ = 87.73%) for MCI and early AD, respectively. Our subgroup analyses conveyed the diagnosis efficiency of keystroke dynamics for naturalistic self-reported data, and the promising performance of multimodal analysis of naturalistic behavioral data and deep learning methods in detecting disease-induced phenotypes. The meta-regression models showed the increase in diagnostic accuracy and fine motor impairment severity index with age and disease duration for PD and MCI. The risk of bias, based on the QUADAS-2 tool, is deemed low to moderate and overall, we rated the quality of evidence to be moderate. We conveyed the feasibility of keystroke dynamics as digital biomarkers for fine motor decline in naturalistic environments. Future work to evaluate their performance for longitudinal disease monitoring and therapeutic implications is yet to be performed. We eventually propose a partnership strategy based on a “co-creation” approach that stems from mechanistic explanations of patients’ characteristics derived from data obtained in-clinics and under ecologically valid settings. The protocol of this systematic review and meta-analysis is registered in PROSPERO; identifier CRD42021278707. The presented work is supported by the KU-KAIST joint research center.

## Introduction

Motor abnormalities, a transdiagnostic domain of an array of neurological and psychiatric disorders that begin years if not decades before clinical diagnosis^1^, stem from perturbed brain networks involving cognitive, emotional and motor domains^2,3^. Despite their well-established neurobiological mechanisms and clinical criteria, early diagnosis remains a devastating obstacle against effective, disease-modifying treatment and sustained quality of life. In fact, the progression of motor symptoms to warrant clinical diagnosis usually occurs after substantial neural loss in neurodegenerative disorders, and at advanced stages of psychiatric disorders. In the case of Parkinson’s Disease (PD), for instance, the hallmark symptoms of bradykinesia, rigidity and tremor are detected after a neural loss of at least 50%^4^, rendering clinical diagnosis accuracy unsatisfactory at early stages as per a recent meta-analysis^5^. In addition, Alzheimer’s disease (AD) is preceded by a mild cognitive impairment (MCI) stage, characterized by a decline in memory and executive functions that is hardly distinguishable from normal aging, but with pronounced impact on the activities of daily life^6^. In psychiatric disorders, the descriptive nature of clinical scales lacks sensitivity to subtle psychomotor symptoms, either in early or remission stages, resulting in a median delay in diagnosis of 14 years after disease onset^7^. Generally, these diseases, affecting the frontal cortical and subcortical circuits are characterized with executive dysfunction that begins years before diagnosis^1^, entailing the need for dimensional, fine-grained behavioral measures, thereby alleviating the “floor-ceiling” effect associated with qualitative clinical scales as well as the inter- and intra-rater diagnosis variability.

According to the scientific vision (2025) of the Brain Research through Advancing Innovative Neurotechnologies (BRAIN) of the National Institute of Health (NIH)^8^, and the Research Domain Criteria (RDoC) of National Institute of Mental Health (NIMH)^9^, automated behavioral quantification, analysis and classification are a crucial start to high-throughput readout of brain activity, whose impact is envisioned to facilitate breakthroughs in early identification and disease management in both neurology and psychiatry. Concurrent with the ever-increasing interest in behavioral measures, is the lack of hypothesis-supported behavioral experiments^10^. The latter require not only experimental design, but also robust computational and analysis methodologies, supported by clinical ground truth and neurobiological theories. With the booming of smartphones in recent years, keyboard typing became an everyday habit, reflecting unique behavioral profile for every user^11^. We hypothesize that the kinetic movement of fingers during keyboard/touchscreen typing embeds features related to subtle decline in motor sequencing and force steadiness^12^. These are referred to as Neurological Soft Signs (NSS), sub-clinical motor abnormalities that can serve as early “warning signs” of brain dysfunction, and additional clinical evaluation remains essential for precise diagnosis^13–15^.

Besides the passive acquisition of user-device interactions, the intricate Artificial Intelligence (AI) and Machine Learning (ML) methods allowed the definition of new disease-related features^16,17^, resulting in a new class of digital biomarkers, that of keystroke dynamics. We found that the latter provide a rich space of the assessment parameters, similar to that of finger tapping tests that quantitatively score the frequency and speed of tapping in clinical settings, either in single or alternating fashion^13^. Therefore, employing keystroke dynamics for fine motor analysis facilitates a paradigm shift from conventional, subjective diagnosis to objective, in-the-wild assessment. As opposed to other papers in the area of digital phenotyping that provide an overview of an “island of experts”, we hereby concentrate on a specific digital biomarker class with plausible connection to neurobiological mechanisms and clinical workflow. In fact, keystroke dynamics were used for PD and MCI, yet, and to our best knowledge, no systematic reviews and/or meta-analysis attempted to convey their diagnostic potential or their clinical significance for identifying patterns with plausible connections to disease characteristics.

In this systematic review and meta-analysis, we aimed to appraise the diagnostic performance of keystroke dynamics for an array of neurological and psychiatric disorders. Moreover, we sought to assess the impact of data collection settings, labeling methods, and model characteristics on the diagnostic performance, with emphasis on clinical relevance and ecological validity. In the meta-analysis, we provided a quantitative evaluation of the keystroke dynamics diagnosis of PD, MCI and psychiatric disorders independently, to convey their reproducibility and clinical impact. More importantly, we performed regression analysis, to convey the relationship between patients’ demographic and clinical characteristics with the diagnostic potentiality of keystroke dynamics, as well as the derived fine motor impairment index. Lastly, due to the immature progress of this area towards clinical adoption, we cast-in-concrete a detailed, multidisciplinary agenda for all stakeholders involved in the digital biomarker research, and open an avenue to multidisciplinary intervention and care delivery in neurology and psychiatry.

## Results

**Figure 1:**
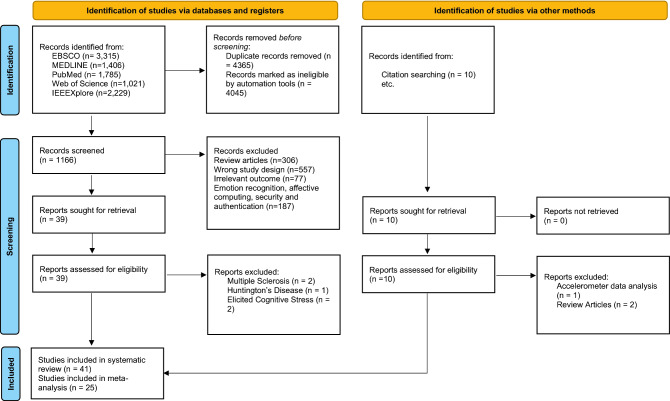
PRISMA 2020 flow diagram for study selection.

Our search identified 9576 results of which 4365 were removed as duplicates and 4045 were excluded by automation tools, as illustrated by the PRSIMA 2020 flowchart in Fig. 1. We therefore screened the title and the abstract of 1166 articles, and we identified 1120 as not meeting our eligibility criteria. Thirty-nine (39) full eligible articles were screened and from their list of references, we identified seven more articles that meet our eligibility criteria. From the resulting 46 articles, five full articles, listed in supplementary file (p. 7) were excluded. At the end of our systematic search, we ended up with 41 full articles of which 25 reported sufficient data to be included in the meta-analysis. Overall, 25 studies are targeting PD, ten studies targeting mood disorders, and six studies were on mild cognitive impairment and AD. The characteristics of the included studies are summarized in Table 1 and are discussed in the following section.

**Table 1:** Characteristics of included studies.

Author (year)	Disease	Data set characteristics and experimental protocol				Data processing			Problem formulation		Funding
		Collection settings	#patients (avg age, SD, %female)	# controls (avg age)	Labeling method	Data streams	Analysis level	Extracted features	Statistical analysis	Classification	
Memedi et al. (2013)^30^	PD	In-the-wild (36 months)	65 (65, 11, 33.8%)	10 (61, 7, 50%)	Hoehn and Yahr scales, UPDRS, visual evaluation of the tapping pattern	Tap position (x–y pixel coordinates) and time-stamps (in milliseconds)	Subject level	Total tapping time and speed, features derived using dynamic time warping and zero-crossing signals to assess typing regularity and accuracy	Linear mixed effects models with maximum likelihood estimation (long-term analysis)	Logistic regression	Swedish Knowledge Foundation
Printy et al. (2014)^26^	PD	In-the-clinic	18 (68.5, 12.1, 44.4%)	NA	UPDRS-III, clinical assessment of upper limb kinematics	Boolean values describing screen contact, tri-axial gyroscope and accelerometer data	Subject level	Tapping frequency (# taps/5 s with 50% overlapping windows), tapping rhythmicity (amplitude peak frequency of normalized power spectral density), tapping rhythmicity (coefficient of variation (CV) of between-tap intervals, CV of finger contact time)	NA	Support vector machines and random forests	NR
Giancardo et al. (2016)^18^	PD	In the clinic	42 (59.0, 9.8, 43%)	43 (60.1, 10.2, 60%)	Clinical evaluation: UPDRS, Alternating Finger Tapping, Single Finger Tapping tests	Keystroke timing data	Session level	HT variance features (outliers, skewness, and finger coordination) and HT probability features (histograms bins)	NA	Support vector regression	Comunidad de Madrid, Fundacion Ramon Areces and The Michael J Fox Foundation for Parkinson's research (grant number 10860)
Vesel et al. (2020)^58^	Bipolar disorder	In-the-wild (8 weeks)	NR	250 (37.7, 12.25, 70%)	Self-reported Patients Health Questionnaire (PHQ-8 )	Keyboard metadata including consecutive time stamps of key presses, character, punctuation, backspace, autocorrect rates	Session level	Inter-key delay	Hierarchical growth curve mixed-effects models		Mood Challenge for Research kit 1R01MH120168
Giancardo et al. (2015)^64^	Psychomotor impairment	In-the-wild (NA)	NA	14 (30.8)	Self-reports	Keystroke timing data	Session level	Hold time evolution matrix, its peak and self-similarity	Rayleigh test for circular uniformity	Linear support vector machines	Comunidad de Madrid, Fundacion Ramon Areces and The Michael J Fox Foundation for Parkinson's research (grant number 10860)
Mastoras et al. (2019)^63^	Depression	In-the-wild (2 months)	11 (23.6, 3.24, 36.36%)	14 (23.8, 4.44, 42.86%)	Self-reported Patients Health Questionnaire (PHQ-9 )	Keyboard interactions including consecutive time stamps of key presses, typing meta-data including session duration and number of special characters	Subject level	Low- and high order statistics of the HT, NFT, normalized pressure and typing speed (inter-key distance/NFT)		Best performing model: random forests	Al Jalila Foundation 2017 Research Grants
Zulueta et al. (2018)^56^	Bipolar disorder	In-the-wild through “BiAffect” Smartphone Application (8 weeks)	9 (48.7, 9.63, 89%)	NR	Hybrid (clinical assessment: HDRS, YMRS/frequent mood self-reports)	Keystroke meta-data, accelerometer data, mobile use activity	Subject level	Avg. accelerometer displacement, IKD, Backspace ratio, avg. session length, number of sessions, circadian baseline similarity	Mixed effects regression	NA	Mood Challenge for Research kit 1R01MH120168
Stange et al. (2018)^57^	Bipolar disorder	In-the-wild through “BiAffect” smartphone application (10 weeks)	18 (NR)	NA	Hybrid (clinical assessment: HDRS, YMRS/ecological momentary assessment)	Keyboard meta-data	Subject level	Root mean square successive difference (rMSSD) between keystrokes	Multi-level and boot-strapped mediation analysis	NA	Mood Challenge for Research kit 1R01MH120168
Vizer et al. (2015)^49^	MCI	In-the-clinic (4 typing sessions, 20–45 min each)	17 (81.12, 6, NR)	20 (79.24, 6, NR)	Clinical evaluation: mini mental state examination (MMSE)	Keystroke timing data and their linguistic content	Subject level	Paralinguistic: pause rate and duration, time per key and keystroke rate and linguistic features: sentence complexity, rate of nouns, verbs and adjectives	NA	Logistic regression	US National Science Foundation graduate research fellowship, and the US National Library of Medicine Biomedical and Health Informatics Training Program at the University of Washington (grant number T15LM007442)
Ntracha et al. (2020)^50^	MCI	In-the-wild (6 months)	11 (67.2, 5.96, 81.8%)	12 (66.2, 4.72, 58.3%)	Clinical Assessment (SCI, MMSE, FUCAS, FRSSD)	KD and texts simulating Spontaneous Written Speech (SWS)	Subject level	NLP features and R/B/AFT indices from KD	NA	kNN (KD alone, logistic regression (NLP alone), ensemble model (fused features)	Horizon 2020 research and innovation programme under grant agreement No 690494—i-PROGNOSIS
Matarazzo et al. (2019)^34^	PD	In-the-wild (6 months)	30 (63.00, NR, 48.3%)	29 (59.78, NR, 53.3%)	Clinical assessment	HT	Subject level	HT distribution matrix	NA	RNN	Michael J. Fox Foundation for Parkinson's Research Grant 10860
Pham et al. (2018)^20^	PD	In-the-clinic (NA)	42 (59.0, 9.8, 43%)	43 (60.1, 10.2, 60%)	Clinical assessment (UPDRS-III, alternating/single finger tapping tests)	HT	Session Level	Recurrence plots and scalable network features	NA	Support vector machines	NR
Pham et al. (2019)^21^	PD	In-the-clinic (NA)	42 (59.0,9.8, 43%)	43 (60.1, 10.2, 60%)	Clinical assessment (UPDRS-III, alternating/single finger tapping tests)	HT	Session Level	Recurrence plots and scalable network features	NA	Long-short term memory (LSTM)	NR
Iakovakis et al. (2018)^19^	PD	In-the-clinic (NA)	18 (61, 8.4, 22%)	15 (57, 3.9, 46%)	Clinical assessment (UPDRS-III)	Time stamps of key presses and releases	Subject level	High and low order statistics of HT, normalized FT and normalized pressure	NA	Two stage ML pipeline (best performing: random forest and mean voting)	Horizon 2020 research and innovation programme under grant agreement No 690494—i-PROGNOSIS
Iakovakis et al. (2018)^32^	PD	In-the-wild (52 weeks)	13 (62, 6, 38%)	35 (57, 8, 40%)	Self-reports	Time stamps of key presses and releases	Subject and session level	NA	NA	Subject and typing session level Regression model for severity estimation	Horizon 2020 research and innovation programme under grant agreement No 690494—i-PROGNOSIS
Iakovakis et al. (2020)^37^	PD	In-the-wild (NR)	TS1: 22 (58.6, 8.4, 22%), TS2: 9 (de novo) (56, 8, 33%) TS3: 67 (61, 7, 35.8%)	TS1, TS2: 17 (54.6, 9.4, 41%) TS3: 186 (58.7, 7.5, 36%)	DB1, clinical evaluation, DB2 self-reports	Keystroke timing data	Subject level	NA	NA	Hybrid deep learning model based on data in-the-clinic and in-the-wild	Horizon 2020 research and innovation programme under grant agreement No 690494—i-PROGNOSIS
Papadopoulos et al. (2020)^36^	PD	In-the-wild (NR)	DB1: 14 PD (60.7, 9.8, 27.3%), DB2: 26, (60.7, 8.9, 64.1%)	DB1:8 (50.5, 9, 50%), DB2: 131 (54.5, 10, 41.98%)	DB1; clinical evaluation, DB2; self-reports	Typing and tri-axial accelerometer data	Subject level	Independent feature transformer for typing and accelerometer data	NA	Deep learning	Horizon 2020 research and innovation programme under grant agreement No 690494—i-PROGNOSIS
Chen et al. (2019)^51^	MCI	In-the-wild (3 months)	MCI: 24 (69.0, 1.8, 54%)/AD: 7 (72.1, 3.5, 57%)	82 (66.3, 0.8, 71%)	Clinical assessment conducted using the National Institute of Aging-Alzheimer’s Association	Accelerometer, pace, stride, heart rate, sleep cycle, distance from home, workout sessions, breathing sessions, standing hours, exercise minutes, phone calls, apps, sleep stages, steps, mood/energy surveys, tapping tests	Subject level	Tapping speed, tapping regularity, typing speed, sentence complexity, drag path efficiency, and reading times	NA	Extreme gradient boosting	NR
Arroyo-Gallego et al. (2017)^24^	PD	In-the-clinic (NA)	21 (59.24, 11.43, 52%)	23 (54.3, 13.95, 83%)	Clinical assessment (UPDRS-III, alternating/single finger tapping tests)	NFT	Session level	Skewness, kurtosis, covariance of NFT time series	NA	Best performing model: SVM	Comunidad de Madrid, Fundación Ramón Areces, and The Michael J Fox Foundation for Parkinson’s research (grant number 10860)
Prince et al. (2018)^31^	PD	In-the-wild (6 months)	312 (63.8, 6.8, NR)	86 (61.9, 7.7, NR)	Self-reports using digitized UPDRS	timestamps (time of finger touching the screen) and the x,y screen pixel coordinates for each tap instance	Subject level	Progression rate and steady state indexes	Spearman’s correlation	NA	Digital Economy Programme grant number EP/G036861/1 (Oxford Centre for Doctoral Training in Healthcare Innovation)
Lipsmeier et al. (2018)^35^	PD	In-the-wild (6 months)	43 (57.5, 8.45, 18.6%)	35 (56.2, 7.8, 22.9%)	Clinical assessment (UPDRS)	Sustained phonation, rest tremor, postural tremor, finger tapping, balance, gait	Subject level	Features correspond to tasks in order: mel-frequency cepstral coefficient, skewness, total power, intra-tap variability, mean velocity, turn speed	Mann Whitney, linear-effects mixed models	NA	F. Hoffmann-La Roche Ltd. and Prothena Biosciences Inc
Stringer et al. (2018)^52^	MCI	In-the-clinic (NA)	20 (75.60, 5.78, 30%)	24 (71.09, 5.38, 58%)	Clinical assessment using ACE-III, ECog scores	Computer use behavior (keyboard and mouse)	Subject level	Typing speed and pausing frequency	NA	Regression	The Engineering and Physical Sciences Research Council (EPSRC) under Grant EP/K015796/1
Rabinowitz et al. (2014)^54^	MCI	In-the-clinic (NA)	170 (82.1, 6.2, 51.2%)	NA	Clinical assessment (MMSE, recall, digit span test)	Finger tapping signal (via pressure transducer)	Subject level	Mean, SD, coefficient of variation of the HT and the FT, mean and SD of HT/tapping period ratio	The Kruskal–Wallis test, t test, Mann–Whitney U test	LDA and SVM	NR
Waes et al. (2017)^53^	MCI	In-the-clinic (NA)	12 (73.9, 4.3, NR)	20 (22.5, 1.0, NR), 20 (74.3, 5.8, NR)	Clinical assessment (Petersen’s diagnostic criteria), MMSE, GDS	Time stamps of keystroke loggings	Subject level	Inter-key latency	MANOVA	NA	The University of Antwerp Research Fund; the Alzheimer Research Foundation
Lee et al. (2016)^22^	PD	In-the-clinic (NA)	57 (65.4, 9, 60.4%)	87 (53.4, 14.8, 60.9%)	Clinical assessment (UPDRS; sub-scores of motor, bradykinesia, rigidity, postural instability and gait disturbance, UK brain bank)	Number of taps (correct taps and tap errors), inter-tap distance and total finger distance	Subject level	Mean and variance (1st order statistics)	Means of the continuous variables compared using t-test or Mann Whitney test. Univariate analysis for the determination of the impact of age, sex, asymmetry and hand dominance	Linear regression	Hallym University Research Fund (HURF-2015-34)
Arora et al. (2018)^23^	PD	In-the-clinic (NA)	334 (66.1, 9, 37%)	84 (66.3, 9.1, 33%)	Clinical assessment	7 smartphone tasks assessing voice, balance, gait, finger tapping, reaction time, rest tremor, and postural tremor	Subject level	Vocal fold excitation ratio, tapping rhythm, pitch, acceleration	NA	Random forests (RF)	Digital Economy Programme grant number EP/G036861/1 (Oxford Centre for Doctoral Training in Healthcare Innovation)
Arora et al. (2018)^23^	Idiopathic REM sleep disorder	In-the-clinic (NA)	104 (64.5, 9.4, 12%)	84 (66.3, 9.1, 33%)	Clinical assessment	7 smartphone tasks assessing voice, balance, gait, finger tapping, reaction time, rest tremor, and postural tremor	Subject level	Vocal fold excitation ratio, tapping rhythm, pitch, acceleration	NA	Random forests (RF)	Digital Economy Programme grant number EP/G036861/1 (Oxford Centre for Doctoral Training in Healthcare Innovation)
Zhan et al. (2016)^39^	PD	In-the-wild (6 months)	121 (57.6, 9.1, 41%)	105 (45.5, 15.5, 47%)	Self-reports	Tri-axial accelerometer data, tasks assessing voice, balance, gait, finger tapping, and reaction time	Subject level	Higher and lower order statistics for voice, gait, and tapping parameters	NA	Random forests (RF)	NR
Wissel et al. (2017)^27^	PD	In-the-clinic (NA)	11 (60.6, 9, 27.3%)	11 (62.5, 11, 55%)	Clinical assessment (MDS-UPDRS-III during ON and OFF states)	Timestamps of taps, pixel locations	Subject level	The total number of taps, tap interval (time [ms] between two consecutive finger/hand screen taps), tap duration (time [ms] the index finger/hand touches the screen per tap), and tap accuracy (tap distance [pixels] from the center of the target) were recorded	T test/correlation analysis	NA	NR
Adams et al. (2017)^28^	PD	In-the-clinic (NA)	32 (NR, NR, NR)	71 (NR, NR, NR)	Clinical assessment (UPDRS)	Keystroke timing information (preprocessed as n tuples)	Subject level	Mean, skewness and kurtosis of hold time and key latency (left and right differences were considered to assess symmetry)	NA	Ensemble machine learning classification models	NR
Milne et al. (2018)^29^	PD	In-the-clinic (NA)	42 (59.0, 9.8, 43%)	43 (60.1, 10.2, 60%)	Clinical evaluation: UPDRS, AFT, SFT	Keystroke timing information	Subject level	Mean and SD, mean absolute consecutive difference of the HT, features extracted using feature extraction based on scalable hypothesis (FRESH)	NA	Logistic regression	NR
Arroyo-Gallego et al. (2018)^33^	PD	In-the wild (2 months)	25 (60.2, 12.0, 48%)	27 (60.8, 10.6, 52%)	Clinical assessment (UPDRS)	Keystroke timing information	Subject level	neuroQWERY index	NA	Support vector regressor	Comunidad de Madrid, Fundación Ramón Areces, and The Michael J Fox Foundation for Parkinson’s research (grant number 10860)
Huang et al. (2018)^60^	Bipolar disorder	In-the-wild (2 months)	Bipolar 1: (45.6, 9.9, 57%), bipolar 2: 5 (52.4, 9.4, 80%)	8 (46.1, 107, 63%)	The Hamilton Depression Rating Scale (HDRS) and Young Mania Rating Scale (YMRS), daily self-reports	Keystroke timing data, alphanumeric data, accelerometer data	Subject level	HT, FT, and pixel coordinates, tri-axial accelerometer	NA	Stacked convolutional and recurrent neural networks (CNN-RNN)	NSF through grants IIS-1526499, IIS-1763325, and CNS-1626432, and NSFC 61672313
Cao et al. (2019)^59^	Bipolar disorder	In-the-wild (2 months)	Bipolar 1: 7 (45.6, 9.9, 57%), bipolar 2: 5 (52.4, 9.4, 80%)	8 (46.1, 10.7, 63%)	The Hamilton Depression Rating Scale (HDRS) and Young Mania Rating Scale (YMRS), daily self-reports	Keystroke timing data, alphanumeric data, accelerometer data	Subject level	HT, FT, and pixel coordinates, tri-axial accelerometer, auto-correct, backspace, space rate	NA	Multi-layer gated recurrent units (GRUs)	NSF through grants IIS-1526499, IIS-1763325, and CNS-1626432, and NSFC 61672313
Iakovakis et al. (2019)^38^	PD	In-the-wild (NR)	27 (NR)	84 (NR)	Self-reports	Keystroke timing data	Subject level	NA	NA	CNN	Horizon 2020 research and innovation programme under grant agreement No 690494—i-PROGNOSIS
Wang et al. (2021)^40^	PD	In-the-wild (NR)	8 (60.5, 9.2, 37.5%)	8 (23.6, 3.7, 62.5%)	Clinical assessment	Keyboard touchpoints (as pixels) and keystroke timing data	Session level	Text entry speed (words per minute), typing error, unintentional repetitive touch	Elastic probabilistic model	NA	National Key R&D Program of China under Grant No. 2019YFF0303300, the Natural Science Foundation of China under Grant No. 62002198, No. 61902208
Goni et al. (2021)^42^	PD	In-the-wild (NR)	970 (59.85, 9.05, 35%)	1630 (46.84, 10.05, 15.2%)	Clinical assessment	Smartphone application with 4 tasks: gait, balance, voice and tapping	Subject level	700 features extracted, comprising statistical features of time and frequency locomotion	NA	Least absolute shrinkage and selection operator (LASSO), RF, SVM	NR
Surangsrirat et al. (2022)^41^	PD	In-the-wild (NR)	1851 (44.27, 0.44, 31.5%)	NA	Self-reports	Demographics, MDS-UPDRS I–II, PDQ-8, memory, tapping, voice, and walking	Subject level	High and low order statistics of keystroke dynamics	NA	K-means unsupervised clustering	National Science and Technology Development Agency (NSTDA), Thailand
Zulueta et al. (2021)^62^	Bipolar disorder	In-the-wild (35 months)	227 (35, 11, 75%)	117 (41, 16, 60%)	Self-reports	Keystroke dynamics and typing metadata (autocorrect and backspace rate)	Session level	Low order statistics of keystroke dynamics, entropy (complexity) features	NA	RF	Mood Challenge for Research kit 1R01MH120168
Ross et al. (2021)^61^	Bipolar disorder	In-the-wild (2 months)	11 (47, 10.6, 72.7%)	8 (46.1, 10.6, 62.5%)	Hybrid (clinical assessment and self-reports)	Keystroke timing data	Session level	Low-order statistics	Longitudinal mixed effects	NA	The Heinz C. Prechter Research Program; Richard Tam Foundation; Michigan Institute for Clinical and Health Research, Grant/Award Number: UL1TR002240

NR—not reported; NA—not applicable.

### Characteristics of included studies

Of the 41 included studies, we identified 25 on PD with 3791 patients of whom 33.9% were female, six on MCI and early AD with 254 patients of whom 52.4% were female, and ten on psychiatric disorders with 374 patients of whom 56.0% were female (not all studies reported gender information). Regardless of the target condition and the data collection setting (in-the-clinic, in-the-wild), typing patterns, or keystroke dynamics, are always passively collected as series of time stamps of consecutive key presses and releases. The derived kinematic parameters are then used for motor behavior pattern analysis.

Of the PD studies, 12 were conducted in-the-clinic^18–29^, while 13 were conducted in-the-wild^30–42^. The earliest studies, that were mainly on PD, collected data in clinical settings, and attempted to correlate the extracted keystroke dynamic features to the Unified Parkinson’s Disease Rating Scale Part III (UPDRS-III) score, which is currently the gold standard for PD diagnosis^43^. On this basis, PD patients were found to have longer inter-key delay, also known as Flight Time (FT), smaller number of total taps (over a fixed tapping duration), and shorter total distance of finger movement compared to controls^22^. Keystroke dynamics analysis also showed that PD patients are characterized by arrhythmokinesia, that is, hastening or freezing in the typing kinetics^44^, as well as heteroscedasticity or dispersion of FT^24^.

Owing to the establishment of reproducible digital biomarkers on the basis of keyboard interaction patterns, the neuroQWERTY index, for example, was estimated using an ensemble regression model that digests variance and histogram features extracted from 90 s windows of the hold time (HT) series obtained from early stage PD patients^18^. The HT, which is the time required for pressing and releasing a key, was particularly employed in early studies given that it is neither affected by the typing skill nor by conscious control. Consequently, the numerical index derived from it, neuroQWERTY, did not only discriminate early-stage PD patients from controls, but also de novo PD patients, reflecting its high sensitivity to subtle motor changes. Besides the HT, the flight time (FT), the latency between releasing a key and pressing the next one was analyzed in^24^ to test the hypothesis that PD patients are characterized by higher dispersion and temporal variability compared to controls. The analysis of the typing patterns of PD patients through the neuroQWERTY keyboard revealed their slower fine-motor kinetics as well^40^. Compared to the Alternating Finger Tapping (AFT) test, employing skewness, kurtosis and covariance features of the FT distribution resulted in a higher diagnosis accuracy, meaning that the typing patterns embed specific irregularities of PD motor symptoms, mainly attributed to rigidity and bradykinesia. In an effort to enrich the feature space of keystroke dynamics, Iakovakis et al.^19^ developed a two-stage machine learning model based on low- and high-order statistical features derived from the HT, Normalized FT and Normalized Pressure. Their results were consistent with earlier studies, and showed higher and more variable HT, lower pressure and high FT skewness. While these features were significantly correlated to the motor sub-scores of the UPDRS-III, correlating the outcome of such standardized clinical scales, which encompass a mixture of symptoms not related to fine motor impairments, to the typing behavior might be misleading. Taking this into consideration and with the objective of enhancing the interpretability of the fine-grained indicators, Iakovakis et al.^32^ analyzed keystroke dynamics with single items scores of the UPDRS Part III, in order to create a plausible connection between the typing behavior and fine motor impairment symptoms. Employing typing kinetics features as independent variables, the UPDRS single items that correspond to the severity of Bradykinesia, Tremor, Rigidity, and AFT were estimated. The regression results indicated that dominant hand rigidity and bradykinesia were estimated with lower error compared to tremor, meaning that the effect of the latter is less pronounced from the typing cadence.

Furthermore, the “transferability” of typing patterns-based models developed and tested on clinically validated data to naturalistic, quasi-continuous, self-reported data from daily interaction with keyboards was evaluated. While the models achieved higher diagnostic performance in clinical settings, they still show high potentiality for real-life detection of disease-induced abnormal behavior^32,33,36–38^. Moreover, exploiting the passive nature of typing data acquisition, data from 970 PD patients, part of the mPower database^45^, facilitated the detection of early motor decline through Support Vector Machine and Random Forests^42^. Similarly, using the mPower database, unsupervised clustering of smartphone tapping data was used to discriminate the severity of motor symptoms in PD^41^.

Given that amalgamating multiple data streams in one model boosts its diagnostic accuracy, multimodal analysis has been uniquely adopted by Papadopolous et al*.*^36^ in order to achieve symptom-specific detection, wherein accelerometer data are used to yield a tremor estimation index, while the typing behavior is leveraged for estimating fine motor impairment. Besides diagnosis, five longitudinal clinical studies investigated medication response^27,30,31,34,39^ with the longest follow up being 36 months^30^. For instance, typing behavior has been utilized to detect longitudinal disease phenotype to uncover short- and long-term variations in the motor behavior profile of PD patients as in^31^. This was achieved by the definition of reliable parameters, such as the progression ratio and the steady state ratio, derived by comparisons between motor behavior across consecutive time windows. While this aspect is still in its infancy, Matarazzo et al.^34^ showed promising results in detecting response to *levodopa* using recurrent neural networks. This implication, in turn, suggests that deep learning is a robust predictive model in biomarkers research, and is therefore being used in five clinical studies^21,34,36–38^.

Besides PD, Growing evidence, from studies targeting imaging biomarkers, suggests that the accumulation of Amyloid β starts up to 20 years before the manifestation of clinical symptoms of Alzheimer’s disease (AD) and that this is detected in one third of the clinically normal elderly population^46^. Whether this population will convert to AD, and at what time frame remain elusive, entailing the search for quantitative assessments during this preclinical stage. AD is in fact preceded by a mild cognitive impairment (MCI), which is an intermediate stage characterized by subtle deficits in memory, lexical and information processing, besides sensory and motor abnormalities^47,48^. In particular, fine motor impairment has been linked to functional loss at the MCI stage, and is specifically compromising the performance of daily life activities. Therefore, six studies were identified on MCI and AD^49–54^. The validity of utilizing the typing kinetics as biomarkers for early stage cognitive decline came about after the pioneering experimental trials that attempted to replicate finger dexterity tests in naturalistic environments. Specifically, the inter-keystroke interval, which is the FT, showed promising cognitive assessment performance of the elderly population^55^. This is particularly linked to breakdowns in attentional control and short-term memory, which constitute two key domains of time-reproduction tasks, such as typing. On this basis, increased latency variability and slower performance were observed in MCI and dementia patients, as compared to age- matched healthy participants^54^. Therefore, capturing computer-use profiles, including mouse and keyboard interactions successfully discriminated MCI patients from age-matched healthy controls^52^.

Interestingly, the multi-domain dysfunction of the prefrontal cortex motivated the development of multi-modal assessment methods, to validate the co-existence of motor and cognitive impairment. The sharp degradation of lexical processing and syntactic complexity reflects on MCI-specific language characteristics including increased verb and pronoun rate and decreased noun rate. To this end, Vizer and colleagues combined keystroke timing features including the HT and the pause rate with linguistic features collected in clinical settings to distinguish PreMCI subjects from age matched healthy controls^49^. Taking the analysis a step further, with the advancement in Natural Language Processing (NLP), and the capability of capturing objective linguistic features, usually not recognized by human raters, Ntracha et al. employed NLP of Spontaneous Written Speech (SWS), fused with keystroke dynamics features captured in-the-wild, to reinforce the interplay of cognitive and fine motor functions^50^. Furthermore, the pronounced advancement in computational modeling now allows aligning multiple data lines, what facilitated the development of “behaviorgrams” that capture activity levels, physiological and behavioral signals on a longitudinal bases, yielding a more comprehensive overview of individuals’ health, yet without solid interpretability on longitudinal transient behavior^51^.

Of the ten studies targeting psychiatric disorders, we identified seven studies on bipolar disorder^56–62^, one study on idiopathic REM Sleep Behavior disorder^23^, two studies are on depression^63^ and sleep induced psychomotor impairment^64^, respectively. All these studies were conducted in-the-wild except the one on REM sleep disorder^23^. Mental and psychiatric disorders, with major depression being the most prevalent, are the leading cause of the disease burden worldwide, accounting for 32.4% of years lived with disability^65^, and substantially contributing to health loss across the lifespan^66^. The underlying mechanisms of depression include dopaminergic, noradrenergic and serotonergic disturbances along with inflammatory and psychosocial factors^67^. Depression has therefore been identified as an epiphenomenon in PD, MCI and AD patients, and has been linked to higher prevalence of neurodegeneration. As per the recommendations of the National Institute of Mental Health (NIMH), deep phenotyping of disease mechanisms at multiple analysis levels, including genetic, neural, and behavioral levels, is key for early diagnosis and monitoring^9^.

From pathological and clinical perspectives, psychomotor perturbation is a well-defined criterion of manic and depressive states^68^. Stemming from this, keyboard interaction patterns, along with accelerometer data, backspace and autocorrect rate were used a predictor variables in a linear mixed effects model to estimate Hamilton Depression Rating Scale (HDRS) and Young Mania Rating Scale (YMRS) scores of bipolar disorder patients^56^. Besides the psychomotor slowing observed by the longer FT, the analysis of the typing meta data including autocorrect and backspace rate, reflect the cognitive states associated with depressive and manic states. For instance, the high autocorrect rate associated with depressive states reflect the degree of concentration impairment. In contrast, the high backspace rate during manic states is associated with deteriorated error-response inhibition. The impact of circadian rhythm, depression severity, and age also have a profound impact on the typing kinetics^58^. In this vein, the analysis of typing kinetics, along with the clinical scores, facilitated the prediction of brain age and revealed that the predicted age of bipolar disorders patients is higher than their actual age, compared to healthy controls, reflecting a marker of brain pathology^62^. Moreover, keystroke dynamics predict cognitive decline, diminished visual attention, reduced processing speed and task switching in bipolar disorder patients^61^.

Beside these approaches, employing machine learning methods such as random forests yielded high discriminatory performance between mildly and severely depressed patients, and controls, from typing data collected in-the-wild^63^. Considering the impact of individual’s unique typing style and the circadian rhythm, stacking convolutional neural networks that detect personalized features, along with recurrent neural networks that learn the dynamic patterns, resulted in personalized mood detection^59,60^. Taking the analysis a leap forward, leveraging passively acquired keystroke dynamics with day-to-day ecological momentary assessment for mood prediction suggested that higher mood instability, inferred from the self-reports and the typing kinetics are highly predictive of worsening depressive and manic symptoms^57^. They also showed that continuous monitoring for up to seven days is sufficient for accurate symptom prediction using multilevel statistical analysis. The longest follow up period among studies targeting psychiatric disorders was eight weeks^56^.

### Diagnostic potentiality of keystroke dynamics

**Figure 2:**
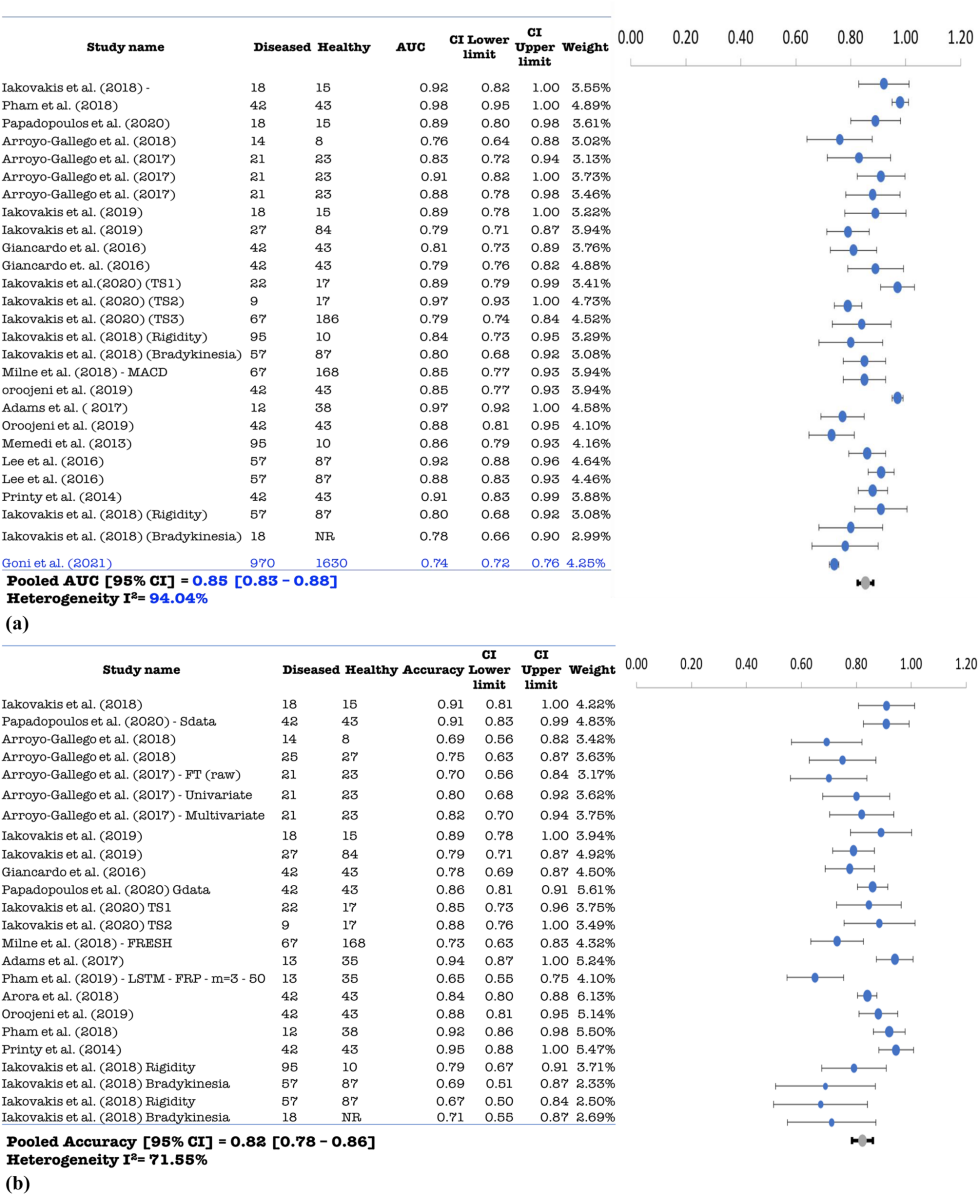

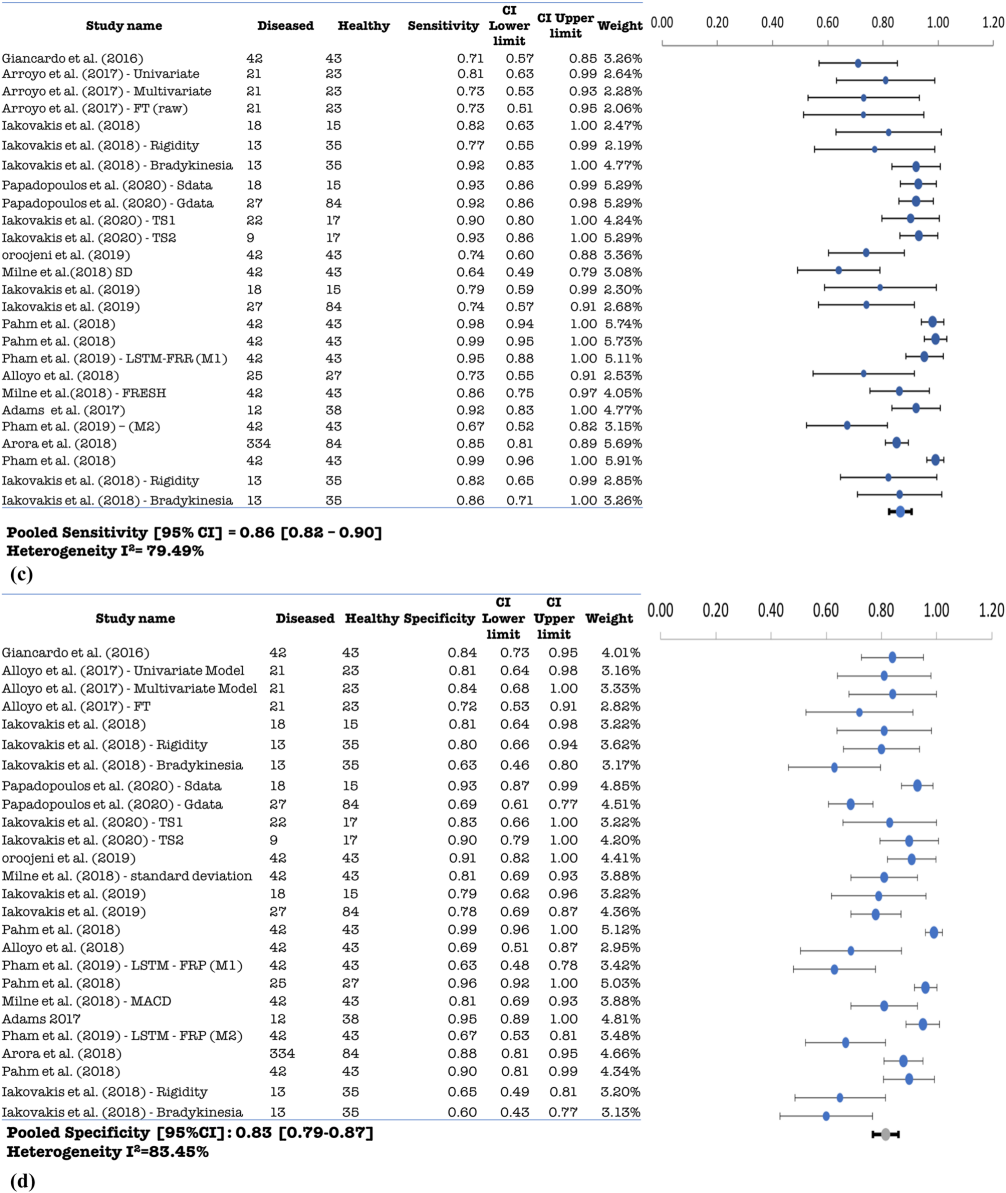
(**a**): Pooled AUC with 95% CI of PD studies. (**b**) Pooled accuracy with 95% CI for PD studies. (**c**) Pooled sensitivity with 95% CI for PD studies. (**d**) Pooled specificity with 95% CI for PD studies.

**Figure 3:**
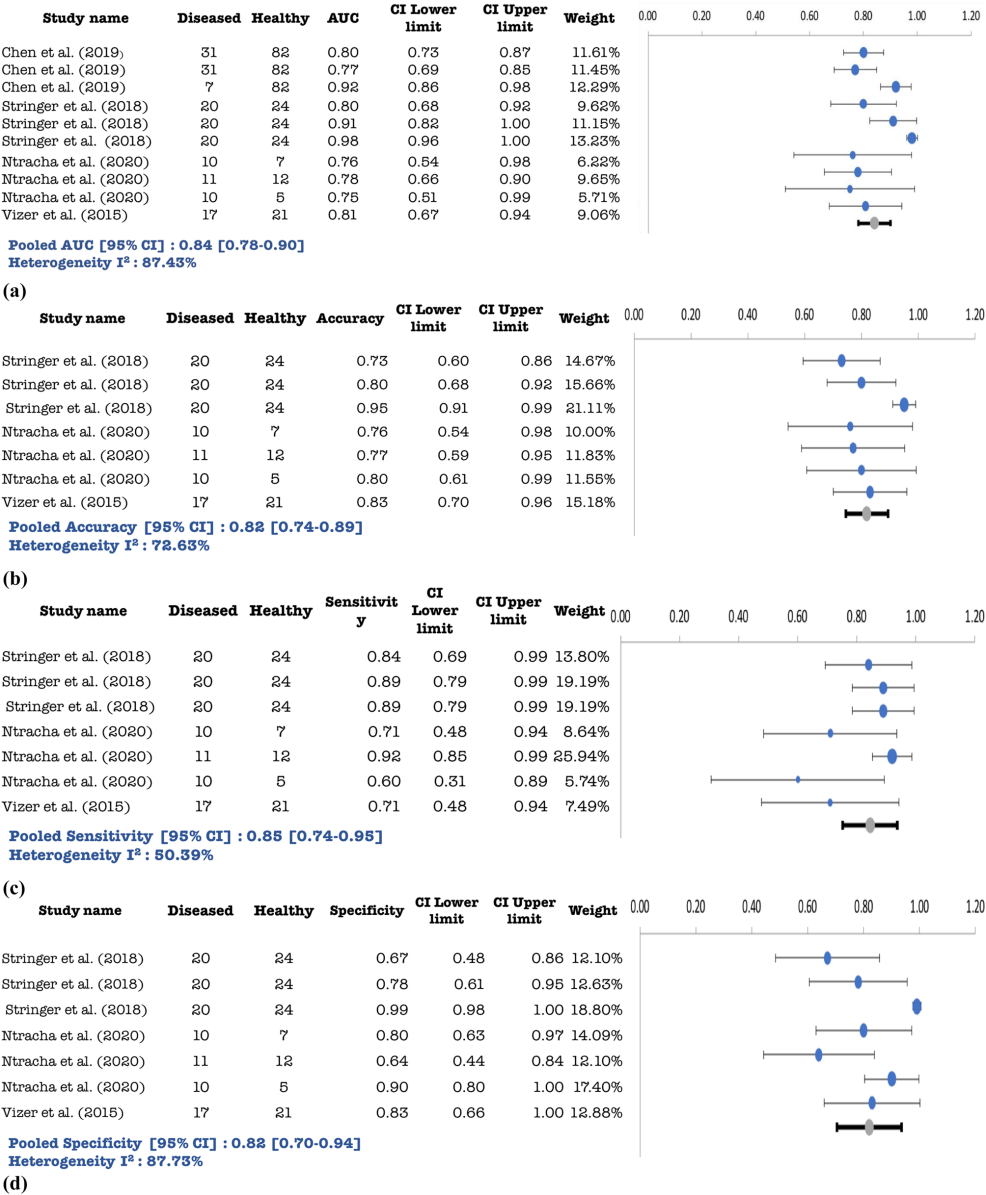
(**a**) Pooled AUC with 95% CI for MCI studies. (**b**) Pooled accuracy with 95% CI for MCI studies. (**c**) Pooled sensitivity with 95% CI for MCI studies. (**d**) Pooled specificity with 95% CI for MCI studies.

**Figure 4:**
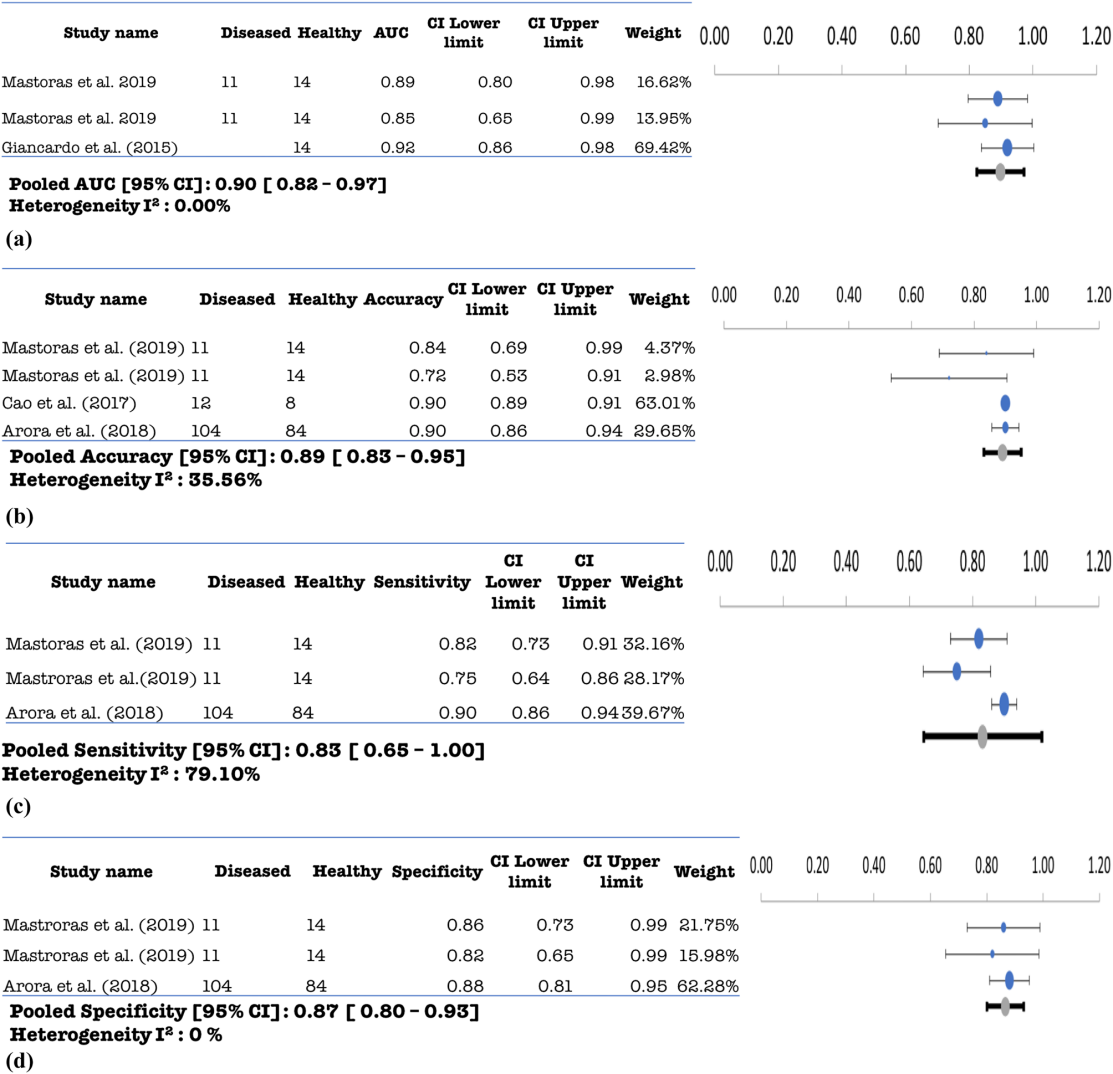
(**a**) Pooled AUC with 95% CI for psychiatric disorder studies. (**b**) Pooled Accuracy with 95% CI for psychiatric disorder studies. (**c)** Pooled Sensitivity with 95% CI for psychiatric disorder studies. (**d**) Pooled Specificity with 95% CI for psychiatric disorder studies.

Twenty-five (25) independent studies were included in the meta-analysis, given that the symmetry condition of the funnel plots is respected (Figs. S1–S4). Whenever possible, if one study formulated multiple models, we treat them independently and their specific characteristics are reported in Supplementary Table 7. We identified 29 independent models for the diagnosis of PD on the basis of keystroke dynamics. Pooled AUC and accuracy of keystroke dynamics classification methods for PD were 0.85 (95% confidence interval (CI): 0.83–0.88; I^2^ = 94.04%) and 0.82 (95% CI 0.78–0.86; I^2^ = 71.55%), respectively. In addition, pooled sensitivity and specificity were 0.86 (95% CI 0.82–0.90, I^2^ = 79.49%) and 0·83 (95% CI 0.79–0.87, I^2^ = 83.45%), as shown in Fig. 2a–d. For MCI and AD (Fig. 3a–d) we found ten independent classification models, except for the study of^51^ that only reported AUC for their three models. The pooled AUC and accuracy were 0.84 (95% CI 0.78–0.90, I^2^ = 87.43%) and 0.82 (95% CI 0.74–0.89, I^2^ = 72.63%), respectively. Pooled sensitivity and specificity for the same category were also found to be 0·85 (95% CI 0.74–0.96, I^2^ = 50.39%) and 0.82 (95% CI 0.70–0.94, I^2^ = 87.73%). We identified four independent models for psychiatric diseases with^59^ only reporting accuracy. Pooled AUC and accuracy for psychomotor impairment were 0.90 (95% CI 0.82–0.97, I^2^ = 0%) and 0.89 (95% CI 0.83–0.95, I^2^ = 35.56%). Pooled sensitivity and specificity for psychomotor impairment were 0.83 (95% CI 0.65–1.00, I^2^ = 79.10%) and 0.87 (95% CI 0.80–0.93, I^2^ = 0%) as shown in Fig. 4a–d. More importantly, the non-significance, inferred by the sensitivity analysis for every disease category, reveals the consistency of the reported diagnostic accuracy, for all pooled measures.

### Assessment of experimental design on diagnostic performance

In order to decipher the heterogeneity sources of the included studies, we have conducted multiple subgroup analyses. Comparing the performance of the diagnostic models when per- formed on data captured in-the-clinic to data captured in-the-wild revealed that the AUC (p = 0.007) and the accuracy (p = 0.032) were significantly higher under clinical settings. The AUC and the accuracy for the data captured in-the-clinic were 0.89 (95% CI = 0.86–0.91, I^2^ = 87.15%, n = 21) and 0.87 (95% CI = 0.83–0.90, I^2^ = 62.33%, n = 18), respectively. The same measures for data captured in-the-wild were 0.82 (95% CI = 0.79–0.84, I^2^ = 74.02%, n = 21) and 0·81 (95% CI = 0.77–0.85, I^2^ = 71.82%, n = 17), respectively. In terms of the sensitivity and the specificity, we found that the pooled sensitivity was not significantly higher for data captured in-the-clinic (p = 0.903), while the specificity was significantly higher for data captured in-the-clinic (p = 0.032). These metrics for data captured in-the-clinic were 0.85 (95% CI = 0.80–0.99, I^2^ = 82.06%, n = 20) and 0.87 (95% CI = 0.83–0.91, I^2^ = 81.81%, n = 18). For data captured in-the-wild, pooled sensitivity and specificity were 0.85 (95% CI = 0.79–0.90, I^2^ = 52.55%, n = 14) and 0.79 (95% CI = 0.73–0.85, I^2^ = 68.96%, n = 16). Similarly, the AUC (p = 0.004), the accuracy (p = 0.013), and the specificity (p = 0.002) are significantly higher for clinically-validated databases, compared to self-reports labeled typing data. For the former, the AUC, accuracy, pooled sensitivity and specificity were 0.86 (95% CI = 0.83–0.89, I^2^ = 86.41%, n = 31), 0.86 (95% CI = 0.83–0.89, I^2^ = 64.17%, n = 26), 0.86 (95% CI = 0.81–0.90, I^2^ = 78.77%, n = 21) and 0.87 (95% CI = 0·0.84–0.91, I^2^ = 73.41%, n = 22). On the other hand, these metrics for the self-reported data were 0.78 (95% CI = 0.73–0.84, I^2^ = 0.00%, n = 6), 0.79 (95% CI = 0.74–0.83, I^2^ = 29.24%, n = 8), 0.83 (95% CI = 0.76–0.90, I^2^ = 50.11%, n = 9) and 0.76 (95% CI = 0.69–0.82, I^2^ = 77.43%, n = 11).

From a methodological point of view, we report no statistical significance between pooled AUC (p = 0.525) and sensitivity (p = 0.074) when we compare unimodal and multimodal analysis methods. The specificity (p = 0.042) and the accuracy (p = 0.022), however, were significantly higher for multimodal analysis. Pooled AUC, accuracy, sensitivity and specificity for multimodal analysis were as follows: 0·83 (95% CI = 0·77–0·90, I^2^ = 90·83%, n = 9), 0·87 (95% CI = 0·83–0·91, I^2^ = 66·24%, n = 11), 0·89 (95% CI = 0·84–0·94, I^2^ = 45·77%, n = 7) and 0.87 (95% CI = 0.79–0·95, I^2^ = 90·19%, n = 8). The same measures for unimodal analysis were 0·86 (95% CI = 0.84–0.89, I^2^ = 76.42%, n = 31), 0.82 (95% CI = 0.78–0.85, I^2^ = 63.98%, n = 25), 0.84 (95% CI = 0.79–0.89, I^2^ = 76.41%, n = 22) and 0.80 (95% CI = 0.76- 0.84, I^2^ = 68.23%, n = 17).

**Figure 5:**
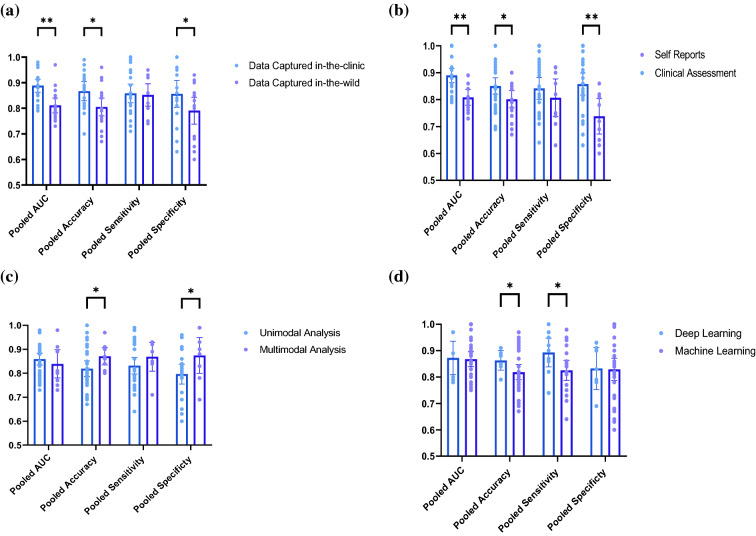
Scatter–Bar plots for the Subgroup Analysis results for (**a**) data collected in-the-clinic vs. data collected in-the-wild, (**b**) clinically validated data vs. self-reported data, (**c**) multimodal analysis vs. unimodal analysis and (**d**) deep learning vs. other machine learning classifiers. The dots represent the individual studies and the height of the bars corresponds to the outcome of the random effects meta-analysis model with 95% CI. ** denotes p < 0.005 and * denotes p < 0.05.

Comparing the performance of ML classifiers and deep learning methods, the sensitivity was significantly higher for deep learning classifiers (p = 0.029), compared to linear machine learning methods, while the AUC (p = 0.859), accuracy (p = 0.299), and specificity (p = 0.882) were all associated with insignificant difference. The pooled AUC, accuracy, sensitivity and specificity for machine learning classifiers were 0.86 (95% CI = 0.83–0.88, I^2^ = 75.29%, n = 33), 0.83 (95% CI = 0.80–0.87, I^2^ = 66.49, n = 26), 0.82 (95% CI = 0.78–0.86, I^2^ = 71.49, n = 24) and 0.83 (95% CI = 0.78–0.87, I^2^ = 75.82%, n = 26), respectively. On the other hand, in the case of deep learning, the pooled measures are 0.86 (95% CI = 0.79–0.94, I^2^ = 86.77%, n = 7), 0·86 (95% CI = 0.81–0.91, I^2^ = 51.77%, n = 8), 0.89 (95% CI = 0.83–0.96, I^2^ = 44.25%, n = 9) and 0.83 (95% CI = 0.76–0.91, I^2^ = 80.37%, n = 9). Figure 5 represents scatter-bar plots of the subgroup analyses results forest plot representations can be found in Figs. (S5–S20).

### Association of diagnostic performance with age and disease duration

**Figure 6:**
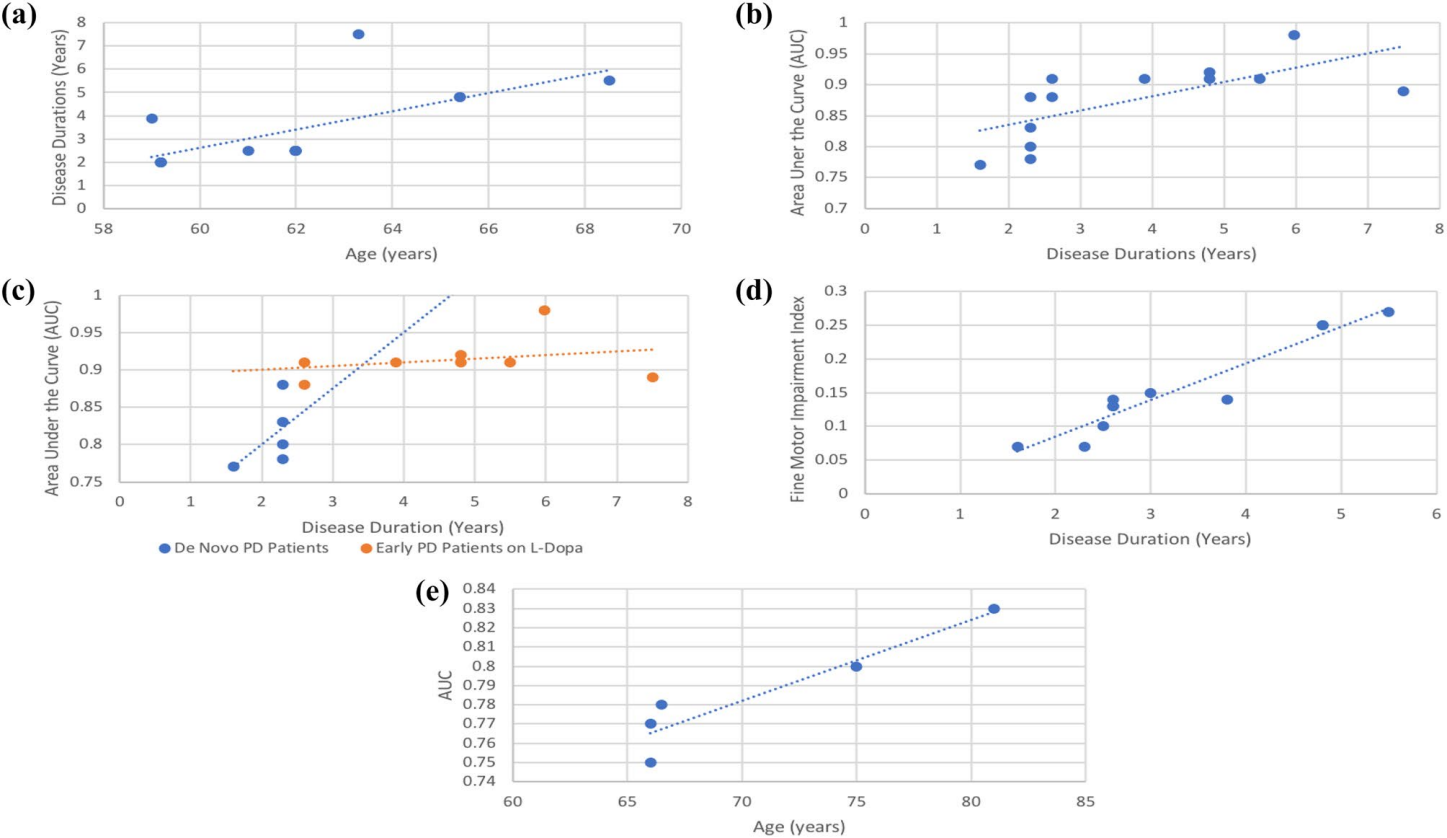
Evaluation of the impact of patients’ age and disease duration on the diagnostic performance of keystroke dynamics represented by the AUC. (**a**) Regression analysis results of PD patients age and years from diagnosis (disease duration). (**b**) Regression analysis results of PD studies reporting diagnostic AUC and disease duration reveals their significant association. (**c**) Pooled AUC of de novo PD patients (blue) and early PD patients on L-Dopa (orange) depicts the sharper increase in AUC with disease duration of de novo PD patients, compared to that of early, medicated PD patients. (**d**) Regression analysis results of Fine motor impairment index derived from the HT and the disease duration. (**e**) Regression analysis results of MCI patients age and diagnosis AUC.

We hypothesize that patients’ demographics and clinical characteristics affect the diagnostic potentiality of keystroke dynamics. To this aim, we performed multiple linear regression analyses to convey the influence of age, disease duration, and medication on the diagnosis potentiality of keystroke dynamics. We also pooled fine motor impairment indexes, mainly related to bradykinesia, to investigate the influence of disease stage on the estimated motor impairment severity. Due to the unavailability of sufficient data for MCI and psychiatric disorders studies, we were mainly able to perform the regression analysis for PD diagnosis. Figure 6a shows the relationship between PD patients’ age and disease duration (years from diagnosis). The figure intuitively suggests that PD disease duration increases with age, and the relationship between the two is statistically significant (p = 0.013) as inferred from the regression analysis. Accordingly, adjusting for disease duration, we analyzed its relationship with diagnostic AUC as represented in Fig. 6b. The regression analysis yielded a statistically significant increase in AUC with disease duration (p = 0.005), reflecting the progression of fine motor impairment skills of PD patients. Next, using the same data, we investigated the AUC relationship with disease duration, when de novo PD patients are compared to early PD patients taking levodopa (l-Dopa). Interestingly, when we use linear fitting to each group, the higher slope associated with the de novo PD patients, compared to that of early PD patients on l-Dopa indicates that although the diagnostic AUC of de novo patients is lower, the evolution of the AUC with respect to disease progression for this patients’ category is more significant, mainly during the first three years after diagnosis, than that of early, medicated PD (Fig. 6c). Perhaps this implication also suggests the sharper decline in fine motor skills at this stage, resulting in a clear improvement in the diagnostic AUC. This is in line with the recent evidence suggesting an exponential neurodegeneration patterns of the Substantia Nigra pars compacta, parallel to a sharper decline in motor skills in early PD^69^. Besides the diagnostic performance, we sought to investigate the association of PD disease duration and the severity of fine motor symptoms. We pooled the fine motor impairment index, that derived from the HT, as an estimation of bradykinesia, as it was reported by multiple studies with sufficient data. However, not all studies reported the fine motor impairment index derived from the HT. Figure 6d depicts the significant correlation (p = 0.010) between the disease duration and fine motor impairment index.

Although the included studies on MCI were generally few compared to those targeting PD, we were able to perform regression analysis to convey the diagnostic potentiality relationship with patients’ age. As represented in Fig. 6e, there is a significant increase in the diagnostic AUC of MCI based on fine motor skills inferred by keystroke dynamics (p = 0.017). The full regression results are reported in Supplementary Tables 9–12.

### Evaluation of between-study heterogeneity and bias risk

The large between-study heterogeneity made combining data from multiple studies to generate a representative effect size on the diagnosis performance problematic. It is due to this reason that we decided to pool four diagnostic metrices via univariate random-effect meta-analysis models. Consequently, we assume that pooled diagnosis metrics of PD, MCI, and psychiatric disorders, as well as the subgroup analysis results, are adequate to convey the diagnostic potentiality of keystroke dynamics models and the impact of study characteristics; namely data collection settings, labeling methods, and the modeling characteristics. Hence, we group the studies based on the desired outcome and assume that despite the heterogeneity, the pooled outcome contributes to the evidence. For instance, when evaluating the diagnostic performance for every disease category, the heterogeneity stems from the between-study differences in experimental design and model characteristics, however, when we group the studies based on experimental characteristics despite the disease category, we attribute the heterogeneity to patients’ characteristics, and other experimental design aspects that are not under investigation. Furthermore, we reinforce our findings from the global performance of studies by evidence from methodological perspectives. Nonetheless, we still caution against overinterpretation.

**Figure 7:**
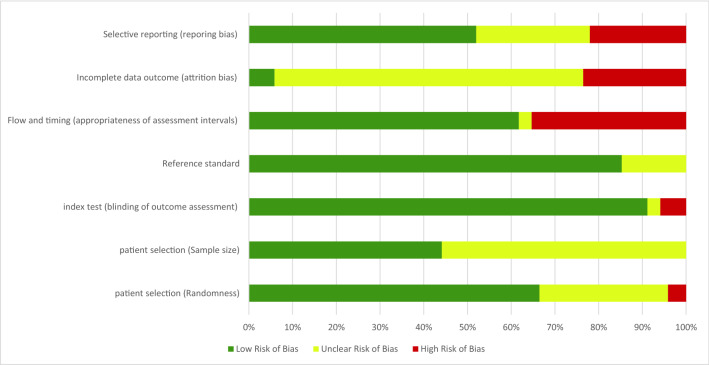
Risk of bias assessment.

Figure 7 shows the graphical representation of the risk of bias of included studies, and the per-study risk of bias assessment is reported in Supplementary Table 6. Given that we target the diagnostic accuracy, the included studies are case–control including a priori labeled diseased and healthy participants. We consider the studies that labeled the participants using self-reports without clinical evaluation at high risk of bias, because participants’ honesty, recall bias and unawareness of their medical conditions influence the correctness of the labels. Furthermore, most studies did not assess the appropriateness of the sample size, we therefore deemed this of unclear risk of bias for most studies, except 12 studies that aimed at enlarging the sample pool, mainly collecting data outside clinics. Besides, we deemed all the studies that performed independent clinical evaluation and keystroke dynamics analysis (outcome assessment blindness) to be of low risk of bias, except^30,39^, that did finger dexterity and clinical evaluation of PD without blindness. All studies were characterized with low risk of bias when we consider timing of ground-truth labeling and data collection. Most studies are of unclear risk of bias in terms of selective reporting. Overall, we deemed the risk of bias to be low to moderate, and the quality of evidence, as inferred by the GRADE tool, to be moderate to high, as illustrated in Supplementary Table 8.

## Discussion

To our best knowledge, this is the first systematic review and meta-analysis that provides a concentrated overview of the clinically-relevant diagnostic performance of keystroke dynamics, their ecological validity and association with patients’ demographics and clinical characteristics. We found that most studies are targeting PD, given its hallmark motor symptoms, however, there are now multiple studies dedicated to the assessment of motor perturbation in MCI, bipolar disorder, and depression. The diagnostic accuracy revealed by our meta-analysis reflects, for the first time, the reproducibility of keystroke dynamic models in the assessment of multiple disorders with neurologically defined fine motor impairment. Besides the three disease categories reviewed in this paper, researchers are currently employing them for Multiple Sclerosis^70,71^ and Huntington’s disease^72^. We therefore conclude that we can rely on keystroke dynamics obtained passively from natural interactions with keyboards to detect fine motor impairments induced by early stage neurological and/or psychiatric disorders. Despite this diagnostic performance, several experimental and analysis deficiencies need to be discussed to mitigate between-study heterogeneity, pave the way for future research. For clinical adoption of this technology, we propose a partnership strategy based on a “co-creation” approach that stems from mechanistic explanations of patients’ characteristics derived from data obtained in-clinics and under ecologically valid settings. It is the multi-level analysis of patients’ data on genetic-, organ-, and behavior-level that will be at the center of the translational paradigm to precision medicine when the heterogeneous brain disorders are to be considered.

While computer/smartphone interaction behavior outperformed clinical gold standards such as the AFT and the single finger tapping tests in detecting specific fine motor symptoms of PD patients, the transition from highly-controlled assessment in-the-clinic, to naturalistic, real-life assessment models should be approached with caution^73–75^. From the sampling perspective, home-based data collection usually results in highly sparse bursts of unpredictable typing activity, that are highly sensitive to real-life contexts, emotional burden and diurnal patterns. This is in line with the higher discriminatory performance of the models on data captured in-the-clinic and labeled by clinical assessment, elucidated by our subgroup analysis. Therefore, to establish robust detection models for diagnosis outside clinics, integrating multiple latent domains, or confounders and defining multiple predictor parameters, such as emotions, activity levels, and sleep patterns, is particularly an interesting avenue for future research to enhance ecological validity^76^. Such integrated frameworks might therefore capture the heterogeneous, neuropsychiatric symptoms in different behavioral disorders, let alone the intra-subject variability that occurs across different time windows. Moreover, because there is neither a consensus on the optimal assessment duration to detect meaningful disease trajectories and progression of neurological disorders, nor for episodic relapse in psychiatric disorders, long-term analysis of behavioral profiles is essential. Moreover, optimizing the analysis window length, that is, the distribution of observation period, to precisely detect disease-induced transient behavior is yet to be performed.

The inherent, progressive nature of psychiatric and neurodegenerative disorders makes them amenable to frequent treatment regimen modifications, yet satisfying symptom control is not achieved given the high economic burden of clinical visits^77^. Besides screening and diagnosis, the concept of remote monitoring is realized thanks to the passive acquisition of high frequency, objective behavioral data. While this undoubtedly constitutes a promising arena, the lack of standardization objective features and the inconsistent analysis methods remain a challenge^78^. A contributing factor to this, according to^76^, is the short assessment time and the rare outcome assessment during the study duration. To be more precise, the ground truth clinical evaluations that are performed at intermittent intervals during longitudinal data acquisition results in many unlabeled days, therefore the validity of propagating these labels for long time windows is still unclear. Perhaps undertaking a hybrid labeling approach combining low frequency clinical assessment and higher frequency Ecological Momentary Assessment via self-reports along the study duration might therefore mitigate this dilemma.

As illustrated earlier in our subgroup analysis, the adoption of deep learning methods that efficiently extract meaningful patterns from unstructured data is now on the rise. However, deep learning methods that outweighed the rest of machine learning models in terms of diagnostic accuracy are associated with considerable uncertainty. Interestingly, with the aim of enhancing the efficacy of remote assessment of PD, Iakovakis et al*.*^37^ combined two databases captured in-the-clinic and in-the-wild in a deep learning, hybrid model capable of learning fine motor symptoms, thereby overcoming the induced quantization error of the UPDRS-III and enhancing the performance of deep learning. Similarly, our meta-analysis showed that multimodal analysis, although reinforces the diagnostic accuracy, is characterized with considerable diagnostic between-studies uncertainty, therefore, future studies should adopt a more transparent and well-conducted study designs to reduce bias. Perhaps combining voice analysis techniques along with keystroke dynamics will boost the detection of early motor impairment signs, as these are also reflected on speech characteristics of PD patients^79^. Moreover, from the methodological perspectives, several pattern recognition tools have the potential to learn and decipher the nonlinear, dynamic nature of human-keyboard interactions. For example, fuzzy recurrence plots and scalable recurrence networks visually revealed finer texture and more regularity in the hold time series of healthy controls to early stage PD patients^20,21^.

Psychiatric and neurodegenerative disorders that develop and progress across the lifespan are characterized by a heterogeneous phenotype of motor and non-motor symptoms^80^. Early stage behavioral perturbations constitute a priori link with plausible connection to disease likelihood, but the high cross-talk between symptoms obscures accurate diagnosis and pathogenesis understanding especially at preclinical stages. This heterogeneity is a central problem to diagnostic research, entailing additional methods for analyzing similarities and differences across disease-induced behavioral disturbances. As opposed to previous reviews that put too much emphasis on specific disorders, we hereby deliberately included studies on PD, MCI and affective disorders to convey that the neurobiological mechanisms differ greatly among disorders that are characterized with similar traits, such as motor slowing. Rather than focusing on specific disorders in isolation of others, we advocate a dimensional approach that stresses more on the symptoms per se, also referred to as comorbidities. We believe that a central challenge, in this realm, is formulating databases with a full representation of the population, to expand our understanding of the heterogeneous disease-related traits.

Although the previous years witnessed an increase lean towards digital health technologies, the premature adoption of these measures by clinics precludes meaningful outcome^81^. Our work highlights important directions for future research. The definition of clinically meaningful thresholds is yet to established, and this cannot be attained without a “co-creation” approach, whereby high-level data and clinically validated interpretations are made. For instance, amalgamating low level, behavioral data, with high level imaging data is not explored yet. We think that this will not only inform better health information, but might also generate new knowledge on “brain fitness” and behavior, across the generations. Further, the importance of interdisciplinary interactions also propagates to ethical implications, for enhanced transparency, informed consent from patients, privacy and accountability^82^. We therefore summarize domain-specific limitations and future research directions in Table 2.

**Table 2:** Future directions for the digital biomarkers research based on the “co-creation approach”.

Domain	Stakeholders	Limitations	Recommendations
Surveillance/screening	Clinicians Researchers	lack of benchmarking databases with a whole representation of the population; overfitting of the models, and the inability to pinpoint disease-specific phenotypes, and shared symptoms between the disorders	Enlarging clinically validated databases; mapping digital, behavioral data with disease-specific mechanisms across neurological and psychiatric disorders
Diagnosis	Clinicians Researchers Patients	Lack of data interpretability; high sensitivity to contextual content	Identify high-risk populations; identify behavioral patterns that are not associated with disease (inflection points) by analyzing latent domains; fine tune sensitivity and specificity of models; encourage patients to seek early medical diagnosis
Monitoring	Clinicians Researchers Patients	Lack of robust dynamic analysis methods; lack of meaningful behavioral profiles that indicate prognosis and symptoms fluctuation; difficulties in data alignment	Develop multimodal, deep learning models that digests temporal, dense behavioral data; analyze behavioral trajectories that reflect disease progression
Prediction	Researchers	Lack of explanations and trust towards digital health technology	Employ deep learning methods, such as restricted Boltzman machines, for behavioral modeling and prediction
Real-time feedback	Clinicians Researchers Patients	Absence of robust risk assessment models; ambiguous relationships of behavioral trajectories associated with disease progression and those not related to health; lack of patients’ education about the value of medical technology	Generating interpretations of longitudinal behavioral change and linking them to genetic and organ level function for better understanding of disease-induced transitions; designing high- throughput, computationally efficient risk assessment models that runs in real-time; educating patients about the merit of personalized digital technology and its role in improving quality of life
Behavioral intervention	Clinicians Researchers Patients	Lack of personalized behavioral change platforms for digital rehabilitation	Correlate symptoms and disease severity with lifestyle requirements such as exercise intensity and frequency; employ virtual reality for the design of collaborative serious games
Ethics	Clinicians Researchers Ethical regulatory frameworks	Security and transfer issues with individuals’ personal data	Secured data repositories (Cloud); obtain patients’ consent in a transparent way

We acknowledge that our study has several limitations. Among them is the sparsity and the inherent heterogeneity of the included studies. While we were able to perform regression analysis with patients’ demographic and clinical characteristics (i.e., age, disease duration respectively) for PD, our meta-analysis lacks the investigation of additional covariates, such as gender differences and medication response, especially for MCI and psychiatric disorders. Although promising results have been revealed by leveraging typing patterns for diagnosing and monitoring mood and cognitive decline, the majority of the studies are, so far, disproportionately targeting PD. While this is understandable given the hallmark motor disturbance in this latter, we see that the need for further validations of this approach in other disorders is still pressing. This will be an important avenue for future studies. The data collected and analyzed in the included studies are collected either in the United States (US) and Europe, therefore, future clinical trials of the diagnostic performance of keystroke dynamics in other populations, with possibly lower education level and smartphone usage, particularly in ageing populations and low-income countries are needed. Perhaps also a global consortium on the translation possibility of this technology to these populations with limited neurological care access is the first step in this context. We can therefore investigate how the diagnostic potentiality changes across time, by site and for different populations. Concerning per-patient variability and disease progression, future work should be more focused on identifying temporal symptom profiles and behavioral trajectories indicative of conversion to brain disease. More importantly, latent domains such as emotions, sleep pattern should be considered as confounders, given their direct influence on motor behavior and general health status. Estimation of motor impairment severity, that correlates with disease stage and subtype is also an important future avenue. Furthermore, future researchers in the field should collaborate with clinicians to make the models more interpretable, thereby enhancing clinical adoption. Research on the area of explainable AI (XAI) is now rapidly growing^83^, but collaborative work between data scientists, engineers and clinicians is not yet established, especially in mutual exchange of data (i.e., behavioral data, imaging). Eventually, we declare, as a limitation, that the protocol of this systematic review and meta-analysis is registered in PROSPERO, but has not been published yet.

Lastly, we note the strength of our meta-analysis conclusions that conveyed the feasibility of using keystroke dynamics derived from the natural interaction connected devices keyboards as digital biomarkers for early decline in fine motor skills associated with neuropsychiatric disorders. Based on experimental design comparisons, we showed that the keystroke dynamics constitute an ecologically valid diagnostic platforms *in-the-wild*, reflecting their translational potentiality outside clinics, despite the methodological challenges that arises, including but limited to confounders influence and sampling difficulties. Further, given the influence of data labeling on the diagnosis models, we conclude that even when self-reported data *in-the-wild* are used for training, keystroke dynamics models still achieve sound discriminatory potential. From methodological perspectives, we show that employing multimodal and advanced deep learning models, which are at the high edge of the contemporary data science methodologies, offer promising opportunities for boosting the diagnostic accuracy, but with considerable heterogeneity across the studies. Consequently, the establishment of intricate and generalizable diagnostic models, that not only achieve accurate diagnosis, but are also sensitive to temporal change and symptom progression. To this end, our regression models showed the evolution of diagnosis AUC and fine motor impairment with age and disease duration for PD. We reperformed the regression analysis for MCI, and showed how the diagnostic AUC increases with age, reflecting the increasing fine motor impairment severity. In conclusion, the importance of digital technology also goes beyond the diagnostic yield, so once at-risk cohorts are identified, digital technologies can also be employed to reinforce behavior change and patients’ empowerment, towards a sustained quality of life, as detailed in Table 2.

## Methods

### Search strategy and selection criteria

In this systematic review and meta-analysis, conducted in accordance with the Diagnostic Test Accuracy extension of Preferred Reporting Items for Systematic Reviews and Meta- Analyses (PRISMA-2020)^84^, a systematic search of MEDLINE, PubMed, IEEE Xplore, Web of Science, and EBSCO has been independently performed by two authors (H.A and N.C) for publications between January 1st, 2010 and March 30th, 2022, on pattern recognition and neuropsychiatric disease classification on the basis of natural interactions with keyboards, without language restrictions. These date restrictions were specified a priori, because typing patterns constitute a new class in the fruitful digital phenotyping area. The full search strategy of all databases is reported in Supplementary Tables 1–5 and in the PRISMA 2020 checklist. Eligible studies assessed the influence of motor impairment induced by psychiatric or neurological disorders on the typing patterns (i.e., keystroke dynamics). Those deemed eligible were case–control studies, comparing the typing behavior of neuropsychiatric disease patients to age- and education-matched healthy control subjects. Studies that used statistical analysis without classification were included in the narrative synthesis, while those employing machine learning models for classification were included in a random effects meta-analysis to evaluate the diagnosis performance on the basis of typing behavior. We performed a manual search of the reference lists from the eligible studies, and we searched the grey literature for unpublished data, conference proceedings and dissertations. Prior to the writing of this paper, we searched if there are existing systematic reviews and meta-analyses on the same topic.

All search results were uploaded to Rayyan web of intelligent systematic reviews^85^ for duplicates removal and screening. One author (H.A.) screened titles and abstracts of the included studies, that were double-screened by a second author (L.H.). Three authors (H.A., A.K. and L.H.) assessed the eligibility of the included full articles. Any disagreement was resolved by discussion.

### Protocol registration

The protocol of this systematic review and meta-analysis has been registered in PROSPERO with identifier CRD42021278707.

### Data extraction and quality assessment

Two authors (H.A. and L.H.) extracted data from the included studies. We extracted the following data from the included studies: (1) disease, (2) first author and publication year, (3) experimental protocol of data collection including collection settings and study duration, (4) number and mean age of participants in diseased and healthy groups, (5) data labeling methodology (self-reported meta-data vs. clinical evaluation), (6) data streams employed by the study, (7) extracted features, (8) analysis and feature extraction level (subject- level vs. typing session-level), (9) problem formulation and validation whether through statistical analyses or classification, (10) 2 × 2 data (True Positives, True negatives, False Positives, False Negatives), and from here we extracted the sensitivity and the specificity (11) Classification Accuracy and (12) Area Under the Receiver Operating Characteristics Curve (AUC). Three authors (H.A., A.K. and L.H.) discussed and assessed the quality of the included studies. The studies that did not perform classification, were included in the systematic review but not in the meta-analysis.

### Statistical analysis and diagnosis evaluation

Our primary outcome is the diagnosis efficiency of machine learning models employing typing features (i.e., keystroke dynamics). Secondary outcomes include longitudinal disease monitoring on the basis of pattern recognition of keystroke dynamics, treatment response, and key features that discriminate diseased from healthy groups.

In particular, the outcomes of the meta-analysis were the Area Under the receiver operating characteristic Curve (AUC), accuracy, sensitivity and specificity. These outcomes were pooled and included in a univariate random effect model independently for three disease categories, namely PD, MCI, and psychiatric disorders. Heterogeneity was assessed using the I^2^ statistics, attributable to non-sample related between-studies differences, in addition to the Cochran Q (X^2^) test (p < 0·05). Given that in this study we report the validity of keystroke dynamics models as diagnostic tools for different disorders, we accepted high heterogeneity (I^2^ > 50%). Furthermore, to ensure the completeness and transparency of the reported diagnostic accuracy measures, we followed the Standards for the Reporting of Diagnostic Accuracy Studies (STARD)^86^.

After pooling the data, we processed them using the Meta Essential tool^87^. For each study, we entered the (per-subject) AUC and the accuracy and the sample size, while for the sensitivity and specificity, we entered the number of participants in diseased and healthy groups, respectively. These measures, along with the 95% confidence interval (CI), were represented by univariate forest plots. All included studies, that reported AUC, accuracy, sensitivity and specificity were included in the meta-analysis given that we maintain symmetry of the funnel plots to minimize publication bias. Studies that did not report any of these measures and/or were associated with high bias risk were included in the systematic review but not in the meta-analysis. Furthermore, for each of the three disorder groups, we performed a sensitivity analysis using leave-one-study-out, to investigate the impact of individual studies on the diagnostic metrics. Two authors (H.A. and L.H.) performed and agreed on the performance and the outcome of the statistical analysis.

### Subgroup analysis

Subgroup analyses were conducted to assess the source of heterogeneity between the studies, if each subgroup contained more than three studies (n > 3) after subgroup division. We particularly focus on the performance of data acquisition and analysis methods, given that they are the main intellectual challenges of the highly fertile arena of digital phenotyping^73^. In spite of the increasing interest in real-life diagnosis, we segregated the studies based on the data acquisition modality as (1) *in-the-clinic* and *in-the-wild*. Furthermore, we compared the attained AUC, accuracy, sensitivity and specificity between (2) clinically validated and self-reported data. In addition, comparisons between (3) multimodal and unimodal studies, as well as (4) deep learning and other machine learning classification methods were performed.

### Regression analysis

Four linear regression models were fitted for (1) PD patients’ age and years from diagnosis (disease duration), (2) PD diagnosis AUC and disease duration, (3) PD fine motor impairment index and disease duration and (4) MCI diagnosis AUC and patients’ age. These tests were two-sided with a statistical significance threshold of 0.05 and 95% CI.

### Publication bias assessment

Publication bias was assessed based on Begg and Mazumdar’s rank correlation test and visualized by funnel plots. Importantly, if one database was used in multiple studies, or if one study employed multiple analysis methods, we treat those as independent studies.

To assess the internal validity of the included studies, quality assessment was performed employing the tool for Quality Assessment of Diagnostic Test Accuracy (QUADAS-2)^88^. All discrepancies were resolved by mutual discussions between three authors (H.A., A.K., and L.H.). Moreover, we generated four funnel plots for AUC, accuracy, sensitivity and specificity to visually illustrate the publication bias of the included studies^89^.

### Quality of evidence assessment

To convey the clinical value of keystroke dynamics, we have used the Grades of Recommendations, Assessment, Development and Evaluation (GRADE) tool^90^ to systematically and transparently assess the diagnostic accuracy evidence of keystroke dynamics for neuropsychiatric disorders. The systematic appraisal of the evidence quality is determined by (1) the design of the study, (2) risk of bias, (3) inconsistency of reported results, (4) indirectness of the outcome, (5) imprecision of the reported results, and (6) publication bias.

## Supplementary Information

Supplementary Information 1.

Supplementary Information 2.

## Data Availability

The search strategy and extracted data contributing to the meta-analysis is available in the appendix; any additional data are available on request from the corresponding author.
