# Supplementary material for: Diagnostic accuracy of keystroke dynamics as digital biomarkers for fine motor decline in neuropsychiatric disorders: a systematic review and meta-analysis
Source: Sci Rep. 2022 May 11;12:7690. doi: 10.1038/s41598-022-11865-7 (PMC9095860; doi:10.1038/s41598-022-11865-7)
Supplement: Supplementary file 1 — Supplementary Information 1. [file 41598_2022_11865_MOESM1_ESM.pdf]

Supplementary Material

Diagnostic Accuracy of Keystroke dynamics as digital biomarkers for fine motor decline in neuropsychiatric disorders: a systematic review and meta-analysis

Hessa Al Falahi [1,*], Ahsan H. Khandoker [1,2], Nayeefa Chowdhury[1], Dimitrios Iakovakis[3], Sofia B. Dias[1,4], K. Ray. Chaudhuri[5,6], and Leontios Hadjileontiadis[1,2,3]

[1]Department of Biomedical Engineering, Khalifa University of Science and Technology, Abu Dhabi, UAE
[2]Healthcare Engineering Innovation Center, Khalifa University of Science and Technology, Abu Dhabi, UAE
[3]Department of Electrical and Computer Engineering, Aristotle University of Thessaloniki, Thessaloniki, Greece
[4]Faculdade de Motricidade Humana, University of Lisbon, 1495-751 Cruz Quebrada, Portugal
[5]Parkinson's Foundation Centre of Excellence, King's College Hospital NHS Foundation Trust, Denmark Hill, London SE5 9RS, UK
[6]Institute of Psychiatry, Psychology Neuroscience, Department of Basic and Clinical Neurosciences, King's College London, De Crespigny Park, London SE5 8AF, UK
[*]E-mail: hessa.alfalahi@ku.ac.ae, h.mjlad@gmail.com, P O Box 127788, Abu Dhabi, UAE, +971505252160

March 31, 2022

Contents

1 Systematic Search Strategy **2**

2 Titles of Excluded Full Text Articles **7**

3 Quality Assessment of Included Studies **7**
3.1 Domain 1: Patient Selection . . . 7
3.2 Domain 2: Index Test . . . 8
3.3 Domain 3: Reference Standard . . . 8
3.4 Domain 4: Flow and Timing . . . 9
3.5 Domain 5: Additional Items . . . 9

4 GRADE Tool **14**
4.1 Criteria for Quality of Evidence Assessment using the GRADE Tool . . . 14

5 Publication Bias **17**

6 Subgroup Analysis:*Forest Plots* **21**

7 Regression Results **30**

8 Key Points **31**

1 Systematic Search Strategy

Supplementary Table 1: Search Strategy of EBSCO

Num	Search Terms	Results
1	digital biomarkers AND neurology	626
2	digital phenotyping AND neurology	55
3	digital biomarkers AND psychiatry	407
4	digital phenotyping AND psychiatry	362
5	digital biomarkers AND Parkinson's Disease	233
6	digital phenotyping AND Parkinson's Disease	16
7	digital biomarkers AND cognitive impairment	259
8	digital phenotyping AND cognitive impairment	13
9	digital biomarkers AND (psychiatric disorders or mental illness or mental disorder)	265
10	digital phenotyping AND (psychiatric disorders or mental illness or mental disorder)	182
11	keystroke dynamics NOT security NOT authentication	852
12	keystroke dynamics OR typing behavior AND (parkinson's disease or parkinson disease or parkinsons disease or pd or parkinsons or parkinsonism)	18
13	keystroke dynamics OR typing behavior AND (mild cognitive impairment or mci or mild dementia or early stage dementia)	4
14	keystroke dynamics OR typing behavior AND (depression or depressive disorder or depressive symptoms or major depressive disorder)	12
15	keystroke dynamics OR typing behavior AND (bipolar disorder or bipolar i or bipolar ii or manic depression or bipolar affective disorder)	11

Supplementary Table 2: Search Strategy of MEDLINE

Num	Search Terms	Results
1	Digital biomarkers	550
2	Digital Phenotyping	330
3	Digital Biomarkers AND Neurology	92
4	Digital phenotyping AND Neurology	15
5	Digital Biomarkers AND Psychiatry	74
6	Digital phenotyping AND Psychiatry	151
7	Digital Biomarkers AND Parkinson's Disease	42
8	Digital Phenotyping AND Parkinson's Disease	4
9	Digital Biomarkers AND (cognitive impairment OR cognitive dysfunction OR cognitively impaired)	36
10	Digital Phenotyping AND (cognitive impairment OR cognitive dysfunction OR cognitively impaired)	7
11	Digital Biomarkers AND (psychiatric disorders OR mental disorder OR psychiatric disorder)	16
12	Digital Phenotyping AND (psychiatric disorders OR mental disorder OR psychiatric disorder)	44
13	Keystroke dynamics NOT Security NOT Authentication	26
14	(keystroke dynamics OR smartphone typing OR keyboard typing) AND (parkinson's disease OR pd OR parkinsonism)	8
15	(keystroke dynamics OR smartphone typing OR keyboard typing) AND (cognitive impairment OR cognitive dysfunction OR cognitively impaired)	1
16	(keystroke dynamics OR smartphone typing OR keyboard typing)AND (depression or depressive symptoms or major depressive disorder)	7
17	(keystroke dynamics OR smartphone typing OR keyboard typing) AND (bipolar i or bipolar ii or manic depression or bipolar affective disorder)	3

Supplementary Table 3: Search Strategy of IEEEXPLORE

Num	Search Terms	Results
1	Digital biomarkers	195
2	Digital Phenotyping	53
3	Digital Biomarkers AND Neurology	4
4	Digital phenotyping AND Neurology	2
5	Digital Biomarkers AND Psychiatry	2
6	Digital phenotyping AND Psychiatry	4
7	Digital Biomarkers AND Parkinson's Disease	7
8	Digital Phenotyping AND Parkinson's Disease	4
9	Digital Biomarkers AND (cognitive impairment OR cognitive dysfunction OR cognitively impaired)	356
10	Digital Phenotyping AND (cognitive impairment OR cognitive dysfunction OR cognitively impaired)	7
11	Keystroke dynamics NOT Security NOT Authentication	57
12	(keystroke dynamics OR smartphone typing OR keyboard typing) AND (parkinson's disease OR parkinson disease OR parkinsons disease OR pd OR parkinsons OR parkinsonism)	571
13	(keystroke dynamics OR smartphone typing OR keyboard typing) AND (cognitive impairment OR cognitive dysfunction OR cognitively impaired)	401
14	(keystroke dynamics OR smartphone typing OR keyboard typing)AND (depression or depressive disorder or depressive symptoms or major depressive disorder)	267
15	(keystroke dynamics OR smartphone typing OR keyboard typing) AND (bipolar disorder or bipolar i or bipolar ii or manic depression or bipolar affective disorder or bipolar depression)	301

Supplementary Table 4: Search Strategy of Web of Science

Num	Search Terms	Results
1	Digital Biomarkers AND Neurology	53
2	Digital phenotyping AND Neurology	11
3	Digital Biomarkers AND Psychiatry	175
4	Digital phenotyping AND Psychiatry	196
5	Digital Biomarkers AND Parkinson's Disease	66
6	Digital Phenotyping AND Parkinson's Disease	10
7	Digital Biomarkers AND (cognitive impairment OR cognitive dysfunction OR cognitively impaired)	113
8	Digital Phenotyping AND (cognitive impairment OR cognitive dysfunction OR cognitively impaired)	19
9	Digital Biomarkers AND (psychiatric disorders OR mental disorder OR mental illness OR psychiatric disorder)	126
10	Keystroke dynamics NOT Security NOT Authentication	231
11	(keystroke dynamics OR smartphone typing OR keyboard typing) AND (parkinson's disease OR parkinson disease OR parkinsons disease OR pd OR parkinsons OR parkinsonism)	6
12	(keystroke dynamics OR smartphone typing OR keyboard typing) AND (cognitive impairment OR cognitive dysfunction OR cognitively impaired)	1
13	(keystroke dynamics OR smartphone typing OR keyboard typing)AND (depression or depressive disorder or depressive symptoms or major depressive disorder)	7
14	(keystroke dynamics OR smartphone typing OR keyboard typing) AND (bipolar disorder or bipolar i or bipolar ii or manic depression or bipolar affective disorder or bipolar depression)	7

Supplementary Table 5: Search Strategy of PubMed

Num	Search Terms	Results
1	Digital Biomarkers AND Neurology	260
2	Digital phenotyping AND Neurology	141
3	Digital Biomarkers AND Psychiatry	181
4	Digital phenotyping AND Psychiatry	254
5	Digital Biomarkers AND Parkinson's Disease	82
6	Digital Phenotyping AND Parkinson's Disease	26
7	Digital Biomarkers AND (cognitive impairment OR cognitive dysfunction OR cognitively impaired)	61
8	Digital Phenotyping AND (cognitive impairment OR cognitive dysfunction OR cognitively impaired)	8
9	Digital Biomarkers AND (psychiatric disorders OR mental disorder OR mental illness OR psychiatric disorder)	165
10	digital phenotyping AND (psychiatric disorders or mental illness or mental disorder)	251
11	Keystroke dynamics NOT Security NOT Authentication	42
12	(keystroke dynamics OR smartphone typing OR keyboard typing) AND (parkinson's disease OR parkinson disease OR pd OR parkinsonism)	7
13	(keystroke dynamics OR smartphone typing OR keyboard typing) AND (cognitive impairment OR cognitive dysfunction OR cognitively impaired)	2
14	(keystroke dynamics OR smartphone typing OR keyboard typing)AND (depression or depressive disorder or major depressive disorder)	4
15	(keystroke dynamics OR smartphone typing OR keyboard typing) AND (bipolar i or bipolar ii or manic depression or bipolar affective disorder)	301

2 Titles of Excluded Full Text Articles

:

- Detecting cognitive impairment using keystroke and linguistic features of typed text: toward an adaptive method for continuous monitoring of cognitive status
- Detecting cognitive and physical stress through typing behavior
- Real-world keystroke dynamics are a potentially valid biomarker for clinical disability in multiple sclerosis
- Early-warning signals for disease activity in patients diagnosed with multiple sclerosis based on keystroke dynamics
- Monitoring the Motor Phenotype in Huntington's Disease by Analysis of Keyboard Typing During Real Life Computer Use

3 Quality Assessment of Included Studies

3.1 Domain 1: Patient Selection

Criteria	description
Q1A: Was a consecutive or random sample of patients enrolled?	
Q1B: Was a case-control design avoided?	Studies that included patients with uncertain diagnosis score as ”yes”. Studies that included healthy controls and patients with confirmed PD, MCI or mood disorders score as ”no”. If no relevant information provided score as ”unclear”.
Q1C: Did the study avoid inappropriate exclusions?	If the study excluded difficult to diagnose patients score as ”no”, if no information provided score as ”unclear”.
Q1D: Was the sample size appropriate?	If sample size calculations are performed score as ”yes”, and mention if it was adequate.
Q1E: Could the selection of patients have introduced bias?	If the patients were considered eligible for the study at early stage PD or MCI, then score as ”no”. Also, if the patients with clinically validated mood disorder, score as ”no”. On the other hand, if the patients self-reported their symptoms without clinical diagnosis, or if the study included patients with advanced disease stages, score as ”yes”.
Q1F: Is there concern that the included patients do not match the review question	

3.2 Domain 2: Index Test

Criteria	description
Q2A: Were the index test results interpreted without knowledge of the results of the reference standard	If the data collection and analysis was carried out without knowing the labels then score as ”yes”.
Q2B: If a threshold was used, was it pre-specified?	If the diagnosis based on the typing features was conducted with predefined threshold by the authors, score as ”yes”. If no relevant information provided, score as ”unclear”.
Q2C: Could the interpretation of the index test have introduced bias?	If the quantitative analysis introduced bias, due to the experimental setting, analysis models, study duration, medication impact, score as ”yes” and mention the reason.
Q2D: Is there concern that the interpretation or the conduct of the index test differ from the review question	

3.3 Domain 3: Reference Standard

Criteria	description
Q3A: Is the reference standard likely to correctly classify the target conditions?	If the diagnosis is confirmed via gold standard clinical scales, for PD, MCI, and mood disorders, then score as ”yes”. If the patients self-reported their symptoms without clinical validity score as ”no”.
Q3B: Were the reference standard interpreted without knowledge of the index test?	If the results of clinical standards were interpreted without knowledge of the typing behavior, score as ”yes”. If no relevant information reported, score as ”unclear”.
Q3C: Could the reference standard, its conduct or interpretation, have introduced bias?	This is a ”no” for all studies, except those using self-reports as a ground truth.
Q3D: Is there concern that the target conditions, as defined by the reference standard does not match the research question	

3.4 Domain 4: Flow and Timing

Criteria	description
Q4A: Was there an appropriate interval between the index tests and the reference standard?	If the interval between the conduct of the clinical reference standard and the acquisition of the index tests is no more than six months, score as ”yes”. If no relevant information of the time is reports, score as ”unclear”.
Q4B: Did all the patients receive a reference standard?	If all patients, whom typing data were collected, received a standard, clinical test, score as ”yes”. This item should be scored as ”unclear” if no relevant information is provided.
Q4C: Did patients receive the same reference standard?	If all patients were evaluated by the same clinical scale, this item is scored as ”yes”.
Q4D: Were all patients included in the analysis?	If some patients that were enrolled to begin with, but excluded from the analysis, this item should be scored as ”yes”. Discuss the exclusion reasons, and whether the explanation is sufficient to preclude bias.

3.5 Domain 5: Additional Items

Item	description
1: Were uninterpretable or intermediate test results reported?	If the authors reported uninterpretable test results, score as ”yes”, otherwise, this item is scored as ”unclear” if it is not possible to tell if there were uninterpretable results, and ”no” if the authors reported no uninterpretable results
2: Were the analyzed behavioral data, and their extracted features, available for comparison?	If the quantitative features, extracted from the biomarkers, were available for interpretation, score as ”yes”
3: Was the method, wether for statistical analysis or classification, consistent through the study?	If the method used in the study is consistent for all participants, score as ”yes”. if no information is sufficient to make a judgement, state as ”unclear”

Supplementary Table 6: Quality Assessment of the Included Studies

Study	*Patient Selection*						*Index Test*				*Reference Standard*				*Flow and Timing*				*Additional Items*		
	**Q1A**	**Q1B**	**Q1C**	**Q1D**	**Q1E**	**Q1F**	**Q2A**	**Q2B**	**Q2C**	**Q2D**	**Q3A**	**Q3B**	**Q3C**	**Q3D**	**Q4A**	**Q4B**	**Q4C**	**Q4D**	**1**	**2**	**3**
Giancardo et al.(2016)[1]	Y	N	U	Y	N	N	Y	U	N	N	Y	Y	N	N	Y	Y	Y	N	U	Y	Y
Arroyo-Gallego et al. (2017)[2]	Y	N	U	. U	N	N	N	U	U	N	Y	N	N	N	Y	Y	Y	N	U	Y	Y
Arroyo-Gallego et al. (2018)[3]	Y	N	U	. Y	N	Y	Y	U	N	Y	Y	U	N	Y	Y	Y	N	U	Y	Y	Y
Iakovakis et al. (2018)[4]	Y	N	U	. Y	N	N	Y	Y	U	N	Y	Y	N	N	Y	Y	Y	Y	N	Y	Y
Iakovakis et al. (2018)[5]	N	Y	Y	Y	Y	U	Y	U	N	N	N	Y	U	U	Y	Y	Y	N	N	Y	Y
Matarazzo et al. (2019)[6]	Y	N	Y	Y	N	N	Y	Y	U	N	Y	N	N	N	Y	Y	Y	N	U	Y	Y
Prince et al. (2018)[7]	Y	N	Y	Y	N	N	Y	Y	U	N	Y	U	U	N	Y	Y	Y	N	U	Y	Y
Lipsmeier et al. (2018)[8]	Y	N	Y	U	N	N	Y	U	U	N	Y	Y	U	N	Y	Y	Y	N	U	U	Y
Chen et al. (2019)[9]	Y	N	Y	U	U	N	Y	U	N	N	Y	Y	N	N	Y	Y	Y	Y	U	Y	Y
Stringer et al. (2018)[10]	Y	N	Y	Y	N	N	N	N	N	N	Y	Y	N	N	Y	Y	Y	Y	U	Y	Y
Ntracha et al. (2020)[11]	Y	N	Y	U	N	N	Y	U	U	N	Y	Y	N	N	Y	Y	Y	Y	N	Y	Y
Vizer et al. (2015)[12]	Y	N	Y	U	N	N	Y	U	N	N	Y	Y	N	N	Y	Y	Y	Y	U	Y	Y
Rabinowitz et al. (2014)[13]	Y	Y	Y	Y	N	N	Y	U	N	N	Y	Y	N	N	Y	Y	Y	Y	U	Y	Y
Waes et al. (2017)[14]	Y	N	Y	U	N	N	Y	U	N	N	Y	Y	N	N	Y	Y	Y	Y	U	Y	Y
Lee et al. (2016)[15]	Y	N	Y	U	N	N	Y	U	N	N	Y	Y	N	N	Y	Y	Y	Y	U	Y	Y
Arora et al. (2018)[16]	Y	N	Y	Y	N	N	Y	N	N	N	Y	Y	N	N	Y	Y	Y	Y	U	Y	Y
Memedi et al. (2013)[17]	Y	N	Y	Y	N	N	Y	U	N	N	U	Y	U	N	Y	Y	Y	Y	U	Y	Y
Zhan et al. (2016)[18]	Y	N	Y	Y	Y	N	Y	N	N	N	U	U	U	U	Y	Y	Y	Y	U	U	Y
Printy et al. (2014)[19]	Y	Y	U	U	U	N	U	Y	N	N	Y	Y	N	N	Y	Y	Y	Y	Y	U	U
Huang et al. (2018)[20]	Y	N	Y	U	U	N	Y	N	N	N	Y	Y	N	N	Y	Y	Y	Y	N	N	Y
Cao et al. (2017)[21]	Y	N	Y	U	U	N	Y	N	N	N	Y	Y	N	N	Y	Y	Y	Y	N	N	Y

Study	*Patient Selection*						*Index Test*				*Reference Standard*				*Flow and Timing*				*Additional Items*		
	**Q1A**	**Q1B**	**Q1C**	**Q1D**	**Q1E**	**Q1F**	**Q2A**	**Q2B**	**Q2C**	**Q2D**	**Q3A**	**Q3B**	**Q3C**	**Q3D**	**Q4A**	**Q4B**	**Q4C**	**Q4D**	**1**	**2**	**3**
Pham et al.(2018)[22]	Y	N	U	U	N	N	Y	U	N	N	Y	Y	N	N	Y	Y	Y	N	U	Y	Y
Pham et al.(2019)[23]	Y	N	U	U	N	N	Y	U	N	N	Y	Y	N	N	Y	Y	Y	N	U	Y	Y
Milne et al. (2018)[24]	Y	N	U	U	N	N	Y	U	N	N	Y	Y	N	N	Y	Y	Y	N	U	Y	Y
Papadopoulos et al. (2020)[25]	Y	N	Y	Y	Y	N	Y	N	Y	N	Y	Y	N	N	Y	Y	Y	Y	U	Y	Y
Mastoras et al. (2019)[26]	Y	N	Y	Y	Y	N	Y	N	Y	N	Y	Y	N	N	Y	Y	Y	Y	U	Y	Y
Iakovakis et al. (2019)[27]	Y	N	Y	Y	Y	N	Y	N	Y	N	Y	Y	N	N	Y	Y	Y	Y	U	Y	Y
Iakovakis et al. (2020)[28]	Y	N	Y	Y	Y	N	Y	N	Y	N	Y	Y	N	N	Y	Y	Y	Y	U	Y	Y
Wissel et al. (2017)[29]	Y	N	U	U	N	N	Y	U	N	N	Y	Y	N	N	Y	Y	Y	N	U	Y	Y
Adams et al. (2017)[30]	Y	N	U	U	N	N	Y	U	N	N	Y	Y	N	N	Y	Y	Y	N	U	N	Y
Zulueta et al. (2018)[31]	Y	N	Y	U	U	N	Y	N	N	N	Y	Y	N	N	Y	Y	Y	Y	N	N	Y
Stange et al. (2018)[32]	Y	N	U	U	U	N	Y	N	N	N	Y	Y	N	N	Y	Y	Y	Y	N	N	Y
Vesel et al. (2020)[33]	Y	N	Y	U	U	N	Y	N	N	N	Y	Y	N	N	Y	Y	Y	Y	N	N	Y
Giancardo et al. (2015)[34]	Y	Y	U	U	N	N	Y	U	N	N	Y	Y	N	N	Y	Y	Y	N	U	Y	Y
Surangsrirat et al. (2022)[35]	Y	N	Y	Y	Y	N	Y	N	Y	N	Y	Y	N	N	Y	Y	Y	Y	U	Y	Y
Wang et al. (2021)[36]	Y	N	Y	Y	Y	N	Y	N	Y	N	Y	Y	N	N	Y	Y	Y	Y	U	Y	Y
Goni et al. (2021)[37]	Y	N	Y	Y	Y	N	Y	N	Y	N	Y	Y	N	N	Y	Y	Y	Y	U	Y	Y
Zulueta et al. (2022)[38]	Y	N	Y	U	U	N	Y	N	N	N	Y	Y	N	N	Y	Y	Y	Y	N	N	Y
Ross et al. (2021)[39]	Y	N	Y	U	U	N	Y	N	N	N	Y	Y	N	N	Y	Y	Y	Y	N	N	Y

Y: Yes, N: No, U:Unknown

Supplementary Table 7: Contingency Data of the Meta Analysis Studies

**Study**	**Diseased**	**Healthy**	**Sensitivity**	**Specificity**	**AUC**	**Accuracy**	**TP**	**TN**	**FP**	**FN**	Additional Notes
Chen et al. (2019)[9]	31	82	NR	NR	0.80	NR	NR	NR	NR	NR	Mild Cognitive Impairment (MCI)
Chen et al. (2019)[9]	31	82	NR	NR	0.77	NR	NR	NR	NR	NR	MCI, without demographics consideration
Chen et al. (2019)[9]	7	82	NR	NR	0.92	NR	NR	NR	NR	NR	Alzheimer's Disease (AD) and Dementia
Stringer et al. (2018)[10]	20	24	0.84	0.67	0.80	0.73	16	16	8	4	pauses per minute
Stringer et al. (2018)[10]	20	24	0.89	0.78	0.91	0.80	17	18	6	3	keystrokes per minute
Stringer et al. (2018)[10]	20	24	0.89	0.99	0.98	0.95	18	24	0	2	combined computer use behavior
Ntracha et al. (2020)[11]	10	7	0.71	0.80	0.76	0.76	7	5	3	3	linguistic features (using LR)
Ntracha et al. (2020)[11]	11	12	0.92	0.64	0.78	0.77	10	7	5	1	keystroke models (kNN)
Ntracha et al. (2020)[11]	10	5	0.60	0.90	0.75	0.80	6	4	1	4	fused model (kNN)
Vizer et al. (2015)[12]	17	21	0.71	0.83	0.81	0.83	12	17	4	5	No special description (main study method)
Lee et al. (2016)[15]	57	87	NR	NR	0.91	NR	NR	NR	NR	NR	LR model using the total distance as a predictive value
Arora et al. (2018)[16]	334	84	0.85	0.88	NR	0.86	283	73	11	51	Parkinson's Disease
Arora et al. (2018)[16]	104	84	0.91	0.90	NR	0.86	94	75	9	10	Idiopathic REM Sleep Behavior
Memedi et al. (2013)[17]	95	10	NR.	NR	0.86	NR.	NR	. NR	NR	NR	-
Iakovakis et al. (2018)[4]	18	15	0.82	0.81	0.92	0.91	14	12	3	4	-
Giancardo et al. (2016)[1]	42	43	0.71	0.84	0.81	0.78	29	36	7	13	-
Iakovakis et al.(2018)[5]	13	35	0.77	0.8	0.84	0.79	10	28	7	3	rigidity index for 52 weeks
Iakovakis et al.(2018)[5]	13	35	0.92	0.63	0.80	0.69	12	32	22	1	Using the bradykinesia index for 52 weeks
Iakovakis et al.(2018)[5]	13	35	0.82	0.65	0.80	0.67	10	22	13	3	Using the rigidity index for 1 week
Iakovakis et al.(2018)[5]	13	35	0.86	0.60	0.78	0.71	11	21	14	2	Using the bradykinesia index for 1 week
Goni et al. (2021)[37]	970	1630	NR	NR	0.74	NR	NR	NR	NR	NR	

Study	Diseased	Healthy	Sensitivity	Specificity	AUC	Accuracy	TP	TN	FP	FN	Additional Notes
Pham et al. (2018)[22]	42	43	0.98	0.99	0.98	0.92	41	43	0	1	-
Pham et al. (2019)[23]	42	43	0.95	0.63	NR	0.79	40	27	16	2	-
Arroyo Gallego et al. (2018)[3]	25	27	NR	NR	0.76	0.75	NR	NR	NR	NR	-
Arroyo Gallego et al. (2017)[2]	21	23	0.73	0.72	0.83	0.70	15	16	7	6	raw flight time (FT)
Arroyo Gallego et al. (2017)[2]	21	23	0.81	0.81	0.88	0.80	17	17	6	4	Univariate model
Arroyo Gallego et al. (2017)[2]	21	23	0.73	0.84	0.91	0.82	15	19	4	6	Multivariate model
Milne et al. (2018)[24]	42	43	0.64	0.81	0.85		16	35	8	26	-
Printy et al. (2014)[19]	18	NR	NR	NR	0.91	0.95	NR	NR	NR	NR	-
Adams et al. (2017)[30]	12	38	0.92	0.95	0.79	0.94	11	36	2	2	-
Oroojeni et al. (2019)[40]	42	43	0.74	0.91	0.85	0.88	31	39	4	11	-
Giancardo[1]	42	43	0.71	0.84	0.81	0.78	29	36	7	13	-
Iakovakis et al. (2019)[27]	18	15	0.79	0.79	0.89	0.89	14	12	3	4	data captured in-the-clinic
Iakovakis et al. (2019)[27]	27	84	0.74	0.78	0.79	0.79	20	65	19	7	data captured in the wild
Iakovakis et al. (2020)[28]	22	17	0.90	0.83	0.89	20	14	3	2	TS1	
Iakovakis et al. (2020)[28]	9	17	0.90	0.83	0.89	8	14	3	1	TS2	
Lee et al. (2016)[15]	57	87	NR	NR	0.88	NR	NR	NR	NR	NR	LR model using the dwelling time as a predictive value
Giancardo et al. (2015)[34]	14	14	NR	NR	0.91	NR	NR	NR	NR	NR	-
Mastoras et al. (2019)[26]	11	14	0.82	0.86	0.85	0.84	9	12	2	2	Threshold of 5 (PHQ-9)
Mastoras et al. (2019)[26]	17	8	0.75	0.82	0.81	0.72	13	6	2	4	Threshold of 10 (PHQ-9)
Cao et al. (2017)[21]	12	8	NR	NR	NR	0.90	NR	NR	NR	NR	-
Papadopoulos et al. (2020)[25]	14	8	0.93	0.93	0.89	0.91	13	7	7	1	SData (clinically validated)
Papadopoulos et al. (2020)[25]	26	131	0.92	0.69	NR	0.86	24	90	41	2	GData (self reported data)

NR: Not Reported, TS1: Testing scenario 1, TS2: Testing scenario 2, PHQ-9: Patient Health Questionnaire - 9, LR: Logistic Regression, SData: Small dataset, Gdata: Large dataset.

4 GRADE Tool

4.1 Criteria for Quality of Evidence Assessment using the GRADE Tool

**Criteria**	**description**
Study Design	Lower score if cohort study
Risk of Bias	score based on the QUADAS-2 tool.
Indirectness of outcome	Lower score if the study collected data in-the-wild and/or labeled data by self-reports.
Inconsistency/ imprecision of results	Lower score if the reported accuracy measures are inconsistent.
Publication Bias	This is deemed low risk for all included studies, given we maintained funnel plots symmetry upon study inclusion.

Supplementary Table 8: Evidence Profile (EP) as per the GRADE Tool for Included Studies

**Study**	**Study Design**	**Risk of Bias**	**Indirectness**	**Imprecision**	**Publication Bias**	**Quality of Evidence**
Giancardo et al.(2016)[1]	Case-Control Study	Low	None	None	None	High ⊕ ⊕ ⊕ ⊕
Arroyo-Gallego et al. (2017)[2]	Case-Control Study	Low	None	None	None	High ⊕ ⊕ ⊕ ⊕
Arroyo-Gallego et al. (2018)[3]	Case-Control Study	Low	Moderate risk (a)	None	None	Moderate ⊕ ⊕ ◯ ◯
Iakovakis et al. (2018)[4]	Case-Control Study	Low	None	None	None	High ⊕ ⊕ ⊕ ⊕
Iakovakis et al. (2018)[5]	Case-Control Study	Low	None	None	None	Moderate ⊕ ⊕ ◯ ◯
Matarazzo et al. (2019)[6]	Cohort Study	Moderate	None	None	None	Moderate ⊕ ⊕ ◯ ◯
Prince et al. (2018)[7]	Case-Control Study	Low	None	None	None	High ⊕ ⊕ ⊕ ⊕
Lipsmeier et al. (2018)[8]	Cohort Study, phase 1 clinical Trial	Low	None	None	None	High ⊕ ⊕ ⊕ ⊕
Chen et al. (2019)[9]	Case-Control Study	Low	None	None	None	Moderate ⊕ ⊕ ◯ ◯
Stringer et al. (2018)[10]	Case-Control Study	Low	None	None	None	High ⊕ ⊕ ⊕ ⊕
Ntracha et al. (2020)[11]	Case-Control Study	Low	None	None	None	High ⊕ ⊕ ⊕ ⊕
Vizer et al. (2015)[12]	Case-Control Study	Low	None	None	None	Moderate ⊕ ⊕ ◯ ◯
Rabinowitz et al. (2014)[13]	Case-Control Study	Low	None	None	None	Moderate ⊕ ⊕ ◯ ◯
Waes et al. (2017)[14]	Case-Control Study	Low	None	None	None	Moderate ⊕ ⊕ ◯ ◯
Lee et al. (2016)[15]	Case-Control Study	Low	None	None	None	Moderate ⊕ ⊕ ◯ ◯
Arora et al. (2018)[16]	Case-Control Study	Low	None	None	None	High ⊕ ⊕ ⊕ ⊕
Memedi et al. (2013)[17]	Case-Control Study	Low	None	None	None	Moderate ⊕ ⊕ ◯ ◯
Zhan et al. (2016)[18]	Case-Control Study	Low	None	None	None	High ⊕ ⊕ ⊕ ⊕
Printy et al. (2014)[19]	Cohort study	Unclear	None	None	None	Low ⊕ ◯ ◯ ◯
Huang et al. (2018)[20]	Case-Control Study	Low	None	None	None	High ⊕ ⊕ ⊕ ⊕
Cao et al. (2017)[21]	Case-Control Study	Low	None	None	None	High ⊕ ⊕ ⊕ ⊕

a: data collected in-the-wild

Study	Study Design	Risk of Bias	Indirectness	Imprecision	Publication Bias	Quality of Evidence
Pham et al.(2018)[22]	Case-Control Study	Low	None	None	None	High ⊕ ⊕ ⊕ ⊕
Pham et al.(2019)[23]	Case-Control Study	Low	None	None	None	High ⊕ ⊕ ⊕ ⊕
Milne et al. (2018)[24]	Case-Control Study	Unclear	None	None	None	Moderate ⊕ ⊕ ◯ ◯
Papadopoulos et al. (2020)[25]	Case-Control Study	Low	None	None	None	High ⊕ ⊕ ⊕ ⊕
Mastoras et al. (2019)[26]	Case-Control Study	Low	None	None	None	High ⊕ ⊕ ⊕ ⊕
Iakovakis et al. (2019)[27]	Case-Control Study	Unclear	None	None	None	Moderate ⊕ ⊕ ◯ ◯
Iakovakis et al. (2020)[28]	Case-Control Study	Low	None	None	None	High ⊕ ⊕ ⊕ ⊕
Wissel et al. (2017)[29]	Case-Control Study	Unclear	High risk of indirectness	None	None	Low ⊕ ◯ ◯ ◯
Adams et al. (2017)[30]	Case-Control Study	Low	Unclear	None	None	Moderate ⊕ ⊕ ◯ ◯
Zulueta et al. (2018)[31]	Case-Control Study	Low	None	None	None	Moderate ⊕ ⊕ ◯ ◯
Stange et al. (2018)[32]	Case-Control Study	Unclear	None	None	None	Low ⊕ ◯ ◯ ◯
Vesel et al. (2020)[33]	Case-Control Study	Low	None	None	None	High ⊕ ⊕ ⊕ ⊕
Giancardo et al. (2015)[34]	Cohort Study	Low	None	None	None	Moderate ⊕ ⊕ ◯ ◯
Surangsrirat et al. (2022)[35]	Cohort Study	Low	None	None	None	High ⊕ ⊕ ⊕ ⊕
Wang et al. (2021)[36]	Case-Control Study	Low	None	None	None	Moderate ⊕ ⊕ ◯ ◯
Goni et al. (2021)[37]	Case-Control Study	Low	None	None	None	High ⊕ ⊕ ⊕ ⊕
Zulueta et al. (2022)[38]	Case-Control Study	Low	None	None	None	High ⊕ ⊕ ⊕ ⊕
Ross et al. (2021)[39]	Case-Control Study (d)	Low	None	None	None	Moderate ⊕ ⊕ ◯ ◯

5 Publication Bias

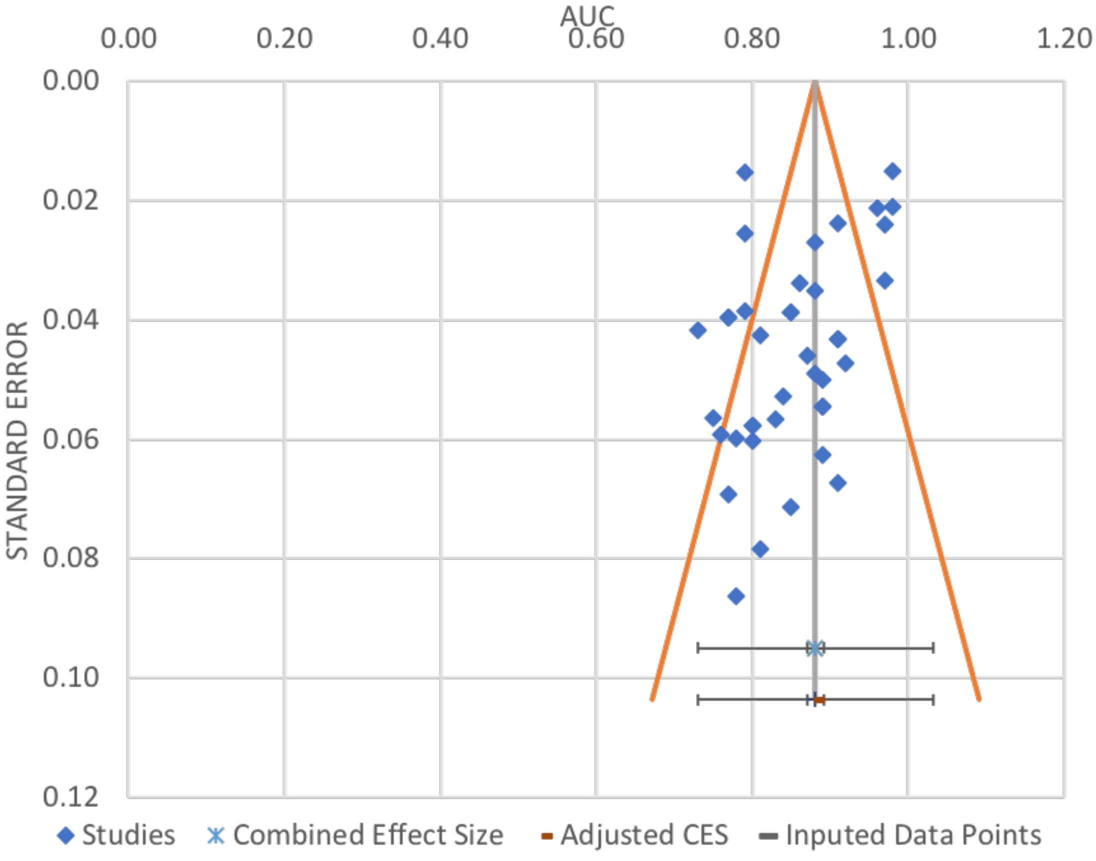

Figure S 1: Funnel plot of Pooled AUC.

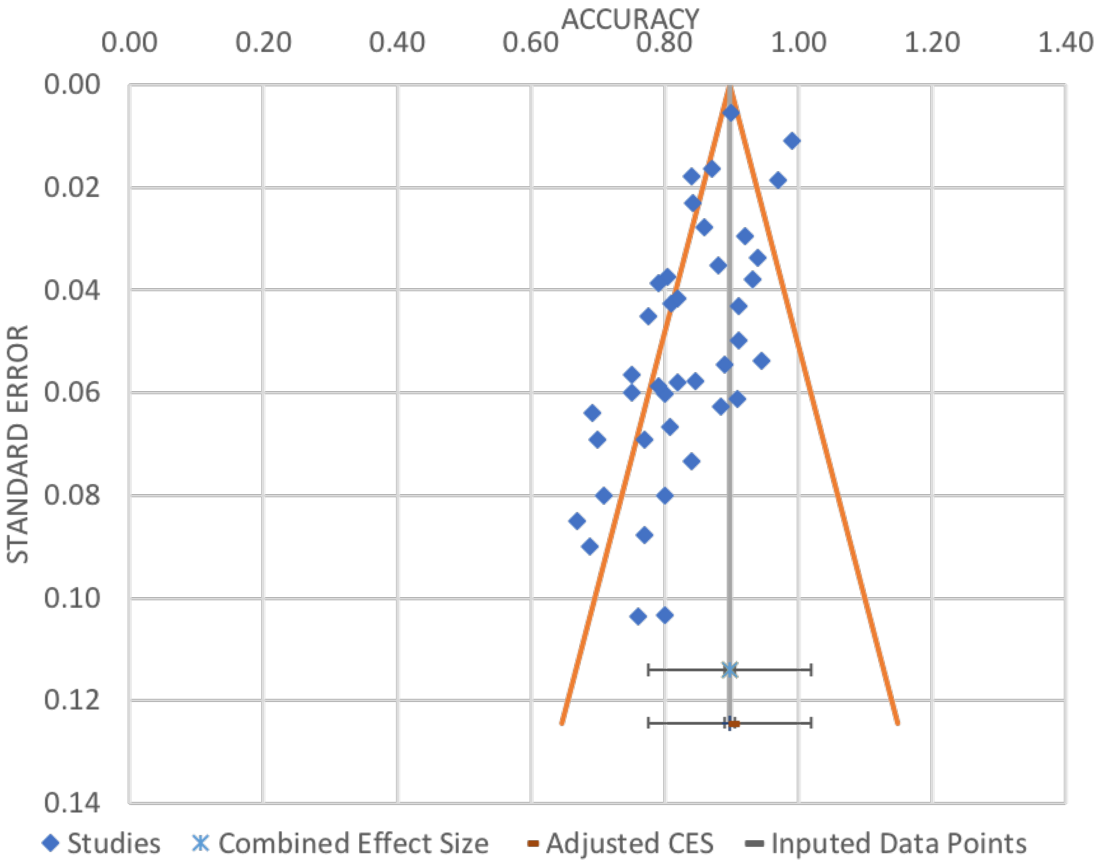

Figure S 2: Funnel plot of Pooled Accuracy.

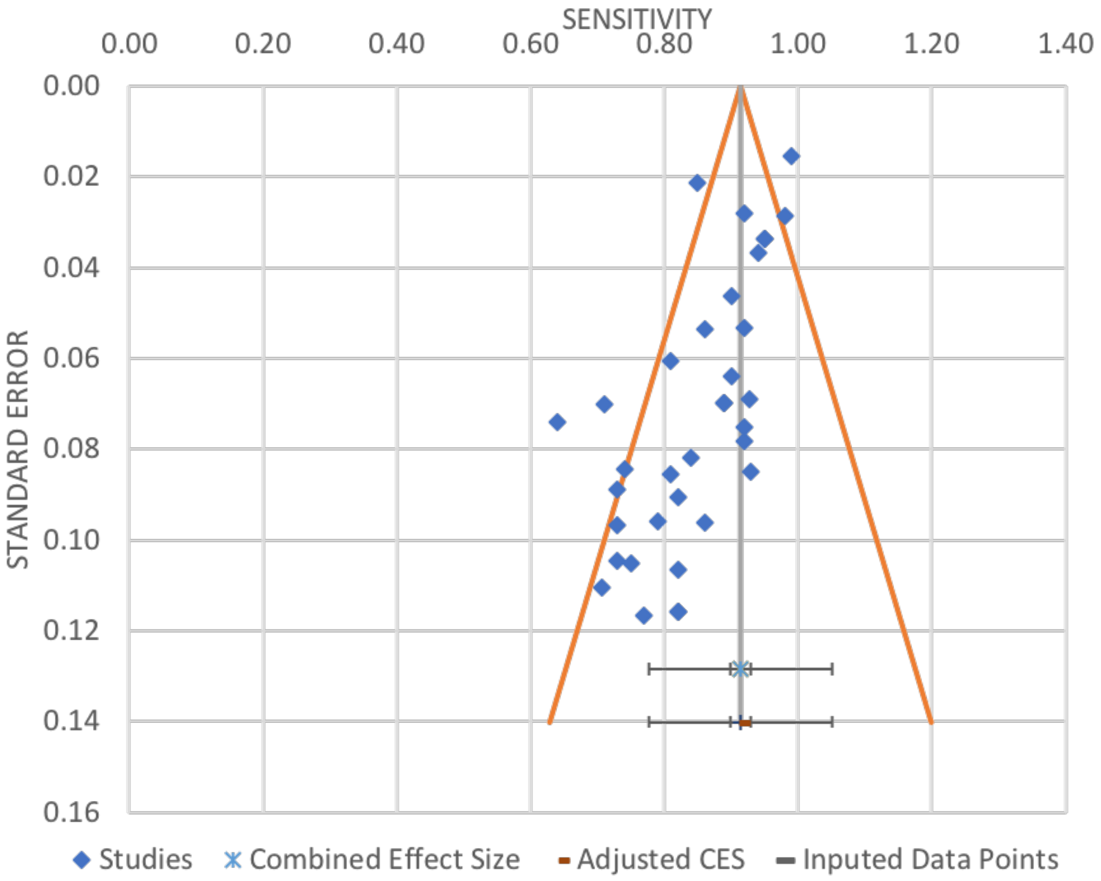

Figure S 3: Funnel plot of Pooled Sensitivity.

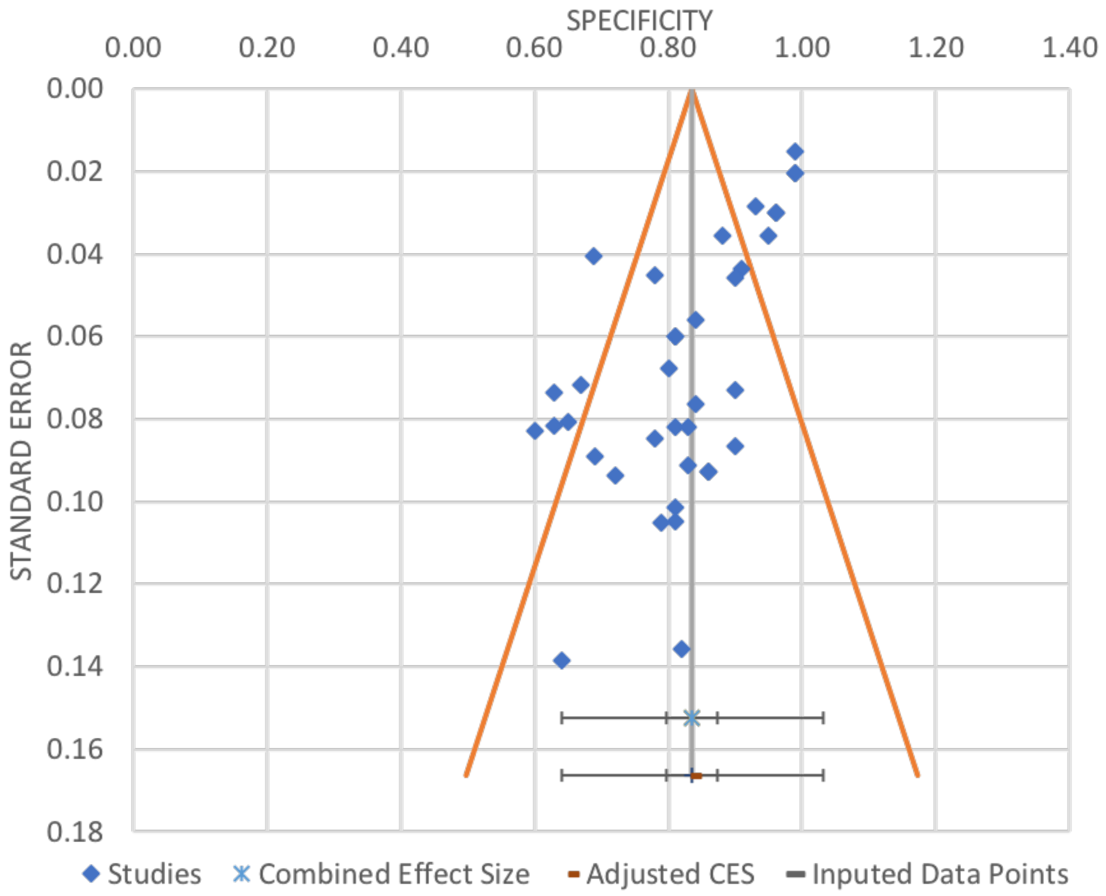

Figure S 4: Funnel plot of Pooled Specificity.

6 Subgroup Analysis: *Forest Plots*

Study name / Subgroup name	AUC	CI LL	CI UL	Weight
Stringer et al. (2017)	0.98	0.96	1.00	0.062
Iakovakis et al. (2018)	0.92	0.82	1.00	0.04
Pham et al.(2018)	0.98	0.95	1.00	0.06
Vizer et al. (2015)	0.81	0.67	0.94	0.03
Arroyo-Gallego et al. (2017)	0.83	0.72	0.94	0.04
Arroyo-Gallego et al. (2017)	0.91	0.82	1.00	0.05
Arroyo-Gallego et al. (2017)	0.88	0.78	0.98	0.04
Iakovakis et al. (2019)	0.89	0.78	1.00	0.04
Giancardo et al. (2016)	0.81	0.73	0.89	0.05
Giancardo et al. (2016)	0.79	0.76	0.82	0.06
Milne et al. (2018)	0.85	0.77	0.93	0.05
oroojeni et al. (2019)	0.85	0.77	0.93	0.05
Adams 2017	0.97	0.92	1.00	0.06
oroojeni et al. (2019)	0.88	0.81	0.95	0.05
Iakovakis 2019	0.87	0.75	0.99	0.04
Iakovakis 2019	0.88	0.76	1.00	0.04
Stringer 2017	0.80	0.68	0.92	0.04
Stringer 2017	0.91	0.82	1.00	0.05
Lee et al. (2016)	0.92	0.88	0.96	0.06
Lee et al. (2016)	0.88	0.83	0.93	0.06
Printy et al. (2014)	0.91	0.83	0.99	0.05
**In the clinic**	**0.89**	**0.86**	**0.91**	**0.50**
Chen et al. (2019)	0.77	0.69	0.85	0.06
Ntracha et al. (2020) - Multimodal	0.75	0.51	0.99	0.02
Papadopoulos et al. (2020)	0.89	0.80	0.98	0.05
Mastoras 2019 - Univariate	0.85	0.70	1.00	0.04
Arroyo-Gallego et al. (2018)	0.76	0.64	0.88	0.04
Iakovakis et al. (2020) (TS1)	0.89	0.79	0.99	0.05
Iakovakis et al. (2020) (TS2)	0.97	0.93	1.00	0.07
Iakovakis et al. (2020) (TS3)	0.79	0.74	0.84	0.06
Iakovakis et al. (2018) (Rigidity)	0.84	0.73	0.95	0.05
Iakovakis et al. 2018 (Bradykinesia)	0.80	0.68	0.92	0.04
Ntracha et al. (2020)	0.79	0.71	0.87	0.06
Ntracha et al. (2020)	0.78	0.60	0.96	0.03
Mastoras et al. (2019) - Multivariate	0.89	0.76	1.00	0.04
Chen et al. (2019)	0.80	0.73	0.87	0.06
Chen et al. (2019)	0.73	0.65	0.81	0.05
Iakovakis 2019	0.77	0.69	0.85	0.05
Iakovakis 2019	0.73	0.65	0.81	0.05
Mastoras 2019 - Univariate (HT)	0.81	0.65	0.97	0.03
Memedi et al. (2013)	0.86	0.79	0.93	0.06
Iakovakis et al. 2018 (Rigidity)	0.80	0.68	0.92	0.04
Iakovakis et al. 2018 (Bradykinesia)	0.78	0.66	0.90	0.04
**In the wild**	**0.82**	**0.79**	**0.84**	**0.50**
Combined effect size	0.85	0.78	0.92	

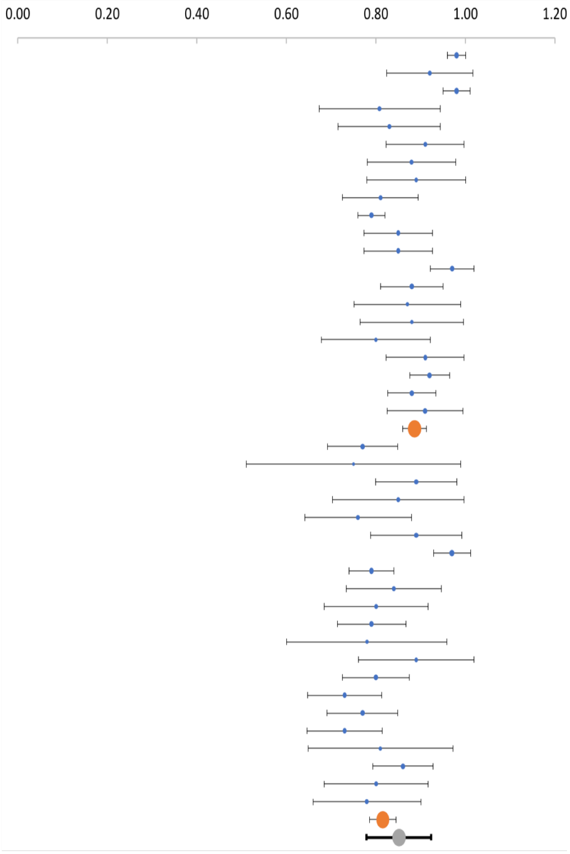

Figure S 5: Subgroup analysis results for AUC of data collected in-the-clinic vs. data collected in-the-wild .

Study name / Subgroup name	Accuracy	CI LL	CI UL	Weight
Stringer et al. (2017)	0.93	0.87	0.99	0.075
Iakovakis et al. (2018)	0.91	0.81	1.00	0.05
Vizer et al. (2015)	0.81	0.67	0.94	0.04
Arroyo-Gallego et al. (2017) - FT (raw)	0.70	0.56	0.84	0.03
Arroyo-Gallego et al. (2017) - Univariate Model	0.80	0.68	0.92	0.04
Arroyo-Gallego et al. (2017) - Multivariate Model	0.82	0.70	0.94	0.04
Iakovakis et al. (2019)	0.89	0.78	1.00	0.05
Giancardo et al. (2016)	0.78	0.69	0.87	0.06
Adams et al. (2017)	0.94	0.87	1.00	0.07
Arora et al. (2018)	0.84	0.80	0.88	0.09
Arora et al. (2018)	0.90	0.86	0.94	0.09
Oroojeni et al. (2019)	0.88	0.81	0.95	0.07
Pham 2018 - GLCM	0.92	0.86	0.98	0.08
Stringer et al. (2018)	0.91	0.82	1.00	0.06
Stringer et al. (2018)	0.72	0.58	0.86	0.03
Printy et al. (2014)	0.95	0.88	1.00	0.07
Giancardo et al. (2016)	0.76	0.61	0.91	0.03
Iakovakis et al. (2019)	0.79	0.65	0.93	0.03
**In the clinic**	**0.87**	**0.83**	**0.90**	**0.51**
Chen et al. (2019)	0.80	0.73	0.88	0.08
Papadopoulos et al. (2020)	0.91	0.83	0.99	0.08
Mastoras et al. (2019)	0.80	0.63	0.97	0.04
Arroyo-Gallego et al. (2018)	0.69	0.56	0.82	0.05
Arroyo-Gallego et al. (2018)	0.75	0.63	0.87	0.06
Iakovakis et al. (2019)	0.79	0.71	0.87	0.08
Cao et al. (2017)	0.90	0.89	0.91	0.11
Papadopoulos 2020	0.86	0.81	0.91	0.09
Iakovakis et al. (2020) TS1	0.85	0.73	0.96	0.06
Iakovakis et al. (2020) TS2	0.88	0.76	1.00	0.06
Mastoras et al. (2019)	0.84	0.69	0.99	0.05
Ntracha et al. (2020)	0.76	0.54	0.98	0.03
Ntracha et al. (2020)	0.77	0.59	0.95	0.04
Iakovakis et al. (2018) R	0.79	0.67	0.91	0.06
Iakovakis et al. (2018) B	0.69	0.51	0.87	0.04
Iakovakis et al. (2018) R	0.67	0.50	0.84	0.04
Iakovakis et al. (2018) B	0.71	0.55	0.87	0.04
**In the wild**	**0.81**	**0.77**	**0.85**	**0.49**
Combined effect size	0.84	0.78	0.89	

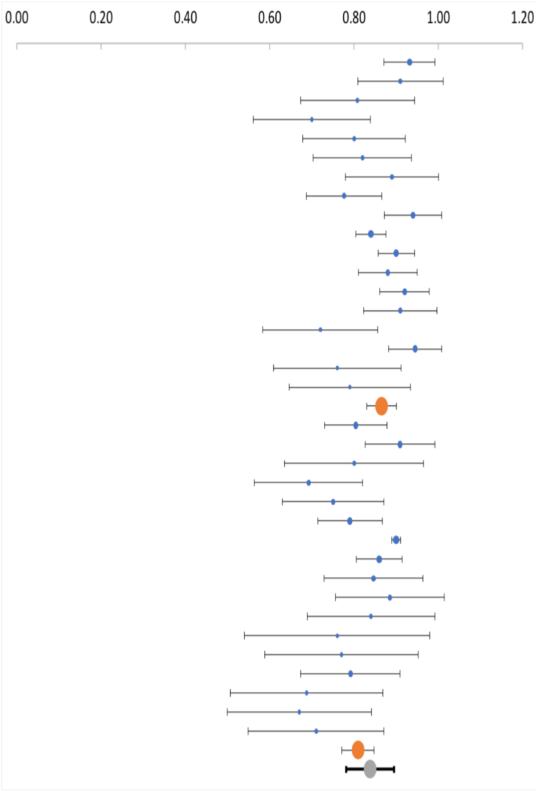

Figure S 6: Subgroup analysis results for Accuracy of data collected in-the-clinic vs. data collected in-the-wild .

Study name / Subgroup name	Sensitivity	CI LL	CI UL	Weight
Giancardo et al. (2016)	0.71	0.57	0.85	0.045
Alloyo et al. (2017) - Univariate Model	0.81	0.63	0.99	0.04
Alloyo et al. (2017) - Multivariate Model	0.73	0.53	0.93	0.03
Alloyo et al. (2017) - FT (raw)	0.73	0.51	0.95	0.03
Iakovakis et al. (2018)	0.82	0.63	1.00	0.03
Vizer et al. (201)	0.71	0.47	0.94	0.03
Stringer et al. (2018)	0.89	0.81	0.97	0.06
Milne et al.(2018) - Standard deviation	0.64	0.49	0.79	0.04
Iakovakis et al. (2019)	0.79	0.59	0.99	0.03
Pahm et al. (2018)	0.98	0.94	1.00	0.07
Pahm et al. (2018)	0.99	0.95	1.00	0.07
Alloyo et al. (2018)	0.73	0.55	0.91	0.04
Milne et al.(2018) - FRESH	0.86	0.75	0.97	0.05
Adams (2017)	0.92	0.83	1.00	0.06
Arora et al. (2018)	0.85	0.81	0.89	0.07
Arora et al. (2018)	0.92	0.86	0.98	0.07
Pham et al. (2018)	0.99	0.96	1.00	0.08
Stringer et al. (2018)	0.84	0.74	0.94	0.06
Iakovakis et al. (2019)	0.75	0.56	0.94	0.03
Iakovakis et al. (2019)	0.78	0.61	0.95	0.04
**In the Clinic**	**0.85**	**0.80**	**0.90**	**0.53**
Iakovakis et al. (2018) - Rigidity	0.77	0.55	0.99	0.04
Iakovakis et al. (2018) - Bradykinesia	0.92	0.83	1.00	0.11
Papadopoulos et al. (2020) - Sdata	0.93	0.86	0.99	0.13
Iakovakis et al. (2020) - TS1	0.90	0.80	1.00	0.09
Iakovakis et al. (2020) - TS2	0.93	0.86	1.00	0.13
Iakovakis et al. (2020) - TS3	0.76	0.63	0.89	0.07
Ntratcha et al. (2020)	0.92	0.81	1.00	0.09
Ntratcha et al. (2020)	0.60	0.27	0.93	0.02
Iakovakis et al. (2019)	0.74	0.57	0.91	0.05
Mastroras et al. (2019) - Univariate	0.82	0.64	1.00	0.06
Mastroras et al. (2019) - Univariate	0.75	0.56	0.94	0.05
Iakovakis et al. (2018) - Rigidity	0.82	0.65	0.99	0.06
Iakovakis et al. (2018) - Bradykinesia	0.86	0.71	1.00	0.07
Iakovakis et al. (2019)	0.65	0.46	0.84	0.05
**In the Wild**	**0.85**	**0.80**	**0.90**	**0.47**
Combined effect size	0.85	0.85	0.85	

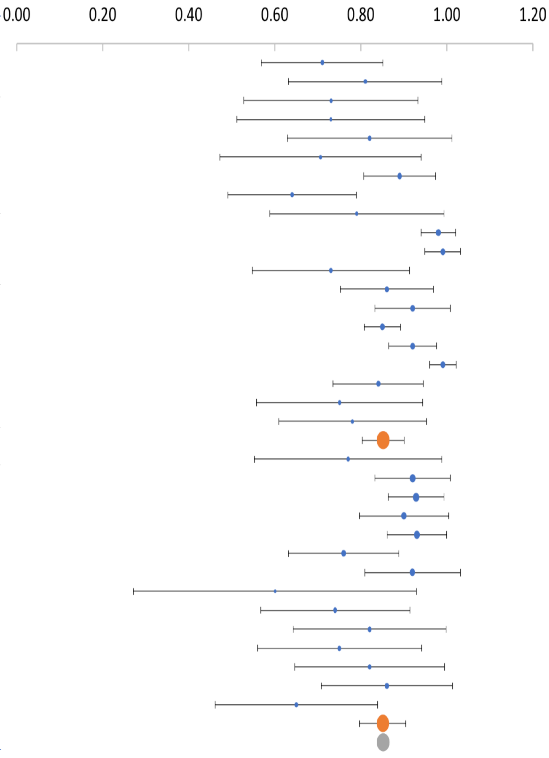

Figure S 7: Subgroup analysis results for Sensitivity of data collected in-the-clinic vs. data collected in-the-wild .

Study name / Subgroup name	Specificity	CI LL	CI UL	Weight
Giancardo et al. (2016)	0.84	0.73	0.95	0.053
Alloyo et al. (2017) - Univariate Model	0.81	0.64	0.98	0.04
Alloyo et al. (2017) - Multivariate Model	0.84	0.68	1.00	0.04
Alloyo et al. (2017) - FT	0.72	0.53	0.91	0.03
Vizer et al. (2015)	0.83	0.66	1.00	0.04
Stringer et al. (2018)	0.99	0.97	1.00	0.08
oroojeni et al. (2019)	0.84	0.73	0.95	0.05
Ntratcha et al. (2020)	0.90	0.79	1.00	0.06
Milne et al. (2018) - standard deviation	0.81	0.69	0.93	0.05
Iakovakis et al. (2019)	0.78	0.69	0.87	0.06
Alloyo et al. (2018)	0.69	0.51	0.87	0.03
Pahm et al. (2018)	0.96	0.92	1.00	0.08
Adams 2017	0.95	0.89	1.00	0.07
Arora et al. (2018)	0.88	0.81	0.95	0.07
Arora et al. (2018)	0.90	0.83	0.97	0.07
Stringer et al. (2018)	0.99	0.95	1.00	0.08
Stringer et al. (2018)	0.78	0.61	0.95	0.04
Iakovakis et al. (2019)	0.78	0.69	0.87	0.06
**In the Clinic**	**0.87**	**0.83**	**0.91**	**0.53**
Iakovakis et al. (2018)	0.81	0.64	0.98	0.05
Iakovakis et al. (2018) - Rigidity	0.80	0.66	0.94	0.06
Iakovakis et al. (2018) - Bradykinesia	0.63	0.46	0.80	0.05
Papadopoulos et al. (2020) - Sdata	0.93	0.87	0.99	0.08
Papadopoulos et al. (2020) - Gdata	0.69	0.61	0.77	0.08
Iakovakis et al. (2020) - TS1	0.83	0.66	1.00	0.05
Iakovakis et al. (2020) - TS2	0.90	0.79	1.00	0.07
Iakovakis et al. (2020) - TS3	0.71	0.64	0.78	0.08
Ntratcha et al. (2020)	0.64	0.34	0.94	0.03
Iakovakis et al. (2019)	0.79	0.62	0.96	0.05
Pahm et al. (2018)	0.90	0.81	0.99	0.07
Mastroras et al. (2019)	0.86	0.73	0.99	0.07
Mastroras et al. (2019)	0.82	0.65	0.99	0.06
Iakovakis et al. (2018) - Rigidity	0.65	0.49	0.81	0.05
Iakovakis et al. (2018) - Bradykinesia	0.60	0.43	0.77	0.05
Iakovakis et al.(2019)	0.92	0.86	0.98	0.08
**In the Wild**	**0.79**	**0.73**	**0.85**	**0.47**
Combined effect size	0.83	0.76	0.91	

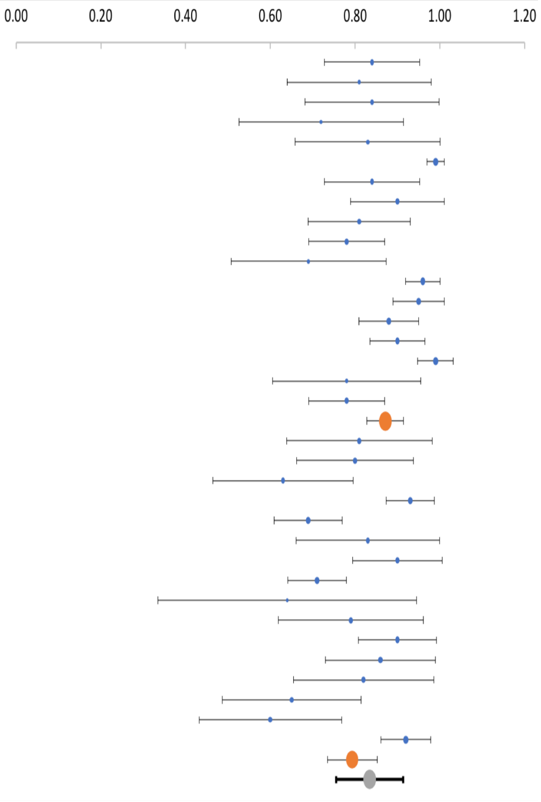

Figure S 8: Subgroup analysis results for Specificity of data collected in-the-clinic vs. data collected in-the-wild .

Study name / Subgroup name	AUC	CI LL	CI UL	Weight
Chen et al. (2019)	0.77	0.69	0.85	0.035
Stringer et al. (2017)	0.98	0.96	1.00	0.04
Ntracha et al. (2020) - Multiimodal	0.75	0.51	0.99	0.01
Iakovakis et al. (2018)	0.92	0.82	1.00	0.03
Pham et al. (2018)	0.98	0.95	1.00	0.04
Papadopoulos et al. (2020)	0.89	0.80	0.98	0.03
Vizer et al. (2015)	0.81	0.67	0.94	0.03
Arroyo-Gallego et al. (2018)	0.76	0.64	0.88	0.03
Arroyo-Gallego et al. (2017)	0.83	0.72	0.94	0.03
Giancardo et al. (2015)	0.91	0.82	1.00	0.03
Arroyo-Gallego et al. (2017)	0.88	0.78	0.98	0.03
Arroyo-Gallego et al. (2019) (All typers)	0.75	0.64	0.86	0.03
Arroyo-Gallego et al. (2019) (Consistent typers)	0.77	0.63	0.91	0.02
Iakovakis et al. (2019)	0.89	0.78	1.00	0.03
Giancardo et al. (2016)	)0.81	0.73	0.89	0.03
Giancardo et al. (2016)	0.79	0.76	0.82	0.04
Iakovakis et al. 2020 (TS1)	0.89	0.79	0.99	0.03
Ntracha 2020 - Keystroike features	0.78	0.60	0.96	0.02
Milne et al. (2018) - MACD	0.85	0.77	0.93	0.03
Adams et al. (2017)	0.97	0.92	1.00	0.04
oroojeni et al. (2019)	0.88	0.81	0.95	0.04
Chen et al. (2019)	0.80	0.73	0.87	0.04
Chen et al. (2019)	0.73	0.65	0.81	0.03
Iakovakis et al. (2019)	0.87	0.75	0.99	0.03
Iakovakis et al. (2019)	0.88	0.76	1.00	0.03
Stringer et al. (2017)	0.80	0.68	0.92	0.03
Stringer et al. (2017)	0.91	0.82	1.00	0.03
Memedi et al. (2013)	0.86	0.79	0.93	0.04
Lee et al. (2016)	0.92	0.88	0.96	0.04
Lee et al. (2016)	0.88	0.83	0.93	0.04
Printy et al. (2014)	0.91	0.83	0.99	0.03
**Clinical Assessment**	**0.86**	**0.83**	**0.89**	**0.52**
Mastoras et al. (2019) - Multivariate	0.89	0.76	1.00	0.12
Iakovakis et al. (2019)	0.77	0.69	0.85	0.29
Iakovakis et al. (2019)	0.73	0.65	0.81	0.26
Mastoras 2019 - Univariate (HT)	0.81	0.65	0.97	0.07
Iakovakis et al. 2018 (Rigidity)	0.80	0.68	0.92	0.14
Iakovakis et al. 2018 (Bradykinesia)	0.78	0.66	0.90	0.13
**Self Reports**	**0.78**	**0.73**	**0.84**	**0.48**
Combined effect size	0.82	0.74	0.90	

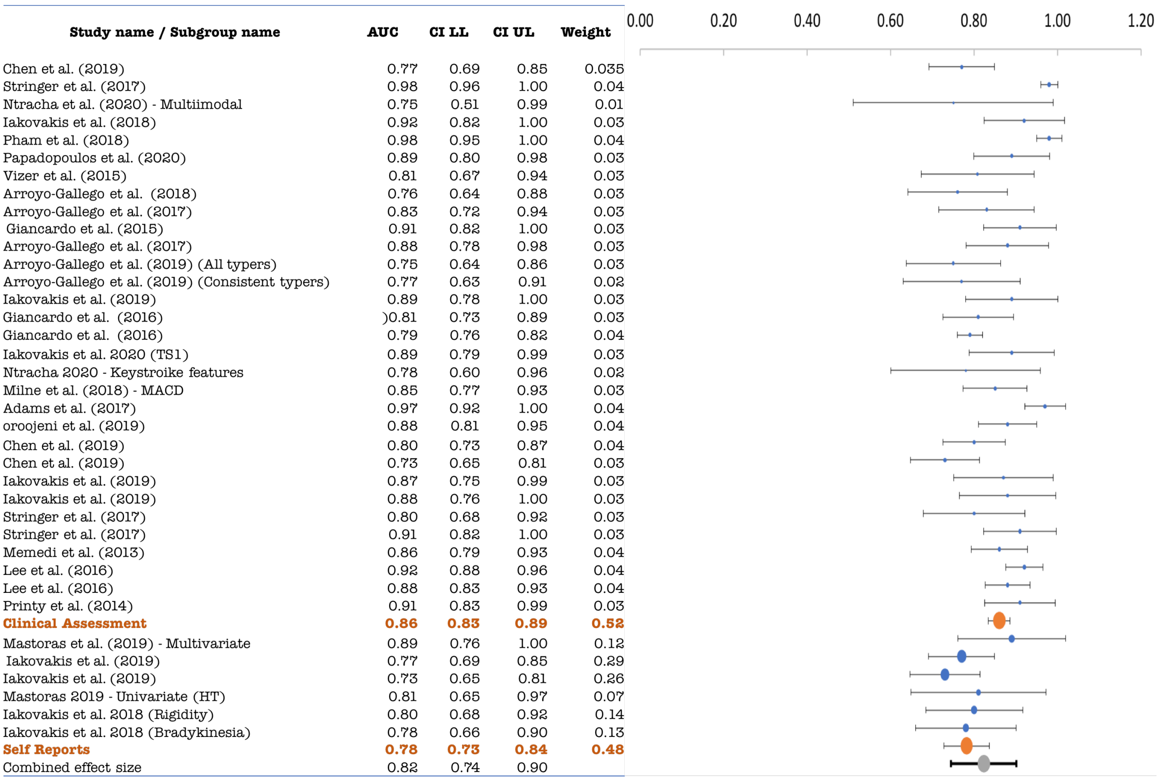

Figure S 9: Subgroup analysis results for AUC of data validated by clinical assessment vs. data labeled by self-reports.

Study name / Subgroup name	Accuracy	CI LL	CI UL	Weight
Chen et al. (2019)	0.80	0.73	0.88	0.047
Stringer et al. (2017)	0.93	0.87	0.99	0.06
Iakovakis et al. (2018)	0.91	0.81	1.00	0.03
Papadopoulos et al. (2020)	0.91	0.83	0.99	0.04
Vizer et al. (2015)	0.81	0.67	0.94	0.02
Arroyo-Gallego et al. (2018)	0.69	0.56	0.82	0.03
Arroyo-Gallego et al.(2017) - FT (raw)	0.70	0.56	0.84	0.02
Arroyo-Gallego et al. (2017) - Univariate Model	0.80	0.68	0.92	0.03
Arroyo-Gallego et al. (2017) - Multivariate Model	0.82	0.70	0.94	0.03
Iakovakis et al. (2019)	0.89	0.78	1.00	0.03
Giancardo et al. (2016)	0.78	0.69	0.87	0.04
Cao et al. (2017)	0.90	0.89	0.91	0.08
Iakovakis et al. (2020) TS1	0.85	0.73	0.96	0.03
Iakovakis et al. (2020) TS2	0.88	0.76	1.00	0.03
Adams et al. (2017)	0.94	0.87	1.00	0.05
Arora et al. (2018)	0.84	0.80	0.88	0.07
Arora et al. (2018)	0.90	0.86	0.94	0.07
oroojeni et al. (2019)	0.88	0.81	0.95	0.05
Pham 2018 - GLCM	0.92	0.86	0.98	0.06
Stringer et al. (2018)	0.91	0.82	1.00	0.04
Stringer et al. (2018)	0.72	0.58	0.86	0.02
Printy et al. (2014)	0.95	0.88	1.00	0.06
Ntracha et al. (2020)	0.76	0.54	0.98	0.01
Ntracha et al. (2020)	0.77	0.59	0.95	0.02
Iakovakis et al. (2019)	0.76	0.61	0.91	0.02
Iakovakis et al. (2019)	0.79	0.65	0.93	0.02
**Clinical Assessments**	**0.86**	**0.83**	**0.89**	**0.54**
Mastoras et al. (2019)	0.80	0.63	0.97	0.08
Iakovakis 2019	0.79	0.71	0.87	0.21
Papadopoulos 2020	0.86	0.81	0.91	0.29
Mastoras et al. (2019)	0.84	0.69	0.99	0.09
Iakovakis et al. (2018) R	0.79	0.67	0.91	0.13
Iakovakis et al. (2018) B	0.69	0.51	0.87	0.06
Iakovakis et al. (2018) R	0.67	0.50	0.84	0.07
Iakovakis et al. (2018) B	0.71	0.55	0.87	0.08
**Self Reports**	**0.79**	**0.74**	**0.85**	**0.46**
Combined effect size	0.83	0.76	0.90	

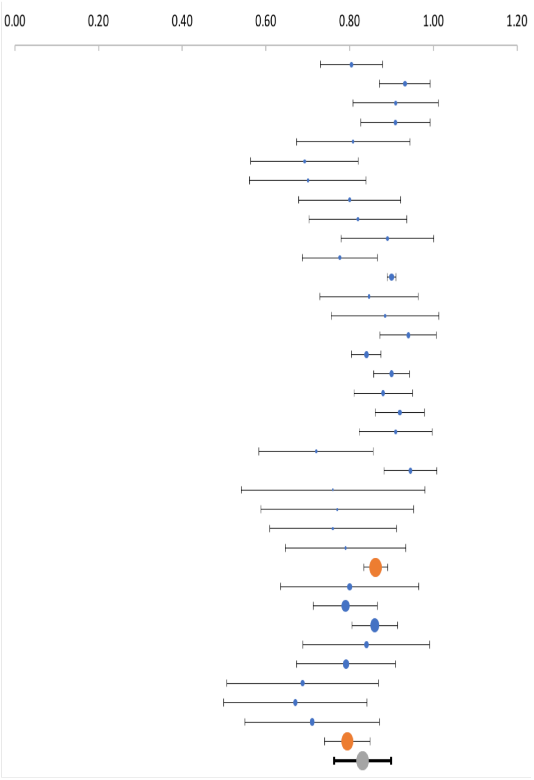

Figure S 10: Subgroup analysis results for Accuracy of data validated by clinical assessment vs. data labeled by self-reports.

Study name / Subgroup name	Sensitivity	CI LL	CI UL	Weight
Giancardo et al. (2016)	0.71	0.57	0.85	0.041
Alloyo et al. (2017) - Univariate Model	0.81	0.63	0.99	0.03
Alloyo et al. (2017) - Multivariate Model	0.73	0.53	0.93	0.03
Alloyo et al. (2017) - FT (raw)	0.73	0.51	0.95	0.03
Iakovakis et al. (2018)	0.82	0.63	1.00	0.03
Papadopoulos et al. (2020) - Sdata	0.93	0.86	0.99	0.07
Iakovakis et al. (2020) - TS1	0.90	0.80	1.00	0.05
Iakovakis et al. (2020) - TS2	0.93	0.86	1.00	0.07
Vizer et al. (201)	0.71	0.47	0.94	0.02
Stringer et al. (2018)	0.89	0.81	0.97	0.06
Milne et al.(2018)	0.64	0.49	0.79	0.04
Iakovakis et al. (2019)	0.79	0.59	0.99	0.03
Pahm et al. (2018)	0.98	0.94	1.00	0.07
Alloyo et al. (2018)	0.73	0.55	0.91	0.03
Adams (2017)	0.92	0.83	1.00	0.06
Arora et al. (2018)	0.85	0.81	0.89	0.07
Arora et al. (2018)	0.92	0.86	0.98	0.07
Pham et al. (2018)	0.99	0.96	1.00	0.07
Stringer et al. (2018)	0.84	0.74	0.94	0.05
Iakovakis et al. (2019)	0.75	0.56	0.94	0.03
Iakovakis et al. (2019)	0.78	0.61	0.95	0.04
**Clinical Assessment**	**0.86**	**0.81**	**0.90**	**0.66**
Iakovakis et al. (2018) - Rigidity	0.77	0.52	1.00	0.06
Iakovakis et al. (2018) - Bradykinesia	0.92	0.83	1.00	0.18
Papadopoulos et al. (2020) - Gdata	0.92	0.86	0.98	0.21
Iakovakis et al. (2019)	0.74	0.57	0.91	0.09
Mastroras et al. (2019) - Univariate	0.82	0.64	1.00	0.10
Mastroras et al. (2019) - Univariate	0.75	0.56	0.94	0.08
Iakovakis et al. (2018) - Rigidity	0.82	0.65	0.99	0.10
Iakovakis et al. (2018) - Bradykinesia	0.86	0.71	1.00	0.11
Iakovakis et al. (2019)	0.65	0.46	0.84	0.08
**Self Reports**	**0.83**	**0.76**	**0.90**	**0.34**
Combined effect size	0.85	0.83	0.87	

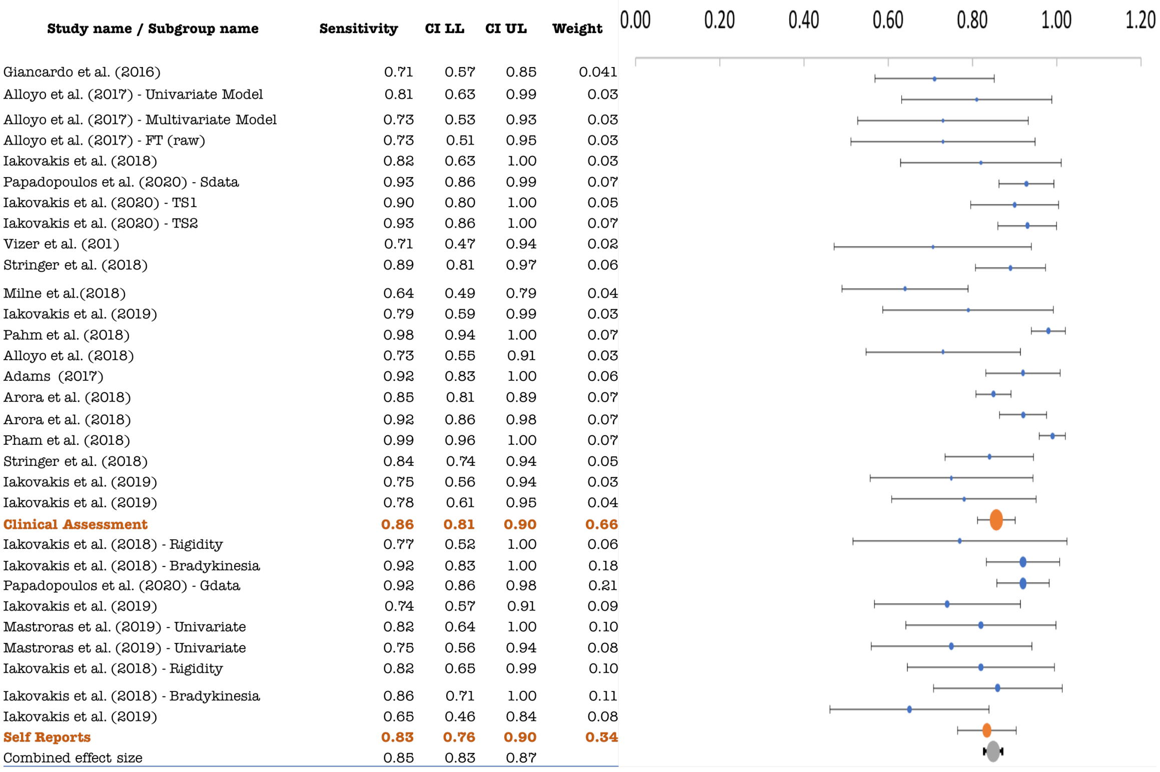

Figure S 11: Subgroup analysis results for Sensitivity of data validated by clinical assessment vs. data labeled by self-reports.

Study name / Subgroup name	Specificity	CI LL	CI UL	Weight
Giancardo et al. (2016)	0.84	0.73	0.95	0.046
Alloyo et al. (2017) - Univariate Model	0.81	0.64	0.98	0.03
Alloyo et al. (2017) - Multivariate Model	0.84	0.68	1.00	0.03
Alloyo et al. (2017) - FT	0.72	0.53	0.91	0.03
Iakovakis et al. (2018)	0.81	0.64	0.98	0.03
Papadopoulos et al. (2020) - Sdata	0.93	0.87	0.99	0.07
Iakovakis et al. (2020) - TS1	0.83	0.66	1.00	0.03
Iakovakis et al. (2020) - TS2	0.90	0.79	1.00	0.05
Vizer et al. (2015)	0.83	0.66	1.00	0.03
Stringer et al. (2018)	0.99	0.97	1.00	0.08
oroojeni et al. (2019)	0.84	0.73	0.95	0.05
Ntratcha et al. (2020)	0.64	0.34	0.94	0.01
Ntratcha et al. (2020)	0.90	0.79	1.00	0.05
Milne et al. (2018) - standard deviation	0.81	0.69	0.93	0.04
Iakovakis et al. (2019)	0.79	0.62	0.96	0.03
Alloyo et al. (2018)	0.69	0.51	0.87	0.03
Pahm et al. (2018)	0.96	0.92	1.00	0.08
Adams 2017	0.95	0.89	1.00	0.07
Arora et al. (2018)	0.88	0.81	0.95	0.06
Arora et al. (2018)	0.90	0.83	0.97	0.07
Pahm et al. (2018)	0.90	0.81	0.99	0.05
Stringer et al. (2018)	0.78	0.61	0.95	0.03
**Clinical Assessment**	**0.87**	**0.84**	**0.91**	**0.52**
Iakovakis et al. (2018) - Rigidity	0.80	0.66	0.94	0.08
Iakovakis et al. (2018) - Bradykinesia	0.63	0.46	0.80	0.07
Papadopoulos et al. (2020) - Gdata	0.69	0.61	0.77	0.11
Iakovakis et al. (2020) - TS3	0.71	0.64	0.78	0.11
Iakovakis et al. (2019)	0.78	0.69	0.87	0.10
Mastroras et al. (2019)	0.86	0.73	0.99	0.09
Mastroras et al. (2019)	0.82	0.65	0.99	0.08
Iakovakis et al. (2018) - Rigidity	0.65	0.49	0.81	0.07
Iakovakis et al. (2018) - Bradykinesia	0.60	0.43	0.77	0.07
Iakovakis et al.(2019)	0.92	0.86	0.98	0.11
Iakovakis et al. (2019)	0.78	0.69	0.87	0.10
**Self Reports**	**0.76**	**0.69**	**0.82**	**0.48**
Combined effect size	0.82	0.70	0.94	

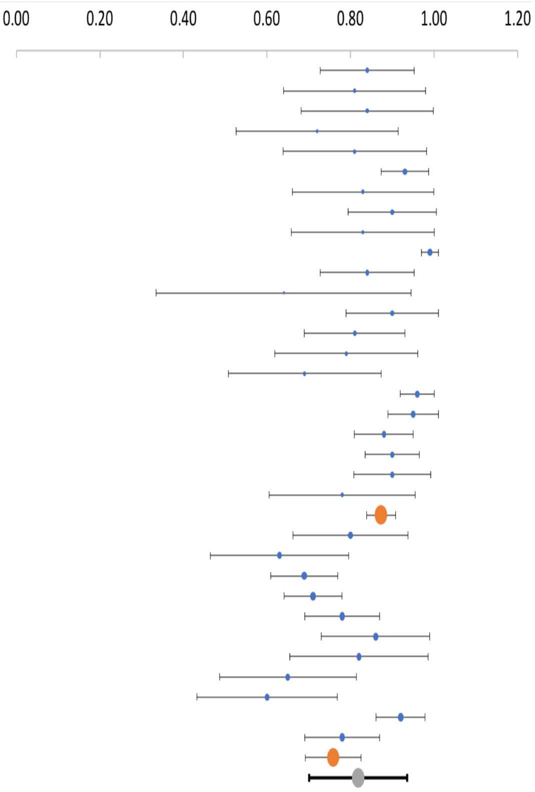

Figure S 12: Subgroup analysis results for Specificity of data validated by clinical assessment vs. data labeled by self-reports.

Study name / Subgroup name	AUC	CI LL	CI UL	Weight
Chen et al. (2019)	0.77	0.69	0.85	0.120
Stringer et al. (2017)	0.98	0.96	1.00	0.13
Ntracha et al. (2020)- Multiimodal	0.75	0.51	0.99	0.07
Papadopoulos et al. (2020)	0.89	0.80	0.98	0.12
Vizer et al. (2015)	0.81	0.67	0.94	0.10
Chen et al. (2019)	0.80	0.73	0.87	0.12
Chen et al. (2019)	0.73	0.65	0.81	0.12
Stringer et al. (2017)	0.80	0.68	0.92	0.11
Stringer et al. (2017)	0.91	0.82	1.00	0.12
**Multimodal Analysis**	**0.83**	**0.77**	**0.90**	**0.16**
Iakovakis et al. (2018)	0.92	0.82	1.00	0.03
Pham et al. (2018)	0.98	0.95	1.00	0.05
Mastoras et al. (2019) - Univariate	0.85	0.70	1.00	0.02
Arroyo-Gallego et al. (2018)	0.76	0.64	0.88	0.03
Arroyo-Gallego et al. (2017)	0.83	0.72	0.94	0.03
Arroyo-Gallego 2017	0.91	0.82	1.00	0.03
Arroyo-Gallego 2017	0.88	0.78	0.98	0.03
Arroyo-Gallego 2019 (All typers)	0.75	0.64	0.86	0.03
Arroyo-Gallego 2019 (Consistent typers)	0.77	0.63	0.91	0.02
Iakovakis et al. (2019)	0.89	0.78	1.00	0.03
Giancardo et al. (2016)	0.81	0.73	0.89	0.03
Iakovakis et al. (2020) (TS1)	0.89	0.79	0.99	0.03
Iakovakis et al. 2020 (TS2)	0.97	0.93	1.00	0.04
Iakovakis et al. 2020 (TS3)	0.79	0.74	0.84	0.04
Iakovakis et al. 2018 (Rigidity)	0.84	0.73	0.95	0.03
Iakovakis et al. 2018 (Bradykinesia)	0.80	0.68	0.92	0.03
Ntracha et al. (2020) - Keystroke features	0.78	0.60	0.96	0.02
Mastoras et al. (2019) - Multivariate	0.89	0.76	1.00	0.03
Milne et al. (2018)	0.85	0.77	0.93	0.04
oroojeni et al. (2019)	0.85	0.77	0.93	0.04
Adams et al. (2017)	0.97	0.92	1.00	0.04
oroojeni et al. (2019)	0.88	0.81	0.95	0.04
Iakovakis et al.(2019)	0.88	0.76	1.00	0.03
Iakovakis et al.(2019)	0.73	0.65	0.81	0.03
Mastoras et al. (2019) - Univariate (HT)	0.81	0.65	0.97	0.02
Memedi et al. (2013)	0.86	0.79	0.93	0.04
Lee et al. (2016)	0.92	0.88	0.96	0.04
Lee et al. (2016)	0.88	0.83	0.93	0.04
Printy et al. (2014)	0.91	0.83	0.99	0.04
Iakovakis et al. (2018) (Rigidity)	0.80	0.68	0.92	0.03
Iakovakis et al.(2018) (Bradykinesia)	0.78	0.66	0.90	0.03
**Unimodal Analysis**	**0.86**	**0.84**	**0.89**	**0.84**
Combined effect size	0.86	0.84	0.88	

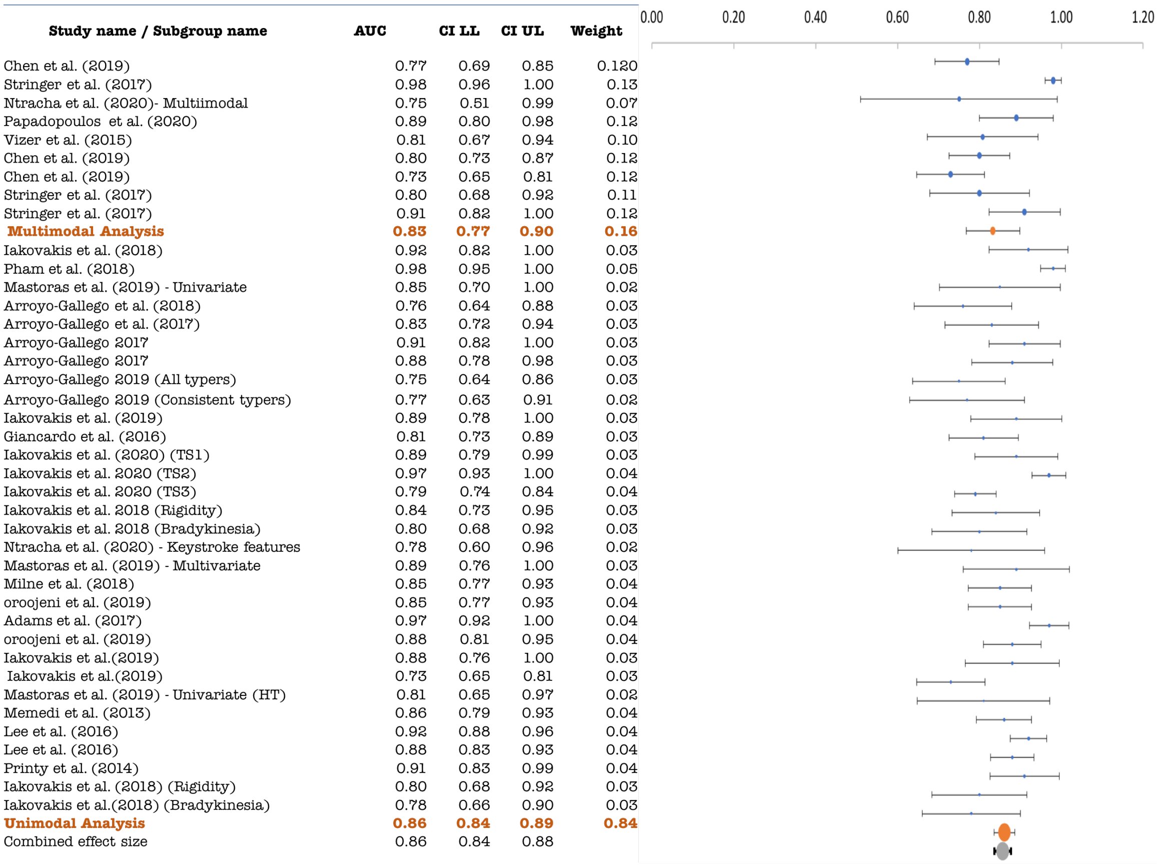

Figure S 13: Subgroup analysis results for AUC of multimodal analysis vs. unimodal analysis.

Study name / Subgroup name	Accuracy	CI LL	CI UL	Weight
Chen et al.(2019)	0.80	0.73	0.88	0.083
Stringer et al. (2017)	0.93	0.87	0.99	0.10
Papadopoulos et al. (2020)	0.91	0.83	0.99	0.08
Vizer et al. (2015)	0.81	0.67	0.94	0.04
Cao et al. (2017)	0.90	0.89	0.91	0.18
Papadopoulos et al.(2020)	0.86	0.81	0.91	0.11
Arora et al. (2018)	0.84	0.80	0.88	0.14
Arora et al. (2018)	0.90	0.86	0.94	0.13
Stringer et al. (2018)	0.91	0.82	1.00	0.07
Stringer et al. (2018)	0.72	0.58	0.86	0.04
Ntracha et al. (2020)	0.77	0.59	0.95	0.02
**Multimodal Analysis**	**0.87**	**0.83**	**0.91**	**0.50**
Iakovakis et al .(2018)	0.91	0.81	1.00	0.05
Mastoras et al. (2019)	0.80	0.63	0.97	0.03
Arroyo-Gallego et al. (2018)	0.69	0.56	0.82	0.04
Arroyo-Gallego et al. (2018)	0.75	0.63	0.87	0.04
Arroyo-Gallego et al. (2017) - FT (raw)	0.70	0.56	0.84	0.03
Arroyo-Gallego 2017 - Univariate Model	0.80	0.68	0.92	0.04
Arroyo-Gallego 2017 - Multivariate Model	0.82	0.70	0.94	0.04
Iakovakis et al. (2019)	0.89	0.78	1.00	0.04
Iakovakis et al. (2019)	0.79	0.71	0.87	0.05
Giancardo 2016	0.78	0.69	0.87	0.05
Iakovakis et al. (2020) TS1	0.85	0.73	0.96	0.04
Iakovakis et al. (2020) TS2	0.88	0.76	1.00	0.04
Milne et al. (2018) - FRESH	0.73	0.63	0.83	0.05
Adams 2017	0.94	0.87	1.00	0.06
oroojeni et al. (2019)	0.88	0.81	0.95	0.05
Pham 2018 - GLCM	0.92	0.86	0.98	0.06
Mastoras 2019	0.84	0.69	0.99	0.03
Printy et al. (2014)	0.95	0.88	1.00	0.06
Ntracha et al. (2020)	0.76	0.54	0.98	0.02
Iakovakis et al. (2018) R	0.79	0.67	0.91	0.04
Iakovakis et al. (2018) B	0.69	0.51	0.87	0.03
Iakovakis et al. (2018) R	0.67	0.50	0.84	0.03
Iakovakis et al. (2018) B	0.71	0.55	0.87	0.03
Iakovakis et al. (2019)	0.76	0.61	0.91	0.03
Iakovakis et al. (2019)	0.79	0.65	0.93	0.03
**Unimodal Analysis**	**0.82**	**0.78**	**0.85**	**0.50**
Combined effect size	0.84	0.79	0.90	

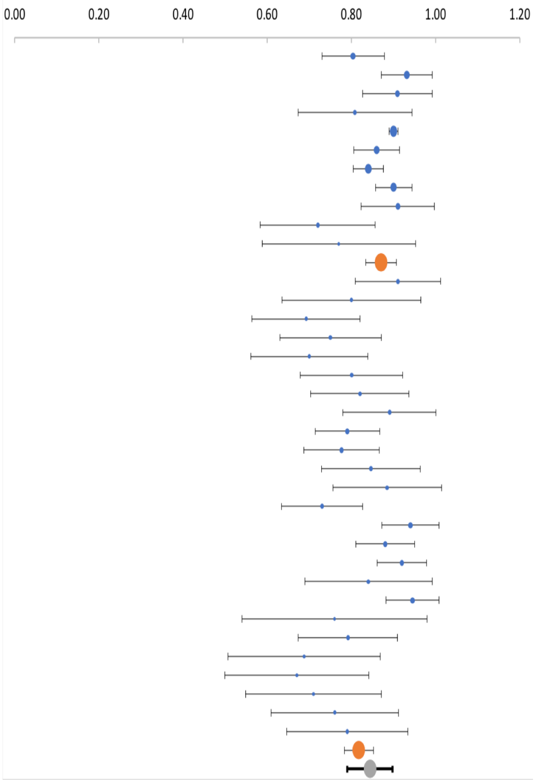

Figure S 14: Subgroup analysis results for Accuracy of multimodal analysis vs. unimodal analysis.

Study name / Subgroup name	Sensitivity	CI LL	CI UL	Weight
Papadopoulos et al. (2020) - Sdata	0.93	0.86	0.99	0.173
Papadopoulos et al. (2020) - Gdata	0.92	0.86	0.98	0.17
Vizer et al. (2015)	0.71	0.47	0.94	0.02
Stringer et al. (2018)	0.89	0.81	0.97	0.12
Arora et al. (2018)	0.85	0.81	0.89	0.23
Arora et al. (2018)	0.92	0.86	0.98	0.18
Stringer et al. (2018)	0.84	0.74	0.94	0.09
**Multimodal Analysis**	**0.89**	**0.84**	**0.93**	**0.52**
Giancardo et al. (2016)	0.71	0.57	0.85	0.05
Alloyo et al. (2017) - Univariate	0.81	0.63	0.99	0.04
Alloyo et al. (2017) - Multivariate	0.73	0.53	0.93	0.03
Alloyo et al. (2017) - FT (raw)	0.73	0.51	0.95	0.03
Iakovakis et al. (2018)	0.82	0.63	1.00	0.04
Iakovakis et al. (2018) - Rigidity	0.77	0.52	1.00	0.03
Iakovakis et al. (2018) -	0.92	0.83	1.00	0.06
Iakovakis et al. (2020) - TS1	0.90	0.80	1.00	0.06
Iakovakis et al. (2020) - TS2	0.93	0.86	1.00	0.07
Milne et al.(2018)	0.64	0.49	0.79	0.04
Iakovakis et al. (2019)	0.79	0.59	0.99	0.03
Iakovakis et al. (2019)	0.74	0.57	0.91	0.04
Pahm et al. (2018)	0.98	0.94	1.00	0.07
Alloyo et al. (2018)	0.73	0.55	0.91	0.04
Adams (2017)	0.92	0.83	1.00	0.06
Pham et al. (2018)	0.99	0.96	1.00	0.07
Mastroras et al. (2019) - Multivariate	0.82	0.64	1.00	0.04
Mastroras et al. (2019) - Univariate	0.75	0.56	0.94	0.04
Iakovakis et al. (2018) - Rigidity	0.82	0.65	0.99	0.04
Iakovakis et al. (2018) - Bradykinesia	0.86	0.71	1.00	0.05
Iakovakis et al. (2019)	0.75	0.56	0.94	0.04
Iakovakis et al. (2019)	0.78	0.61	0.95	0.04
**Unimodal Analysis**	**0.84**	**0.79**	**0.88**	**0.48**
Combined effect size	0.86	0.81	0.92	

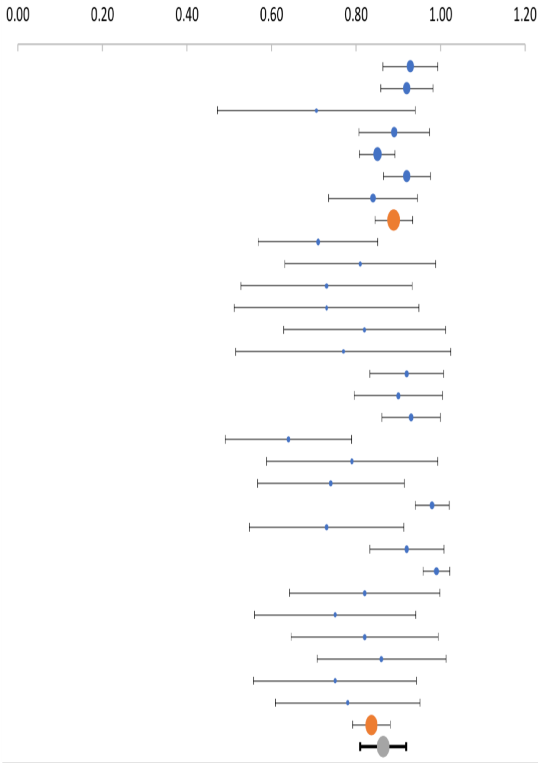

Figure S 15: Subgroup analysis results for Sensitivity of multimodal analysis vs. unimodal analysis.

Study name / Subgroup name	Specificity	CI LL	CI UL	Weight
Papadopoulos et al. (2020) - Sdata	0.93	0.87	0.99	0.142
Papadopoulos et al. (2020) - Gdata	0.69	0.61	0.77	0.13
Vizer et al. (2015)	0.83	0.66	1.00	0.09
Stringer et al. (2018)	0.99	0.97	1.01	0.15
Ntratcha et al. (2020)	0.90	0.79	1.01	0.12
Arora et al. (2018)	0.88	0.81	0.95	0.14
Arora et al. (2018)	0.90	0.83	0.97	0.14
Stringer et al. (2018)	0.78	0.61	0.95	0.09
**Multimodal Analysis**	**0.87**	**0.79**	**0.95**	**0.42**
Giancardo et al. (2016)	0.84	0.73	0.95	0.05
Alloyo et al. (2017) - Univariate Model	0.81	0.64	0.98	0.04
Alloyo et al. (2017) - Multivariate Model	0.84	0.68	1.00	0.04
Alloyo et al. (2017) - FT	0.72	0.53	0.91	0.03
Iakovakis et al. (2018)	0.81	0.64	0.98	0.04
Iakovakis et al. (2018) - Rigidity	0.80	0.66	0.94	0.04
Iakovakis et al. (2018) - Bradykinesia	0.63	0.46	0.80	0.04
Iakovakis et al. (2020) - TS1	0.83	0.66	1.00	0.04
Iakovakis et al. (2020) - TS2	0.90	0.79	1.01	0.05
Iakovakis et al. (2020) - TS3	0.71	0.64	0.78	0.06
Ntratcha et al. (2020)	0.64	0.34	0.94	0.02
Milne et al. (2018) - standard deviation	0.81	0.69	0.93	0.05
Iakovakis et al. (2019)	0.79	0.62	0.96	0.04
Iakovakis et al. (2019)	0.78	0.69	0.87	0.05
Alloyo et al. (2018)	0.69	0.51	0.87	0.03
Adams et al. 2017	0.95	0.89	1.01	0.06
Pahm et al. (2018)	0.90	0.81	0.99	0.05
Mastroras et al. (2019)	0.86	0.73	0.99	0.05
Mastroras et al. (2019)	0.82	0.65	0.99	0.04
Iakovakis et al. (2018) - Rigidity	0.65	0.49	0.81	0.04
Iakovakis et al. (2018) - Bradykinesia	0.60	0.43	0.77	0.04
Iakovakis et al.(2019)	0.92	0.86	0.98	0.06
Iakovakis et al. (2019)	0.78	0.69	0.87	0.05
**Unimodal Analysis**	**0.80**	**0.76**	**0.84**	**0.58**
**Combined effect size**	**0.83**	**0.76**	**0.90**	

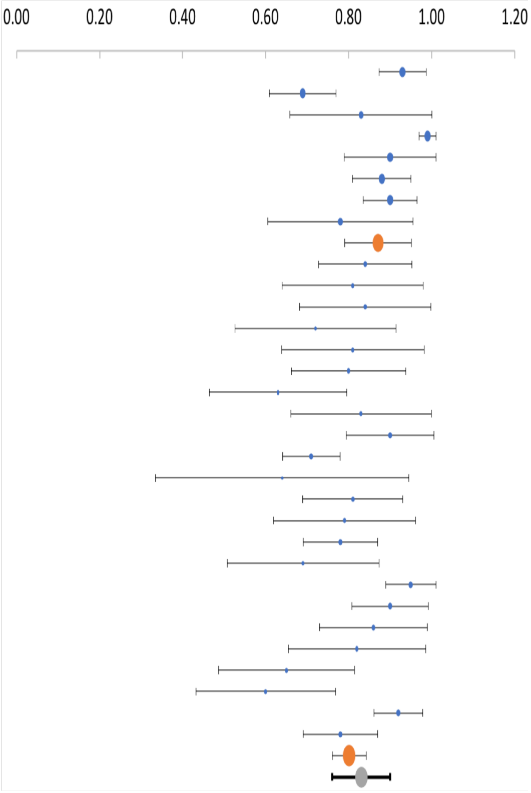

Figure S 16: Subgroup analysis results for Specificity of multimodal analysis vs. unimodal analysis.

Study name / Subgroup name	AUC	CI LL	CI UL	Weight
Papadopoulos et al. (2020)	0.89	0.80	0.98	0.139
Iakovakis 2019	0.89	0.78	1.00	0.13
Iakovakis et al. 2020 (TS1)	0.89	0.79	0.99	0.13
Iakovakis et al. 2020 (TS2)	0.97	0.93	1.00	0.17
Iakovakis et al. 2020 (TS3)	0.79	0.74	0.84	0.16
Iakovakis 2019	0.88	0.76	1.00	0.13
Iakovakis 2019	0.73	0.65	0.81	0.14
**Deep Learning**	**0.86**	**0.79**	**0.94**	**0.13**
Chen et al. (2019)	0.77	0.69	0.85	0.03
Pham et al. (2018)	0.98	0.94	1.00	0.04
Ntracha et al. (2020)- Multimodal	0.75	0.51	0.99	0.01
Iakovakis et al. (2018)	0.92	0.82	1.00	0.03
Pham et al. (2018)	0.98	0.95	1.00	0.04
Mastoras et al. (2019) - Univariate	0.85	0.70	1.00	0.02
Vizer et al. (2015)	0.81	0.67	0.94	0.02
Arroyo-Gallego. et al. (2018)	0.76	0.64	0.88	0.03
Arroyo-Gallego et al. (2017)	0.83	0.72	0.94	0.03
Arroyo-Gallego et al. (2017)	0.91	0.82	1.00	0.03
Arroyo-Gallego et al. (2017)	0.88	0.78	0.98	0.03
Arroyo-Gallego et al. (2019) (All typers)	0.75	0.64	0.86	0.03
Arroyo-Gallego et al. (2019) (Consistent typers)	0.77	0.63	0.91	0.02
Giancardo et al. 2016	0.81	0.73	0.89	0.03
Iakovakis et al. 2018 (Rigidity -52 weeks)	0.84	0.73	0.95	0.03
Iakovakis et al. 2018 (Bradykinesia - 52 weeks)	0.80	0.68	0.92	0.03
Ntracha et al. (2020) - Keystroike features	0.78	0.60	0.96	0.02
Mastoras et al. (2019) - Multivariate	0.89	0.76	1.00	0.02
Milne et al. (2018) - MACD	0.85	0.77	0.93	0.03
oroojeni et al. (2019)	0.85	0.77	0.93	0.03
Adams et al. (2017)	0.97	0.92	1.00	0.04
oroojeni et al. (2019)	0.88	0.81	0.95	0.04
Chen et al. (2019)	0.77	0.69	0.85	0.03
Chen et al. (2019)	0.80	0.73	0.87	0.04
Stringer et al. (2017)	0.80	0.68	0.92	0.03
Iakovakis et al. (2019)	0.91	0.82	1.00	0.03
Mastoras 2019 - Univariate (HT)	0.81	0.65	0.97	0.02
Memedi et al. (2013)	0.86	0.79	0.93	0.04
Lee et al. (2016)	0.92	0.88	0.96	0.04
Lee et al. (2016)	0.88	0.83	0.93	0.04
Printy et al. (2014)	0.91	0.83	0.99	0.03
Iakovakis et al. 2018 (Rigidity – 1 week)	0.80	0.68	0.92	0.03
Iakovakis et al. 2018 (Bradykinesia – 1 week)	0.78	0.66	0.90	0.03
**Machine Learning**	**0.86**	**0.83**	**0.88**	**0.87**
Combined effect size	0.86	0.85	0.86	

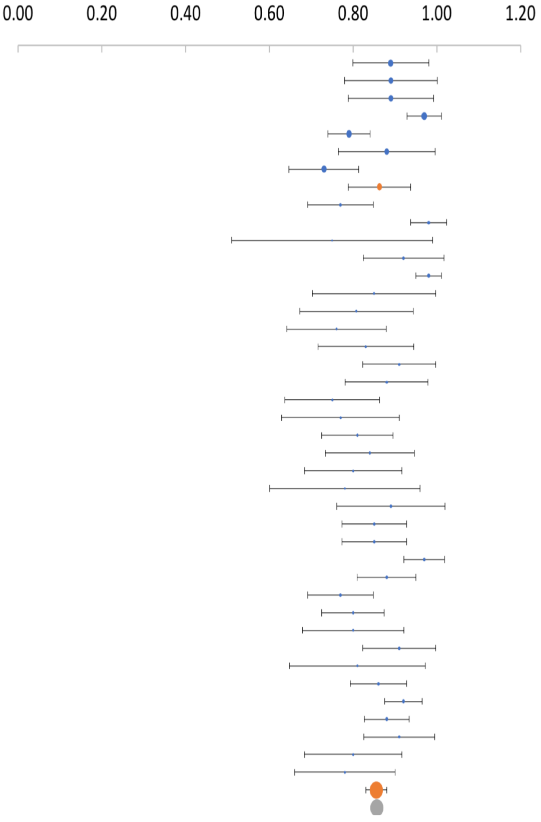

Figure S 17: Subgroup analysis results for AUC of deep learning vs. other machine learning methods.

Study name / Subgroup name	Accuracy	CI LL	CI UL	Weight
Papadopoulos et al. (2020)	0.91	0.83	0.99	0.145
Iakovakis et al. (2019)	0.89	0.78	1.00	0.10
Iakovakis et al. (2019)	0.79	0.71	0.87	0.15
Cao et al. (2017)	0.90	0.89	0.91	0.29
Iakovakis et al. (2020) TS1	0.85	0.73	0.96	0.09
Iakovakis et al. (2020) TS2	0.88	0.76	1.00	0.08
Iakovakis et al. (2019)	0.76	0.61	0.91	0.06
Iakovakis et al. (2019)	0.79	0.65	0.93	0.07
**Deep Learning**	**0.86**	**0.81**	**0.91**	**0.43**
Chen et al. (2019)	0.80	0.73	0.88	0.05
Stringer et al. (2017)	0.93	0.87	0.99	0.06
Iakovakis et al. (2018)	0.91	0.81	1.00	0.04
Mastoras et. al. (2019) -univariate	0.80	0.63	0.97	0.02
Vizer et al. (2015)	0.81	0.67	0.94	0.03
Arroyo-Gallego et al. (2018)	0.69	0.56	0.82	0.03
Arroyo-Gallego et al. (2018)	0.75	0.63	0.87	0.03
Arroyo-Gallego et al. (2017) - FT (raw)	0.70	0.56	0.84	0.03
Arroyo-Gallego et al. (2017) - Univariate Model	0.80	0.68	0.92	0.03
Arroyo-Gallego et al. (2017) - Multivariate Model	0.82	0.70	0.94	0.04
Giancardo et al. (2016)	0.78	0.69	0.87	0.04
Adams. et al. (2017	0.94	0.87	1.00	0.05
Arora et al. (2018)	0.84	0.80	0.88	0.06
Arora et al. (2018)	0.90	0.86	0.94	0.06
oroojeni et al. (2019)	0.88	0.81	0.95	0.05
Pham et al. (2018)- GLCM	0.92	0.86	0.98	0.06
Stringer et al. (2018)	0.91	0.82	1.00	0.05
Stringer et al. (2018)	0.72	0.58	0.86	0.03
Mastoras et al. (2019)-multivariate	0.84	0.69	0.99	0.03
Printy et al. (2014)	0.95	0.88	1.00	0.06
Ntracha et al. (2020)	0.76	0.54	0.98	0.02
Ntracha et al. (2020)	0.77	0.59	0.95	0.02
Iakovakis et al. (2018) R	0.79	0.67	0.91	0.04
Iakovakis et al. (2018) B	0.69	0.51	0.87	0.02
Iakovakis et al. (2018) R	0.67	0.50	0.84	0.02
Iakovakis et al. (2018) B	0.71	0.55	0.87	0.02
**Machine Learning**	**0.83**	**0.80**	**0.87**	**0.57**
Combined effect size	0.84	0.82	0.87	

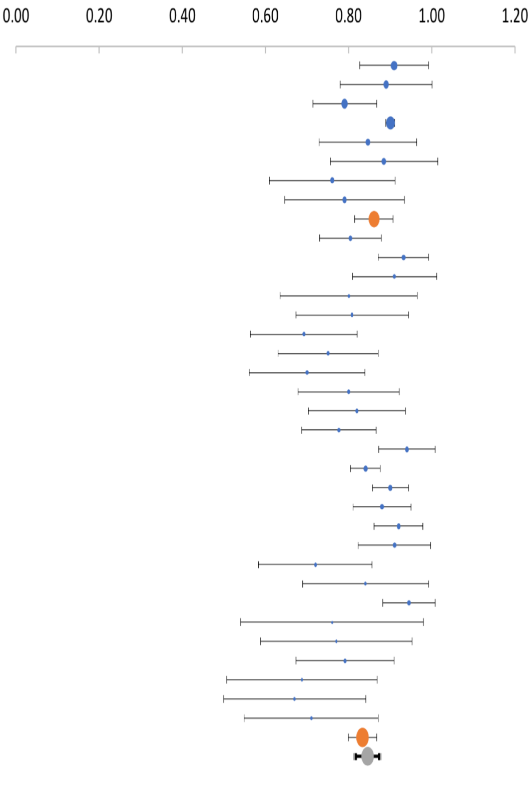

Figure S 18: Subgroup analysis results for Accuracy of deep learning vs. other machine learning methods.

Study name / Subgroup name	Sensitivity	CI LL	CI UL	Weight
Papadopoulos et al. (2020) - Sdata	0.93	0.86	0.99	0.226
Papadopoulos et al. (2020) - Gdata	0.92	0.86	0.98	0.23
Iakovakis et al. (2020) - TS1	0.90	0.80	1.00	0.14
Iakovakis et al. (2020) - TS2	0.93	0.86	1.00	0.23
Iakovakis et al. (2019)	0.74	0.57	0.91	0.06
Iakovakis et al. (2019)	0.75	0.56	0.94	0.06
Iakovakis et al. (2019)	0.78	0.61	0.95	0.07
**Deep Learning**	**0.89**	**0.83**	**0.96**	**0.47**
Giancardo et al. (2016)	0.71	0.57	0.85	0.04
Alloyo et al. (2017) - Univariate Model	0.81	0.63	0.99	0.04
Alloyo et al. (2017) - Multivariate Model	0.73	0.53	0.93	0.03
Alloyo et al. (2017) - FT (raw)	0.73	0.51	0.95	0.03
Iakovakis et al. (2018)	0.82	0.63	1.00	0.03
Iakovakis et al. (2018) - Rigidity	0.77	0.52	1.00	0.02
Iakovakis et al. (2018) - Bradykinesia	0.92	0.83	1.00	0.06
Vizer et al. (201)	0.71	0.47	0.94	0.03
Stringer et al. (2018)	0.89	0.81	0.97	0.06
oroojeni et al. (2019)	0.74	0.60	0.88	0.04
Milne et al.(2018) - Standard deviation	0.64	0.49	0.79	0.04
Iakovakis et al. (2019)	0.79	0.59	0.99	0.03
Pahm et al. (2018)	0.98	0.94	1.00	0.07
Alloyo et al. (2018)	0.73	0.55	0.91	0.03
Adams et al. (2017)	0.92	0.83	1.00	0.06
Arora et al. (2018)	0.85	0.81	0.89	0.07
Arora et al. (2018)	0.92	0.86	0.98	0.07
Stringer et al. (2018)	0.84	0.74	0.94	0.06
Stringer et al. (2018)	0.55	0.32	0.78	0.03
Mastoras et al. (2019) - Univariate	0.82	0.64	1.00	0.04
Mastoras et al. (2019) - Univariate	0.75	0.56	0.94	0.03
Iakovakis et al. (2018) - Rigidity	0.82	0.65	0.99	0.04
Iakovakis et al. (2018) - Bradykinesia	0.86	0.71	1.00	0.04
**Machine Learning**	**0.82**	**0.78**	**0.86**	**0.53**
Combined effect size	0.85	0.78	0.93	

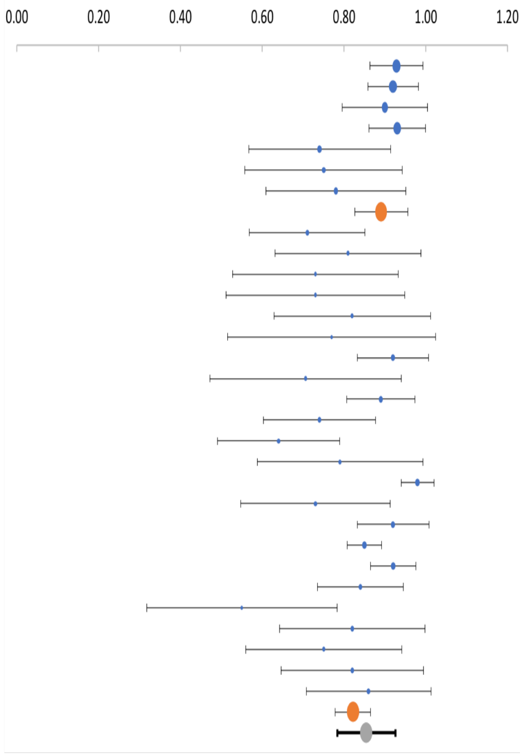

Figure S 19: Subgroup analysis results for Sensitivity of deep learning vs. other machine learning methods.

Study name / Subgroup name	Specificity	CI LL	CI UL	Weight
Papadopoulos et al. (2020) - Sdata	0.93	0.87	0.99	0.150
Papadopoulos et al. (2020) - Gdata	0.69	0.61	0.77	0.14
Iakovakis et al. (2020) - TS1	0.83	0.66	1.00	0.09
Iakovakis et al. (2020) - TS2	0.90	0.79	1.00	0.12
Iakovakis et al. (2019)	0.79	0.62	0.96	0.09
Iakovakis et al. (2019)	0.78	0.69	0.87	0.13
Iakovakis et al.(2019)	0.92	0.86	0.98	0.15
Iakovakis et al. (2019)	0.78	0.69	0.87	0.13
**Deep Learning**	**0.83**	**0.76**	**0.91**	**0.31**
Giancardo et al. (2016)	0.84	0.73	0.95	0.04
Alloyo et al. (2017) - Univariate Model	0.81	0.64	0.98	0.03
Alloyo et al. (2017) - Multivariate Model	0.84	0.68	1.00	0.03
Alloyo et al. (2017) - FT	0.72	0.53	0.91	0.03
Iakovakis et al. (2018)	0.81	0.59	1.00	0.03
Iakovakis et al. (2018) - Rigidity	0.80	0.66	0.94	0.04
Iakovakis et al. (2018) - Bradykinesia	0.63	0.46	0.80	0.03
Vizer et al. (2015)	0.83	0.66	1.00	0.03
Stringer et al. (2018)	0.99	0.95	1.00	0.06
Oroojeni et al. (2019)	0.84	0.73	0.95	0.04
Ntratcha et al. (2020)	0.64	0.34	0.94	0.02
Ntratcha et al. (2020)	0.90	0.79	1.00	0.05
Milne et al. (2018)	0.81	0.69	0.93	0.04
Alloyo et al. (2018)	0.69	0.51	0.87	0.03
Pahm et al. (2018)	0.96	0.92	1.00	0.06
Alloyo et al. (2018)	0.69	0.51	0.87	0.03
Adams 2017	0.95	0.89	1.00	0.05
Arora et al. (2018)	0.88	0.81	0.95	0.05
Arora et al. (2018)	0.90	0.83	0.97	0.05
Pahm et al. (2018)	0.90	0.81	0.99	0.05
Stringer et al. (2018)	0.78	0.61	0.95	0.03
Stringer et al. (2018)	0.67	0.47	0.87	0.03
Mastoras et al. (2019) - multivariate	0.86	0.73	0.99	0.04
Mastoras et al. (2019) -univariate	0.82	0.65	0.99	0.04
Iakovakis et al. (2018) - Rigidity - 1 week	0.65	0.49	0.81	0.03
Iakovakis et al. (2018) - Bradykinesia – 1 week	0.60	0.43	0.77	0.03
**Machine Learning**	**0.83**	**0.78**	**0.87**	**0.69**
Combined effect size	0.83	0.82	0.83	

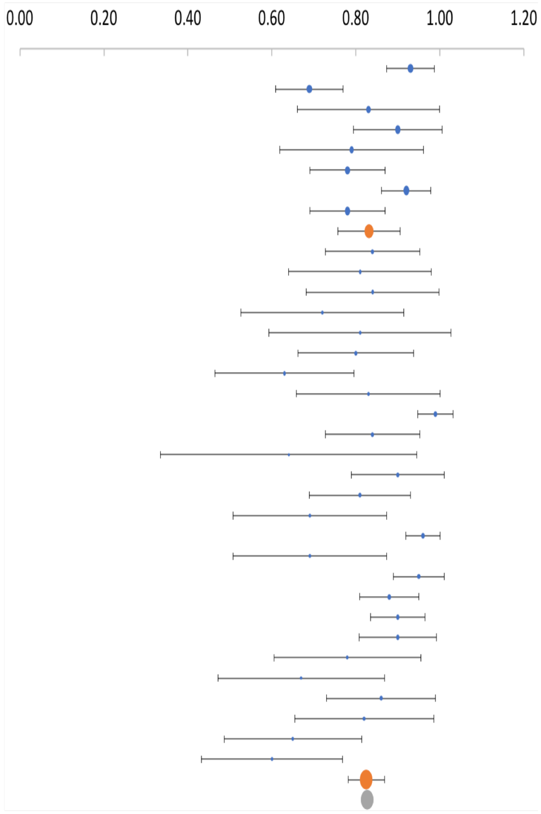

Figure S 20: Subgroup analysis results for Specificity of deep learning vs. other machine learning methods.

7 Regression Results

SUMMARY OUTPUT

Regression Statistics	
Multiple R	0.66727879
R Square	0.44526098
Adjusted R Square	0.39483016
Standard Error	1.33305639
Observations	13

ANOVA

	df	SS	MS	F	Significance F
Regression	1	15.6897366	15.6897366	8.8291443	0.01271377
Residual	11	19.5474326	1.77703933		
Total	12	35.2371692			

	Coefficients	Standard Error	t Stat	P-value	Lower 95%	Upper 95%	Lower 95.0%	Upper 95.0%
Intercept	-21.141407	8.28716765	-2.5511017	0.02694219	-39.38134	-2.9014742	-39.38134	-2.9014742
X Variable 1	0.39569202	0.13316742	2.97138761	0.01271377	0.10259251	0.68879154	0.10259251	0.68879154

Supplementary Table 9: Regression Results for PD patients' age and disease duration.

SUMMARY OUTPUT

Regression Statistics	
Multiple R	0.74607931
R Square	0.55663434
Adjusted R Square	0.51229777
Standard Error	0.05856767
Observations	12

ANOVA

	df	SS	MS	F	Significance F
Regression	1	0.04306494	0.04306494	12.5547463	0.00532766
Residual	10	0.03430172	0.00343017		
Total	11	0.07736667			

	Coefficients	Standard Error	t Stat	P-value	Lower 95%	Upper 95%	Lower 95.0%	Upper 95.0%
Intercept	0.68326545	0.03998807	17.0867311	9.9417E-09	0.59416647	0.77236443	0.59416647	0.77236443
X Variable 1	0.03743476	0.01056504	3.54326774	0.00532766	0.01389439	0.06097513	0.01389439	0.06097513

Supplementary Table 10: Regression Results for PD patients' disease duration and AUC.

SUMMARY OUTPUT

Regression Statistics	
Multiple R	0.68231434
R Square	0.46555286
Adjusted R Square	0.41696676
Standard Error	0.04723583
Observations	13

ANOVA

	df	SS	MS	F	Significance F
Regression	1	0.02137962	0.02137962	9.58201693	0.0101859
Residual	11	0.02454346	0.00223122		
Total	12	0.04592308			

	Coefficients	Standard Error	t Stat	P-value	Lower 95%	Upper 95%	Lower 95.0%	Upper 95.0%
Intercept	0.78841039	0.03077628	25.6174728	3.7025E-11	0.72067227	0.85614852	0.72067227	0.85614852
X Variable 1	0.02312079	0.0074692	3.09548331	0.0101859	0.00668119	0.0395604	0.00668119	0.0395604

Supplementary Table 11: Regression Results for PD patients' fine motor impairment index and AUC.

SUMMARY OUTPUT

Regression Statistics	
Multiple R	0.94080681
R Square	0.88511746
Adjusted R Square	0.84682328
Standard Error	0.01193542
Observations	5

ANOVA

	df	SS	MS	F	Significance F
Regression	1	0.00329264	0.00329264	23.1136287	0.01713355
Residual	3	0.00042736	0.00014245		
Total	4	0.00372			

	Coefficients	Standard Error	t Stat	P-value	Lower 95%	Upper 95%	Lower 95.0%	Upper 95.0%
Intercept	0.48785446	0.06224393	7.83778354	0.00432522	0.28976649	0.68594243	0.28976649	0.68594243
X Variable 1	0.00420516	0.00087468	4.80766353	0.01713355	0.00142154	0.00698877	0.00142154	0.00698877

Supplementary Table 12: Regression Results for MCI patients' age and AUC.

8 Key Points

What Was Known:

- Given the mildness of early stage symptoms, just-on-time detection of neurodegenerative and their associated psychiatric signs remains a challenge.
- Ubiquitous technology and connected devices facilitate the acquisition of high-frequency behavioral data that may aid in early diagnosis and monitoring

What this Paper Adds:

- Employing keyboard interactions, for detecting motor abnormalities, offers *unbiased diagnostic performance*, consistently across an array of neurodegenerative and psychiatric disorders, particularly PD, MCI and Depression.
- The diagnosis accuracy is significantly higher for data collected and validated *in-the-clinic*, compared to data collected *in-the-wild*, while the latter still results in acceptable accuracy.
- The adoption of multimodal data and deep learning models outperforms unimodal analysis, reflecting the importance of simultaneous learning of behavioral trends from aligned data streams
- Preliminary results on longitudinal behavioral analysis and treatment response are promising, yet this domain is still in its infancy.
- Despite the disproportionate utilization of keystroke dynamics analysis for PD, their reproducibility motivated using them for analyzing motor symptoms in other disorders, like AD, depression, multiple sclerosis and Huntington's disease.
- Neuropsychiatric disorders, despite the underlying diagnosis, are characterized by highly unstable typing latencies and more dispersed features, such as the hold time and the flight time.
- Considerable similarities between symptoms exist, entailing further research to better understand disease nosology and decipher disease-specific behavioral traits.
- The immature adoption of digital biomarkers in the clinical workflow requires an urgent multidisciplinary collaboration, realized by a *co-creation* approach, whereby relevant stakeholders contribute with the final objective of disease diagnosis and early intervention.
- Ideal future path implies undertaking cross-sectional, observational studies with a full representation of the population and longitudinal studies, to inform better understanding of disease nosology and behavioral trajectories aligned with disease prognosis respectively.

References

1. Giancardo, L. *et al.* Computer keyboard interaction as an indicator of early parkinson's disease. *Scientific reports* **6**, 1–10 (2016).
2. Arroyo-Gallego, T. *et al.* Detection of motor impairment in parkinson's disease via mobile touchscreen typing. *IEEE Transactions on Biomedical Engineering* **64**, 1994–2002 (2017).
3. Arroyo-Gallego, T. *et al.* Detecting motor impairment in early parkinson's disease via natural typing interaction with keyboards: Validation of the neuroqwerty approach in an uncontrolled at-home setting. *Journal of medical Internet research* **20**, e89 (2018).
4. Iakovakis, D. *et al.* Touchscreen typing-pattern analysis for detecting fine motor skills decline in early-stage parkinson's disease. *Scientific reports* **8**, 1–13 (2018).
5. Iakovakis, D. *et al.* Motor impairment estimates via touchscreen typing dynamics toward parkinson's disease detection from data harvested in-the-wild. *Frontiers in ICT* **5**, 28 (2018).
6. Matarazzo, M. *et al.* Remote monitoring of treatment response in parkinson's disease: the habit of typing on a computer. *Movement Disorders* **34**, 1488–1495 (2019).
7. Prince, J., Arora, S. & de Vos, M. Big data in parkinson's disease: using smartphones to remotely detect longitudinal disease phenotypes. *Physiological measurement* **39**, 044005 (2018).
8. Lipsmeier, F. *et al.* Evaluation of smartphone-based testing to generate exploratory outcome measures in a phase 1 parkinson's disease clinical trial. *Movement Disorders* **33**, 1287–1297 (2018).
9. Chen, R. *et al.* Developing measures of cognitive impairment in the real world from consumer-grade multimodal sensor streams. In *Proceedings of the 25th ACM SIGKDD International Conference on Knowledge Discovery & Data Mining*, 2145–2155 (2019).

10. Stringer, G. *et al.* Can you detect early dementia from an email? a proof of principle study of daily computer use to detect cognitive and functional decline. *International journal of geriatric psychiatry* **33**, 867–874 (2018).

11. Ntracha, A. *et al.* Detection of mild cognitive impairment through natural language and touchscreen typing processing. front. digit. *Health* **2**, 567158 (2020).

12. Vizer, L. M. & Sears, A. Classifying text-based computer interactions for health monitoring. *IEEE Pervasive Computing* **14**, 64–71 (2015).

13. Rabinowitz, I. & Lavner, Y. Association between finger tapping, attention, memory, and cognitive diagnosis in elderly patients. *Perceptual and motor skills* **119**, 259–278 (2014).

14. Van Waes, L., Leijten, M., Mariën, P. & Engelborghs, S. Typing competencies in alzheimer's disease: An exploration of copy tasks. *Computers in Human Behavior* **73**, 311–319 (2017).

15. Lee, C. Y. *et al.* A validation study of a smartphone-based finger tapping application for quantitative assessment of bradykinesia in parkinson's disease. *PloS one* **11**, e0158852 (2016).

16. Arora, S. *et al.* Smartphone motor testing to distinguish idiopathic rem sleep behavior disorder, controls, and pd. *Neurology* **91**, e1528–e1538 (2018).

17. Memedi, M., Khan, T., Grenholm, P., Nyholm, D. & Westin, J. Automatic and objective assessment of alternating tapping performance in parkinson's disease. *Sensors* **13**, 16965–16984 (2013).

18. Zhan, A. *et al.* High frequency remote monitoring of parkinson's disease via smartphone: Platform overview and medication response detection. *arXiv preprint arXiv:1601.00960* (2016).

19. Printy, B. P. *et al.* Smartphone application for classification of motor impairment severity in parkinson's disease. In *2014 36th Annual International Conference of the IEEE Engineering in Medicine and Biology Society*, 2686–2689 (IEEE, 2014).

20. Huang, H., Cao, B., Phillip, S. Y., Wang, C.-D. & Leow, A. D. Dpmood: Exploiting local and periodic typing dynamics for personalized mood prediction. In *2018 IEEE International Conference on Data Mining (ICDM)*, 157–166 (IEEE, 2018).

21. Cao, B. *et al.* Deepmood: modeling mobile phone typing dynamics for mood detection. In *Proceedings of the 23rd ACM SIGKDD International Conference on Knowledge Discovery and Data Mining*, 747–755 (2017).

22. Pham, T. D. Pattern analysis of computer keystroke time series in healthy control and early-stage parkinson's disease subjects using fuzzy recurrence and scalable recurrence network features. *Journal of neuroscience methods* **307**, 194–202 (2018).

23. Pham, T. D., Wårdell, K., Eklund, A. & Salerud, G. Classification of short time series in early parkinson s disease with deep learning of fuzzy recurrence plots. *IEEE/CAA Journal of Automatica Sinica* **6**, 1306–1317 (2019).

24. Milne, A., Farrahi, K. & Nicolaou, M. A. Less is more: Univariate modelling to detect early parkinson's disease from keystroke dynamics. In *International Conference on Discovery Science*, 435–446 (Springer, 2018).

25. Papadopoulos, A. *et al.* Unobtrusive detection of parkinson's disease from multi-modal and in-the-wild sensor data using deep learning techniques. *Scientific Reports* **10**, 1–13 (2020).

26. Mastoras, R.-E. *et al.* Touchscreen typing pattern analysis for remote detection of the depressive tendency. *Scientific reports* **9**, 1–12 (2019).

27. Iakovakis, D. *et al.* Early parkinson's disease detection via touchscreen typing analysis using convolutional neural networks. In *2019 41st Annual International Conference of the IEEE Engineering in Medicine and Biology Society (EMBC)*, 3535–3538 (IEEE, 2019).

28. Iakovakis, D. *et al.* Screening of parkinsonian subtle fine-motor impairment from touchscreen typing via deep learning. *Scientific reports* **10**, 1–13 (2020).

29. Wissel, B. D. *et al.* Tablet-based application for objective measurement of motor fluctuations in parkinson disease. *Digital biomarkers* **1**, 126–135 (2017).

30. Adams, W. R. High-accuracy detection of early parkinson's disease using multiple characteristics of finger movement while typing. *PloS one* **12**, e0188226 (2017).

31. Zulueta, J. *et al.* Predicting mood disturbance severity with mobile phone keystroke metadata: A biaffect digital phenotyping study. *Journal of medical Internet research* **20**, e241 (2018).

32. Stange, J. P. *et al.* Let your fingers do the talking: Passive typing instability predicts future mood outcomes. *Bipolar disorders* **20**, 285–288 (2018).

33. Vesel, C. *et al.* Effects of mood and aging on keystroke dynamics metadata and their diurnal patterns in a large open-science sample: A biaffect ios study. *Journal of the American Medical Informatics Association* **27**, 1007–1018 (2020).

34. Giancardo, L., Sánchez-Ferro, A., Butterworth, I., Mendoza, C. & Hooker, J. M. Psychomotor impairment detection via finger interactions with a computer keyboard during natural typing. *Scientific reports* **5**, 1–8 (2015).

35. Surangsrirat, D., Sri-Iesaranusorn, P., Chaiyaroj, A., Vateekul, P. & Bhidayasiri, R. Parkinson's disease severity clustering based on tapping activity on mobile device. *Scientific Reports* **12**, 1–11 (2022).

36. Wang, Y. *et al.* Facilitating text entry on smartphones with qwerty keyboard for users with parkinson's disease. In *Proceedings of the 2021 CHI Conference on Human Factors in Computing Systems*, 1–12 (2021).

37. Goñi, M., Eickhoff, S. B., Far, M. S., Patil, K. R. & Dukart, J. Smartphone-based digital biomarkers for parkinson's disease in a remotely-administered setting. *IEEE Access* (2022).

38. Zulueta, J. *et al.* The effects of bipolar disorder risk on a mobile phone keystroke dynamics based biomarker of brain age. *Frontiers in Psychiatry* **12** (2021).

39. Ross, M. K. *et al.* Naturalistic smartphone keyboard typing reflects processing speed and executive function. *Brain and behavior* **11**, e2363 (2021).

40. Hooman, O. M., Oldfield, J. & Nicolaou, M. A. Detecting early parkinson's disease from keystroke dynamics using the tensor-train decomposition. In *2019 27th European Signal Processing Conference (EUSIPCO)*, 1–5 (IEEE, 2019).
