# Supplementary material for: Diagnostic accuracy of keystroke dynamics as digital biomarkers for fine motor decline in neuropsychiatric disorders: a systematic review and meta-analysis
Source: Sci Rep. 2022 May 11;12:7690. doi: 10.1038/s41598-022-11865-7 (PMC9095860; doi:10.1038/s41598-022-11865-7)
Supplement: Supplementary file 2 — Supplementary Information 2. [file 41598_2022_11865_MOESM2_ESM.pdf]

PRISMA 2020 Checklist

Section and Topic	Item #	Checklist item	Location where item is reported
**TITLE**			
Title	1	Diagnostic Accuracy of Keystroke Dynamics as Digital Biomarkers for Fine Motor Decline in Neuropsychiatric Disorders: A Systematic Review and Meta-Analysis	Page 1
**ABSTRACT**			
Abstract	2	The unmet timely diagnosis requirements, that take place years after subtantial neural loss and neuroperturbations in neuropsychaitric disorders, affirm the dire need for biomarkers with proven efficacy. In Parkinson's disease (PD), Mild Cognitive impairment (MCI), Alzheimers disease (AD) and psychaitric disorders, it is difficult to detect early symptoms, given their mild nature. We hypothesize that employing fine motor patterns, derived from natural interactions with keyboards, also knwon as keystroke dynamics, could translate classic finger dexterity tests from clinics to populations in-the-wild for timely diagnosis, yet, further evidence is required to prove this efficiency. We have searched PubMeD, MEDLINE, IEEEXplore, EBSCO and Web of Science for eligible diagnostic accuracy studies employing keystroke dynamics (index test) for the detection of neuropsychiatric disorders as the main target condition. We evaluated the diagnostic performance of keystroke dynamics across 41 studies published between 2014 and 2022, comprising 3791 PD patients, 254 MCI patients, and 374 psychiatric disease patients. Of these, 25 studies were included in univariate random-effect meta-analysis models for diagnostic performance assessment. Pooled sensitivity and specificity are 0.86 (95% Confidence Interval (CI): 0.82-0.90, I^2 = 79.49 %) and 0.83 (CI: 0.79 – 0.87, I^2 = 83.45%) for PD, 0.83 (95% CI: 0.65 – 1.00, I^2 = 79.10%) and 0.87 (95% CI: 0.80 – 0.93, I^2= 0%) for psychomotor impairment, and 0.85 (95% CI: 0.74-0.96, I^2= 50.39%) and 0.82 (95% CI: 0.70 – 0.94, I^2=87.73%) for MCI and early AD, respectively. Our subgroup analyses conveyed the diagnosis efficiency of keystroke dynamics for naturalistic self-reported data, and the promising performance of multimodal analysis of naturalistic behavioral data and deep learning methods in detecting disease-induced phenotypes. The meta-regression models showed the increase in diagnostic accuracy and fine motor impairment severity index with age and disease duration for PD and MCI. The risk of bias, based on the QUADAS-2 tool, is deemed low to moderate and overall, we rated the quality of evidence to be moderate. We conveyed the feasibility of keystroke dynamics as digital biomarkers for fine motor decline. In naturalistic environments. Future work to evaluate their performance for longitudinal disease monitoring and therapeutic implications is yet to be performed. We eventually propose a partnership strategy based on a "co-creation" approach that stems from mechanistic explanations of patients' characteristics derived from data obtained in-clinics and under ecologically valid settings. The protocol of this systematic review and meta-analysis is registered in PROSPERO; identifier CRD42021278707. The presented work is supported by the KU-KAIST joint research center.	Page 1
**INTRODUCTION**			
Rationale	3	Early diagnosis of neurological and psychiatric disorders is key for neuroprotection and disease modifying treatments. While motor abnormalities represent well-defined criteria in clinical gold-standards, recognition systems of preclinical, subtle motor decline are not yet integrated with the clinical workflow. Therefore, our systematic review and meta-analysis aims to provide a qualitative, and quantitative evidence on the role of a special class of digital biomarker, that of typing patterns, in early detection of fine motor decline mainly in Parkinson's Disease (PD), and extend the analysis to Mild Cognitive Impairment and psychiatric disorders.	Page 3

PRISMA 2020 Checklist

Section and Topic	Item #	Checklist item	Location where item is reported
Objectives	4	The objectives of the current systematic review and meta-analysis are related to the following Research Questions (RQs):<br>• RQ1: Do passively collected keyboard interactions, particularly the keystroke dynamics, result in clinically and ecologically valid diagnostic performance with reproducibility and repeatability in neurology and psychiatry?<br>• RQ2: What is the impact of data collection settings and the characteristics of the classification methods on the diagnostic sensitivity, specificity, and accuracy?<br>• RQ3: Can passively collected keyboard interactions, inform disease progression and therapeutic decisions?<br>• RQ4: With the aim of enhancing compatibility with the clinical workflow, what should the direction of future research be?	Page 3
**METHODS**			
Eligibility criteria	5	**No restrictions on language or publication date were applied.**<br>**Inclusion Criteria:**<br>-Case-Control Studies assessing the diagnostic performance of keyboard typing patterns, and features extracted from them to represent the fine motor decline in diseased groups compared to healthy controls.<br>-No restrictions on date or language are imposed.<br>**Exclusion Criteria:**<br>-Review articles or Meta-analysis<br>-Conference Abstracts<br>-Preprints and not peer reviewed papers.<br>-Studies eliciting emotional or cognitive distress in healthy participant groups and comparing neutral to stress states.	Page 14
Information sources	6	A systematic search of PubMed, IEEE Xplore, Web of Science, and EBSCO has been performed by two independent authors (H.A and N.C) between January 2021 and March 2022, without restrictions on language or publication date. The detailed outcome of the search was handled through Rayyan web of intelligent systematic reviews.	Page 14
Search strategy	7	Present the full search strategies for all databases, registers and websites, including any filters and limits used.<br>We conducted electronic searches of the databases up to March 25th, 2020. The following databases were searched:<br>• MEDLINE<br>• PubMed	Page 14, Pages S2-S7

Section and Topic	Item #	Checklist item	Location where item is reported
		• IEEE Xplore<br>• Web of Science<br>• EBSCO<br>The references of the included studies were also screened for eligible studies.<br>The search strategies for each database are as follows:<br>MEDLINE:	

Search Terms	Number of Results
Digital biomarkers	550
Digital Phenotyping	330
Digital Biomarkers AND Neurology	92
Digital phenotyping AND Neurology	15
Digital Biomarkers AND Psychiatry	74
Digital phenotyping AND Psychiatry	151
Digital Biomarkers AND Parkinson's Disease	42
Digital Phenotyping AND Parkinson's Disease	4
Digital Biomarkers AND [cognitive impairment OR cognitive dysfunction OR cognitively impaired]	36
Digital Phenotyping AND [cognitive impairment OR cognitive dysfunction OR cognitively impaired]	7
Digital Biomarkers AND [psychiatric disorders OR mental disorder OR mental illness OR psychiatric disorder]	16
Digital Phenotyping AND [psychiatric disorders OR mental disorder OR mental illness OR psychiatric disorder]	44
Keystroke dynamics NOT Security NOT Authentication	26
[keystroke dynamics OR smartphone typing OR keyboard typing] AND [parkinson's disease OR parkinson disease OR parkinsons disease OR pd OR parkinsons OR parkinsonism]	8
[keystroke dynamics OR smartphone typing OR keyboard typing] AND [cognitive impairment OR cognitive dysfunction OR cognitively impaired]	1

Section and Topic	Item #	Checklist item	Location where item is reported

[keystroke dynamics OR smartphone typing OR keyboard typing] AND [depression or depressive disorder or depressive symptoms or major depressive disorder]	7
[keystroke dynamics OR smartphone typing OR keyboard typing] AND [bipolar disorder or bipolar i or bipolar ii or manic depression or bipolar affective disorder or bipolar depression]	3

IEEE XPLORE Search:

Search Terms	Number of Results
Digital biomarkers	195
Digital Phenotyping	53
Digital Biomarkers AND Neurology	4
Digital Biomarkers AND Psychiatry	2
Digital phenotyping AND Psychiatry	4
Digital Biomarkers AND Parkinson’s Disease	7
Digital Phenotyping AND Parkinson’s Disease	4
Digital Biomarkers AND [cognitive impairment OR cognitive dysfunction OR cognitively impaired]	356
Digital Phenotyping AND [cognitive impairment OR cognitive dysfunction OR cognitively impaired]	7
Keystroke dynamics NOT Security NOT Authentication	57
[keystroke dynamics OR smartphone typing OR keyboard typing] AND [parkinson's disease OR parkinson disease OR parkinsons disease OR pd OR parkinsons OR parkinsonism]	571
[keystroke dynamics OR smartphone typing OR keyboard typing] AND [cognitive impairment OR cognitive dysfunction OR cognitively impaired]	401
[keystroke dynamics OR smartphone typing OR keyboard typing] AND [depression or depressive disorder or depressive symptoms or major depressive disorder]	267

Section and Topic	Item #	Checklist item	Location where item is reported

[keystroke dynamics OR smartphone typing OR keyboard typing] AND [bipolar disorder or bipolar i or bipolar ii or manic depression or bipolar affective disorder or bipolar depression]	301

Web of Science:

Search Terms	Number of Results
Digital biomarkers AND neurology	53
Digital Phenotyping AND neurology	11
Digital Biomarkers AND Psychiatry	175
Digital phenotyping AND Psychiatry	196
Digital Biomarkers AND Parkinson's Disease	66
Digital Phenotyping AND Parkinson's Disease	10
Digital Biomarkers AND [cognitive impairment OR cognitive dysfunction OR cognitively impaired]	113
Digital Phenotyping AND [cognitive impairment OR cognitive dysfunction OR cognitively impaired]	19
Digital Biomarkers AND [psychiatric disorders OR mental disorder OR mental illness OR psychiatric disorder]	126
Keystroke dynamics NOT Security NOT Authentication	231
[keystroke dynamics OR smartphone typing OR keyboard typing] AND [parkinson's disease OR parkinson disease OR parkinsons disease OR pd OR parkinsons OR parkinsonism]	6
[keystroke dynamics OR smartphone typing OR keyboard typing] AND [cognitive impairment OR cognitive dysfunction OR cognitively impaired]	1
[keystroke dynamics OR smartphone typing OR keyboard typing] AND [depression or depressive disorder or depressive symptoms or major depressive disorder]	7
[keystroke dynamics OR smartphone typing OR keyboard typing] AND [bipolar disorder or bipolar i or bipolar ii or manic depression or bipolar affective disorder or bipolar depression]	7

Section and Topic	Item #	Checklist item	Location where item is reported

PubMed:

Search Terms	Number of Results
Digital Biomarkers AND Neurology	260
Digital phenotyping AND Neurology	141
Digital Biomarkers AND Psychiatry	181
Digital phenotyping AND Psychiatry	254
Digital Biomarkers AND Parkinson's Disease	82
Digital Phenotyping AND Parkinson's Disease	26
Digital Biomarkers AND [cognitive impairment OR cognitive dysfunction OR cognitively impaired]	61
Digital Phenotyping AND [cognitive impairment OR cognitive dysfunction OR cognitively impaired]	8
Digital Biomarkers AND [psychiatric disorders OR mental disorder OR mental illness OR psychiatric disorder]	165
Digital Phenotyping AND [psychiatric disorders OR mental disorder OR mental illness OR psychiatric disorder]	251
Keystroke dynamics NOT Security NOT Authentication	42
[keystroke dynamics OR smartphone typing OR keyboard typing] AND [parkinson's disease OR parkinson disease OR parkinsons disease OR pd OR parkinsons OR parkinsonism]	7
[keystroke dynamics OR smartphone typing OR keyboard typing] AND [cognitive impairment OR cognitive dysfunction OR cognitively impaired]	2
[keystroke dynamics OR smartphone typing OR keyboard typing] AND [depression or depressive disorder or depressive symptoms or major depressive disorder]	4
[keystroke dynamics OR smartphone typing OR keyboard typing] AND [bipolar disorder or bipolar i or bipolar ii or manic depression or bipolar affective disorder or bipolar depression]	301

EBSCO:

Section and Topic	Item #	Checklist item	Location where item is reported
		(search terms table below)	
Selection	8	Specify the methods used to decide whether a study met the inclusion criteria of the review, including how many reviewers screened each record	Page 14

Search Terms	Number of Results
Digital Biomarkers AND Neurology	626
Digital phenotyping AND Neurology	55
Digital Biomarkers AND Psychiatry	407
Digital phenotyping AND Psychiatry	362
Digital Biomarkers AND Parkinson's Disease	233
Digital Phenotyping AND Parkinson's Disease	16
Digital Biomarkers AND [cognitive impairment OR cognitive dysfunction OR cognitively impaired]	259
Digital Phenotyping AND [cognitive impairment OR cognitive dysfunction OR cognitively impaired]	13
Digital Biomarkers AND [psychiatric disorders OR mental disorder OR mental illness OR psychiatric disorder]	265
Digital Phenotyping AND [psychiatric disorders OR mental disorder OR mental illness OR psychiatric disorder]	182
Keystroke dynamics NOT Security NOT Authentication	852
[keystroke dynamics OR smartphone typing OR keyboard typing] AND [parkinson's disease OR parkinson disease OR parkinsons disease OR pd OR parkinsons OR parkinsonism]	18
[keystroke dynamics OR smartphone typing OR keyboard typing] AND [cognitive impairment OR cognitive dysfunction OR cognitively impaired]	4
[keystroke dynamics OR smartphone typing OR keyboard typing] AND [depression or depressive disorder or depressive symptoms or major depressive disorder]	12
[keystroke dynamics OR smartphone typing OR keyboard typing] AND [bipolar disorder or bipolar i or bipolar ii or manic depression or bipolar affective disorder or bipolar depression]	11

Section and Topic	Item #	Checklist item	Location where item is reported
process		and each report retrieved, whether they worked independently, and if applicable, details of automation tools used in the process.<br>The handling of the search results was carried out on Rayyan web of intelligent systematic reviews by three reviewers (H.A, A.K and L.H).<br>Since we are targeting diagnostic accuracy studies of keystroke dynamics for neuropsychiatric disorder, we have automatically excluded papers for security and authentication purposes, as well as those on affective computing, stress detection and emotion recognition.	
Data collection process	9	Specify the methods used to collect data from reports, including how many reviewers collected data from each report, whether they worked independently, any processes for obtaining or confirming data from study investigators, and if applicable, details of automation tools used in the process.<br>Two reviewers (H.A. and L.H) independently collected data from the studies. No automation tools were used for data collection.	Page 14
Data items	10a	**List and define all outcomes for which data were sought. Specify whether all results that were compatible with each outcome domain in each study were sought (e.g. for all measures, time points, analyses), and if not, the methods used to decide which results to collect.**<br>For the meta-analysis, the sensitivity, specificity, accuracy and AUC were used in a random-effects model employing the meta-essential tool. In the case of available data on true positives (TP), true negatives (TN), false positives (FP) and false negatives (FN), the pooled measures were evaluated as follows:<br>Sensitivity = TP / (TP+FN)<br>Specificity = TN / (TN+FP)<br>Accuracy= (TP+TN) / (TP+TN+FN+FP)	Page 14
	10b	**List and define all other variables for which data were sought (e.g. participant and intervention characteristics, funding sources). Describe any assumptions made about any missing or unclear information.**<br>The following data were extracted from the studies for systematic evaluation:<br>(1) disease, (2) first author and publication year, (3) experimental protocol of data collection including collection settings and study duration, (4) number and mean age of participants in diseased and healthy groups, (5) data labeling methodology (self-reported meta-data vs. clinical evaluation), (6) data streams employed by the study, (7) extracted features, (8) analysis and feature extraction level (subject- level vs. typing session-level), (9) problem formulation and validation whether through statistical analyses or classification, (10) 2 x 2 data (True Positives, True negatives, False Positives, False Negatives), and from here we extracted the sensitivity and the specificity (11) Classification Accuracy and (12) Area Under the Receiver Operating Characteristics Curve (AUC).	Page 14
Study risk of bias assessment	11	**Specify the methods used to assess risk of bias in the included studies, including details of the tool(s) used, how many reviewers assessed each study and whether they worked independently, and if applicable, details of automation tools used in the process.**<br>Two reviewers (H.A. and L.H. independently assessed the risk of bias for all included studies using the QUADAS-2 tool.	Page 14

Section and Topic	Item #	Checklist item	Location where item is reported
Effect measures	12	**Specify for each outcome the effect measure(s) (e.g. risk ratio, mean difference) used in the synthesis or presentation of results.**<br>The diagnosis accuracy, sensitivity, specificity and area under the Receiver Operating Characteristic Curve (AUC) were pooled.	Page 15
Synthesis methods	13a	**Describe the processes used to decide which studies were eligible for each synthesis (e.g. tabulating the study intervention characteristics and comparing against the planned groups for each synthesis (item #5)).**<br>The outcomes of the meta-analysis were the Area Under the receiver operating characteristic Curve (AUC), accuracy, sensitivity and specificity. These outcomes were pooled and included in a univariate random effect model independently for three disease categories, namely PD, MCI, and psychiatric disorders. Heterogeneity was assessed using the I^2 statistics, attributable to non-sample related between-studies differences, in addition to the Cochran Q (X^2) test ($p<0{\cdot}05$). Given that in this study we report the validity of keystroke dynamics models as diagnostic tools for different disorders, we accepted high heterogeneity ($I^2 > 50\%$).	Page 15
	13b	All missing data were handled as stated in item 10b. Studies not reporting diagnostic accuracy measures were not included in the meta-analysis but in the systematic review.	
	13c	**Describe any methods used to tabulate or visually display results of individual studies and syntheses.**<br>Forest plots for pooled sensitivity, specificity, AUC and accuracy and their 95% confidence interval.<br>Scatter bar plots for subgroup analysis results.<br>Characteristics of included studies table	Pages 27-60
	13d	**Describe any methods used to synthesize results and provide a rationale for the choice(s). If meta-analysis was performed, describe the model(s), method(s) to identify the presence and extent of statistical heterogeneity, and software package(s) used.**<br>The random effects meta-analysis model was performed using the MetaEssential Tool.	Pages 15-16
	13e	**Describe any methods used to explore possible causes of heterogeneity among study results (e.g. subgroup analysis, meta-regression).**<br>The following subgroup analyses were performed:<br>• Data in the clinic and in the wild<br>• Clinical assessment and self-reports<br>• Multimodal and unimodal analysis<br>• Deep learning and other machine learning models.<br><br>Meta-regression analysis was performed to assess the relationship between:<br>(1) The age and disease duration of PD patients<br>(2) The disease duration and diagnostic AUC of PD patients<br>(3) The age and diagnosis AUC of MCI patients	

Section and Topic	Item #	Checklist item	Location where item is reported
	13f	**Describe any sensitivity analyses conducted to assess robustness of the synthesized results.**<br>We performed sensitivity analysis employing "leave-one-study-out" to ensure that the reported effect sizes are not influenced by the results of one study.	
Reporting bias assessment	14	**Describe any methods used to assess risk of bias due to missing results in a synthesis (arising from reporting biases).**<br>quality assessment was performed employing the tool for Quality Assessment of Diagnostic Test Accuracy (QUADAS-2)	Page 16
Certainty assessment	15	Outcome certainty assessment and quality of evidence was evaluated using the Grades of Recommendations, Assessment, Development and Evaluation (GRADE) tool.	Page 16
**RESULTS**			
Study selection	16a	**Describe the results of the search and selection process, from the number of records identified in the search to the number of studies included in the review, ideally using a flow diagram.**	Page 38

Section and Topic	Item #	Checklist item	Location where item is reported

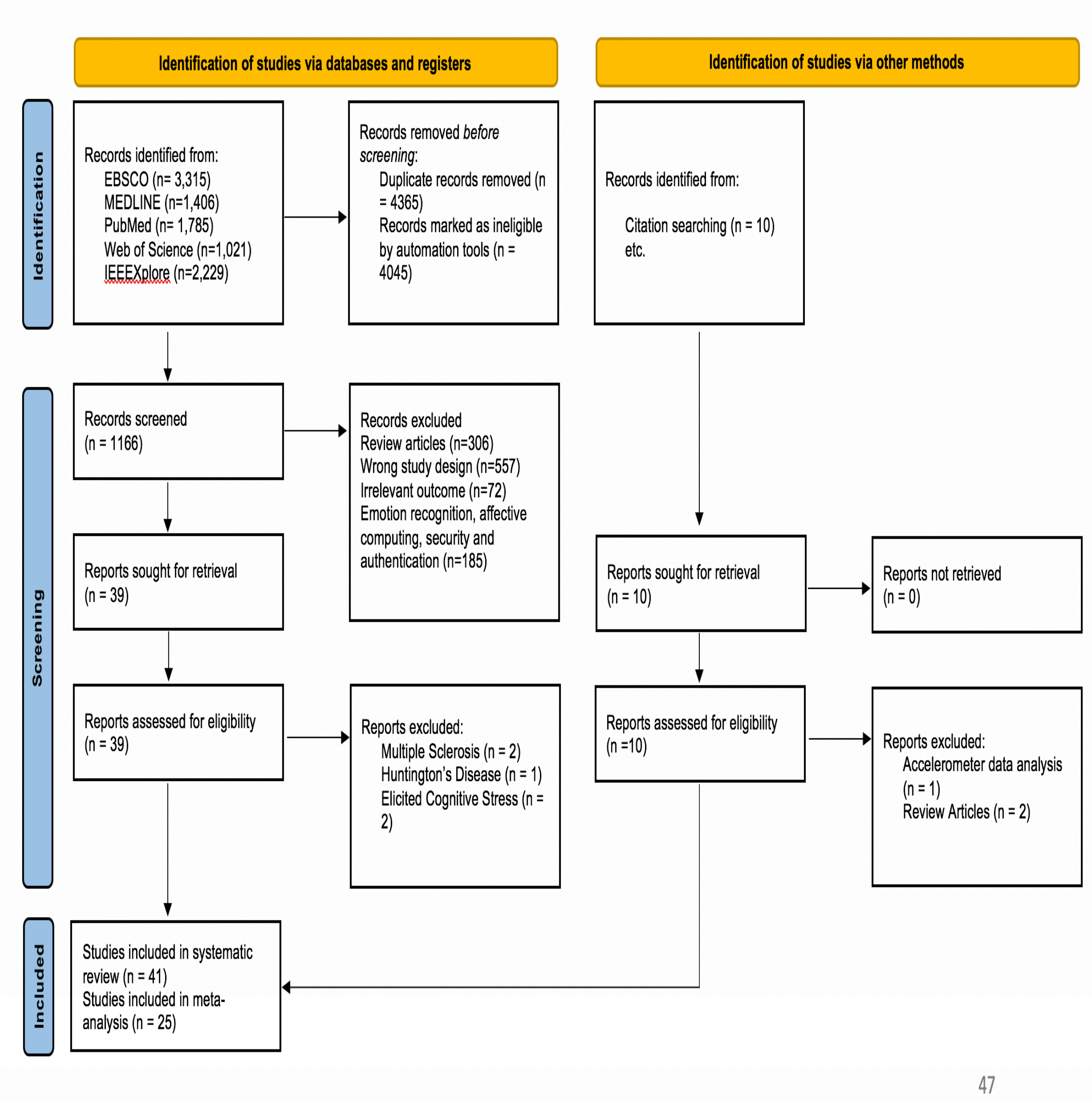

	16b	**Cite studies that might appear to meet the inclusion criteria, but which were excluded, and explain why they were excluded.**	Page S7

Section and Topic	Item #	Checklist item	Location where item is reported
		We excluded two full articles on Multiple Sclerosis and one on Huntington's disease, due to the very few studies on these two disorders. We have also excluded two full texts because they elicited cognitive stress in participants, and did not recruit medically diagnosed patients. The citation of the aforementioned studies are as follows:<br>(1) Lam, K. H., Meijer, K. A., Loonstra, F. C., Coerver, E. M. E., Twose, J., Redeman, E., ... & Killestein, J. (2021). Real-world keystroke dynamics are a potentially valid biomarker for clinical disability in multiple sclerosis. *Multiple Sclerosis Journal*, *27*(9), 1421-1431.<br>(2) Twose, J., Licitra, G., McConchie, H., Lam, K. H., & Killestein, J. (2020). Early-warning signals for disease activity in patients diagnosed with multiple sclerosis based on keystroke dynamics, *Chaos: An Interdisciplinary Journal of Nonlinear Science*, *30*(11), 113133.<br>(3) Lang, C., Gries, C., Lindenberg, K. S., Lewerenz, J., Uhl, S., Olsson, C., ... & Landwehrmeyer, G. B. (2021). Monitoring the motor phenotype in Huntington's disease by analysis of keyboard typing during real life computer use. *Journal of Huntington's Disease*, *10*(2), 259-268.<br>(4) Vizer, L. M., & Sears, A. (2011, November). Detecting cognitive impairment using keystroke and linguistic features of typed text: toward an adaptive method for continuous monitoring of cognitive status. In *Symposium of the Austrian HCI and Usability Engineering Group* (pp. 483-500). Springer, Berlin, Heidelberg.<br>(5) Vizer, L. M. (2009). Detecting cognitive and physical stress through typing behavior. In *CHI'09 Extended Abstracts on Human Factors in Computing Systems* (pp. 3113-3116).	
Study characteristics	17	The full characteristics of all included studies are reported in Table 1 of the manuscript.	Pages 27-43
Risk of bias in studies	18	**Present assessments of risk of bias for each included study.**<br>To assess the internal validity of the included studies, quality assessment was performed employing the tool for Quality Assessment of Diagnostic Test Accuracy (QUADAS-2). All discrepancies were resolved by mutual discussions between three authors (H.A., A.K., and L.H.). The risk of bias is presented in Supplementary Table 6 of the supplementary file	Pages S10-S11
Results of individual studies	19	**For all outcomes, present, for each study: (a) summary statistics for each group (where appropriate) and (b) an effect estimate and its precision (e.g. confidence/credible interval), ideally using structured tables or plots.**<br>(a) The summary statistics of the studies reporting diagnostic metrics of keystroke dynamics are reported in Supplementary Table 7.<br>(b) The effect estimates along with their 95 % confidence intervals are reported in forest plots (Figures 2-4) in the main manuscript.	Pages 48-59<br><br>Pages S12-S13
Results of syntheses	20a	**For each synthesis, briefly summarise the characteristics and risk of bias among contributing studies.**<br>The overall risk of bias assessment is illustrated in Figure 7 of the main manuscript.	Pages 10 and 63
	20b	**Present results of all statistical syntheses conducted. If meta-analysis was done, present for each the summary estimate and its precision**	Pages 7-

Section and Topic	Item #	Checklist item	Location where item is reported
		**(e.g. confidence/credible interval) and measures of statistical heterogeneity. If comparing groups, describe the direction of the effect.**<br>• **Parkinson's Disease:**<br>Pooled AUC: 0.85 (95%CI: 0.83 – 0.88; I^2 = 94.04%)<br>Pooled Accuracy: 0.82 (95% CI: 0.78 – .86; I^2 = 71.55%)<br>Pooled Sensitivity: 0.86 (95% CI: 0.82 – 0.90, I^2 = 79.49 %)<br>Pooled Specificity: 0·83 (95% CI: 0.79 – 0.87, I^2 = 83.45%)<br>• **Mild Cognitive Impairment and Alzheimer's disease:**<br>Pooled AUC: 0.84 (95% CI: 0.78 – 0.90, I^2 = 87.43%)<br>Pooled Accuracy: 0.82 (95% CI: 0.74 – 0.89, I^2 = 72.63%)<br>Pooled Sensitivity: 0·85 (95% CI: .74 – 0.96, I^2 = 50.39%)<br>Pooled Specificity: 0.82 (95% CI: 0.70 – 0.94, I^2 = 87.73)<br>• **Psychiatric disease:**<br>Pooled AUC: 0.90 (95% CI: 0.82 – 0.97, I^2 = 0%)<br>Pooled Accuracy: 0.89 (95% CI: 0.83 – 0.95, I^2 = 35.56%)<br>Pooled Sensitivity: 0.83 (95% CI: 0.65 – 1.00, I^2 = 79.10%)<br>Pooled Specificity: 0.87 (95% CI: 0.80 – 0.93, I^2 = 0%)<br>• **Directions of effect inferred by the subgroup analyses:**<br>**(1)** Comparing the performance of the diagnostic models when per- formed on data captured in-the-clinic to data captured in-the-wild revealed that the AUC (p=0.007) and the accuracy (p=0.032) were significantly higher under clinical settings.<br>**(2)** The AUC (p=0.004), the accuracy (p=0.013), and the specificity (p=0.002) are significantly higher for clinically-validated databases, compared to self-reports labeled typing data.<br>(3) The specificity (p=0.042) and the accuracy (p=0.022), however, were significantly higher for multimodal analysis compared to unimodal analysis.<br>(4) Comparing the performance of ML classifiers and deep learning methods, the sensitivity was significantly higher for deep learning classifiers (p=0.029), compared to linear machine learning methods	9

Section and Topic	Item #	Checklist item	Location where item is reported
		• Meta-regression Results:<br>(1) The regression analysis yielded a statistically significant increase in AUC with disease duration (p=0.005), reflecting the progression of fine motor impairment skills of PD patients.<br>(2) there is a significant increase in the diagnostic AUC of MCI based on fine motor skills inferred by keystroke dynamics (p=0.017).	
	20c	**Present results of all investigations of possible causes of heterogeneity among study results.**<br>The possible sources of between-study heterogeneity are as follows:<br>(1) Demographic characteristics of patients (study participants)<br>(2) Clinical characteristics of patients (e.g., disease duration and medication status)<br>(3) Settings of data collection<br>(4) Methodologies of data labeling<br>(5) Modeling characteristics	Page 10
	20d	**Present results of all sensitivity analyses conducted to assess the robustness of the synthesized results.**<br>When performing sensitivity analysis to reveal the impact of individual studies on the overall effect size, we reported no significant influence by any of the studies on any of the diagnostic metrics of keystroke dynamics.	Page 8
Reporting biases	21	**Present assessments of risk of bias due to missing results (arising from reporting biases) for each synthesis assessed.**<br>We have assessed the publication bias by funnel plots (Figures S1-S4) in the supplementary material.	Pages S17-S20
Certainty of evidence	22	**Present assessments of certainty (or confidence) in the body of evidence for each outcome assessed.**<br>We have conducted the assessment of the quality of evidence using the GRADE tool as reported in supplementary table 8.	Pages S14-S16
**DISCUSSION**			
Discussion	23a	**Provide a general interpretation of the results in the context of other evidence.**<br>This review is the first to report the diagnostic performance of keystroke dynamics for detecting fine motor decline in PD, MCI and AD, and psychiatric disorders (i.e., psychomotor impairment). Furthermore, this review is the first to quantitively compare the diagnostic performance between (1) data collected in-the-clinic and data collected in-the-wild, (2) labeling methods of clinical evaluation and self-reports, (3) multimodal and unimodal analysis, and (4) deep learning and other machine learning classifiers. Besides early diagnosis, we qualitatively report the role of keystroke dynamics in informing therapeutic decisions, and longitudinal disease prognosis yet with sparse studies. From the findings of our review,	Page 10

Section and Topic	Item #	Checklist item	Location where item is reported
		we formulate an agenda for future work that stems from a co-creation approach, via the positive collaboration of clinicians, scientists and patients, towards a measurement-driven, multidisciplinary culture.	
	23b	**Discuss any limitations of the evidence included in the review.**<br>Typing patterns show promising potential as digital biomarkers for diagnosing and monitoring mood and cognitive decline. There is a disproportional focus towards Parkinson's disease; hence, further validation studies of these digital biomarkers in other disorders are still needed. Issues like intermittent clinical evaluation and validity in labeling across long time windows raise attention, placing the need for hybrid labeling (clinical assessment with Ecological Momentary Assessment). Latent domains and/or confounders (such as emotions, activity levels, and sleep patterns) should also be considered with keystroke dynamics, forming new integrated frameworks for capturing the heterogeneous, dynamic, neuropsychiatric symptoms in different behavioral disorders, both from inter- and intra-subject perspectives.	Page 12-13
	23c	**Discuss any limitations of the review processes used.**<br>is the sparsity and the inherent heterogeneity of the included studies. While we were able to perform regression analysis with patients' demographic and clinical characteristics (i.e., age, disease duration respectively) for PD, our meta-analysis lacks the investigation of additional covariates, such as gender differences and medication response, especially for MCI and psychiatric disorders. Although promising results have been revealed by leveraging typing patterns for diagnosing and monitoring mood and cognitive decline, the majority of the studies are, so far, disproportionately targeting PD. While this is understandable given the hallmark motor disturbance in this latter, we see that the need for further validations of this approach in other disorders is still pressing. This will be an important avenue for future studies.	Page 13
	23d	**Discuss implications of the results for practice, policy, and future research.**<br>we note the strength of our meta-analysis conclusions that conveyed the feasibility of using keystroke dynamics derived from the natural interaction connected devices keyboards as digital biomarkers for early decline in fine motor skills associated with neuropsychiatric disorders. Based on experimental design comparisons, we showed that the keystroke dynamics constitute an ecologically valid diagnostic platforms *in-the-wild*, reflecting their translational potentiality outside clinics, despite the methodological challenges that arises, including but limited to confounders influence and sampling difficulties. Further, given the influence of data labeling on the diagnosis models, we conclude that even when self-reported data *in-the-wild* are used for training, keystroke dynamics models still achieve sound discriminatory potential. From methodological perspectives, we show that employing multimodal and advanced deep learning models, which are at the high edge of the contemporary data science methodologies, offer promising opportunities for boosting the diagnostic accuracy, but with considerable heterogeneity across the studies. Consequently, the establishment of intricate and generalizable diagnostic models, that not only achieve accurate diagnosis, but are also sensitive to temporal change and symptom progression. To this end, our regression models showed the evolution of diagnosis AUC and fine motor impairment with age and disease duration for PD. We reperformed the regression analysis for MCI, and showed how the diagnostic AUC increases with age, reflecting the increasing fine motor impairment severity. In conclusion, the importance of digital technology also goes beyond the diagnostic yield, so once at-risk cohorts are identified, digital technologies can also be employed to reinforce behavior change and patients' empowerment, towards a sustained quality of life.	Page 13
**OTHER INFORMATION**			

PRISMA 2020 Checklist

Section and Topic	Item #	Checklist item	Location where item is reported
Registration and protocol	24a	The protocol of this systematic review and meta-analysis has been registered in PROSPERO with identifier CRD42021278707. We refer the reader to the supplementary file for a copy of the registered PROSPERO protocol.	Page 14
	24b	**Indicate where the review protocol can be accessed, or state that a protocol was not prepared.** The protocol registered with PROSPERO is attached as pdf.	Page 14
	24c	**Describe and explain any amendments to information provided at registration or in the protocol.** Not applicable	Page 14
Support	25	**Describe sources of financial or non-financial support for the review, and the role of the funders or sponsors in the review.** This work is supported by the Joint Research Center of Khalifa University of Science and Technology and the Korean Advanced Institute of Science and Technology, 8474000221 (KKJRC-2019-Health2) awarded to Ahsan Khandoker and Leontios Hadjileontiadis. The funder did not have any role in the analysis performed.	Page 16
Competing interests	26	The review authors have no competing interest and have nothing to declare.	Page 16
Availability of data, code and other materials	27	**Report which of the following are publicly available and where they can be found: template data collection forms; data extracted from included studies; data used for all analyses; analytic code; any other materials used in the review.** The search strategy and extracted data contributing to the meta-analysis is available in the appendix; any additional data are available on request from the corresponding author.	Page 16
